# Tuning excited-state-intramolecular-proton-transfer (ESIPT) process and emission by cocrystal formation: a combined experimental and theoretical study†
†Electronic supplementary information (ESI) available: Experimental details, characterization of cocrystals, and computational details and results. CCDC 1454754 and 1454755. For ESI and crystallographic data in CIF or other electronic format see DOI: 10.1039/c6sc04354b



**DOI:** 10.1039/c6sc04354b

**Published:** 2016-11-14

**Authors:** Heyang Lin, Xueping Chang, Dongpeng Yan, Wei-Hai Fang, Ganglong Cui

**Affiliations:** a Key Laboratory of Theoretical and Computational Photochemistry , Ministry of Education , College of Chemistry , Beijing Normal University , Beijing 100875 , P. R. China . Email: yandp@bnu.edu.cn ; Email: ganglong.cui@bnu.edu.cn ; Fax: +86-10-64425385 ; Tel: +86-10-64412131; b State Key Laboratory of Chemical Resource Engineering , Beijing University of Chemical Technology , Beijing 100029 , P. R. China

## Abstract

The formation of two-component molecular cocrystals can lead to the tunable excited state intramolecular proton transfer (ESIPT) process and emission, as first confirmed by both experimental and computational studies.

## Abstract 2

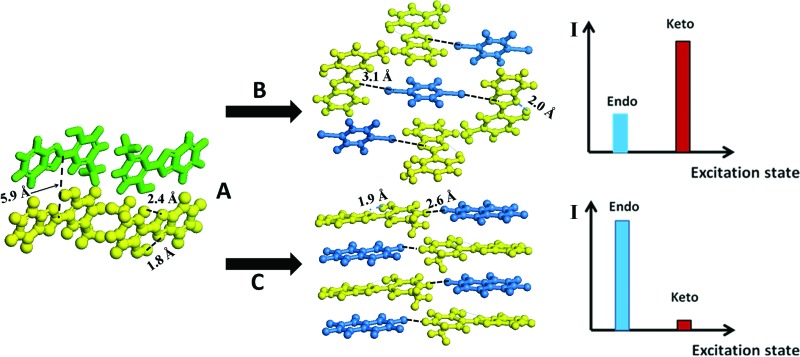
The formation of two-component molecular cocrystals can lead to the tunable excited state intramolecular proton transfer (ESIPT) process and emission, as first confirmed by both experimental and computational studies.

Molecular solid-state photoactive materials have been widely studied during the past two decades due to their excellent optoelectronic features in applications including light-emitting diodes (LEDs),^1^ lasers,^2^ and chemical/biological sensors.^3^ Recently, tuning molecular solid-state aggregation and the related optical/luminescence properties has been paid much attention because it not only paves an effective way to achieve tunable multicolor emission of molecule-based materials,^4^ but also can provide an understanding of the relationship between molecular arrangement and bulk optical properties.^5^ In this context, the formation of photofunctional cocrystals^6^ based on hybridization of target photoactive molecules with one or more assembled units has obtained increasing interest, because it clearly offers more flexibility through tailoring intermolecular non-covalent interactions (*e.g.* hydrogen- and halogen-bonding).

Recent developments in crystal engineering (also known as solid-state supramolecular chemistry) have largely enhanced our understanding of molecular self-assembly and intermolecular interactions.^7^ Furthermore, the synthesis of cocrystals is of great interest in the fields of molecule-based materials chemistry and crystal engineering, since the cocrystal materials can be commonly designed based on the principles of molecular recognition, such as the supramolecular synthon strategy in typical multicomponent crystal systems.^7*a*^ For example, the formation of cocrystals composed of active pharmaceutical ingredients (APIs) has been regarded as a promising route towards commercial products, since cocrystallization can be used to modify the solid-state properties of APIs (such as solubility, bioavailability, hydration/thermal stability, and mechanical behavior).^7b,c^ Also, cocrystallization supplied an effective way to tune the color and photoemission of molecular systems.^6*a*^ Applications of cocrystals have already been extended to the fields of ambipolar charge transport,^8^ photoconductivity,^9^ and nonlinear optics.^10^ Although solid-state supramolecular structures based on hydrogen and halogen bonding designs have been widely constructed, the development of efficient photofunctional cocrystals is still in an early stage, and examples are still relatively rare.^11*a*^ This is mainly due to the fact that the relationships between crystal arrangement/molecular configuration and optical/luminescent performance, as well as the detailed mechanism of electronic interaction between molecular building blocks, have not been fully understood.^11*b*^

Excited state intramolecular proton transfer (ESIPT) is a photochemical process involving a photo-induced enol–keto tautomerization leading to significant variations in the electronic structure and photophysical properties compared to the canonical form.^12^ In recent years molecular systems with ESIPT characteristics have attracted much attention in the areas of solar concentrators,^13^ optical memory,^14^ dual emitters,^15^ two-photon microscopy imaging,^16^ white LEDs,^17^ sunscreens,^18^ nucleobase photoprotection,^19^ and green fluorescent proteins,^20^ due to large Stokes shifted fluorescence emission.^21^ From both experimental and theoretical viewpoints, adjustment of the ESIPT process in the solid state plays an important role in tailoring photophysical properties and optoelectronic performance.^22^ It has been known that ESIPT-induced emission is highly dependent on solvent conditions;^23^ however, an effective strategy for tuning the ESIPT process and emission by designing and controlling suitable intermolecular interactions and related excitation states in a crystalline solid remains a long-standing problem. In this work, we have put forward a cocrystallization strategy to tune the solid state ESIPT process, the ratio of excited state enol–keto tautomerization, and related photophysical properties.

2-(2′-Hydroxy-5′-methyl-phenyl)-benzotriazole (denoted as UV-P hereinafter) is a well-known UV stabilizer and fluorescent material in chemical industry,^24^ which involves an ESIPT process from the O atom of the hydroxyl group (proton donor) to the N atom of the triazole unit (proton acceptor). Here, UV-P (**A**, Scheme 1) was selected as a model system to illustrate how to tailor its ESIPT process and excitation state in the solid-state based on changes in intermolecular interactions and molecular aggregation. Two typical co-assembled units (**B** and **C**, Scheme 1) with potential halogen/hydrogen bonds and π–π interactions with **A** were chosen to give rise to new UV-P-based cocrystals, which exhibited enhanced and reduced ESIPT emission respectively compared with the pristine UV-P. Moreover, high-level electronic structure calculations (DFT, TD-DFT, and MS-CASPT2) have further confirmed how the introduction of the coformer can influence the ESIPT process from a theoretical perspective. Therefore, this work supplied an effective way to obtain tunable ESIPT emission of molecular materials.

**Scheme 1:**
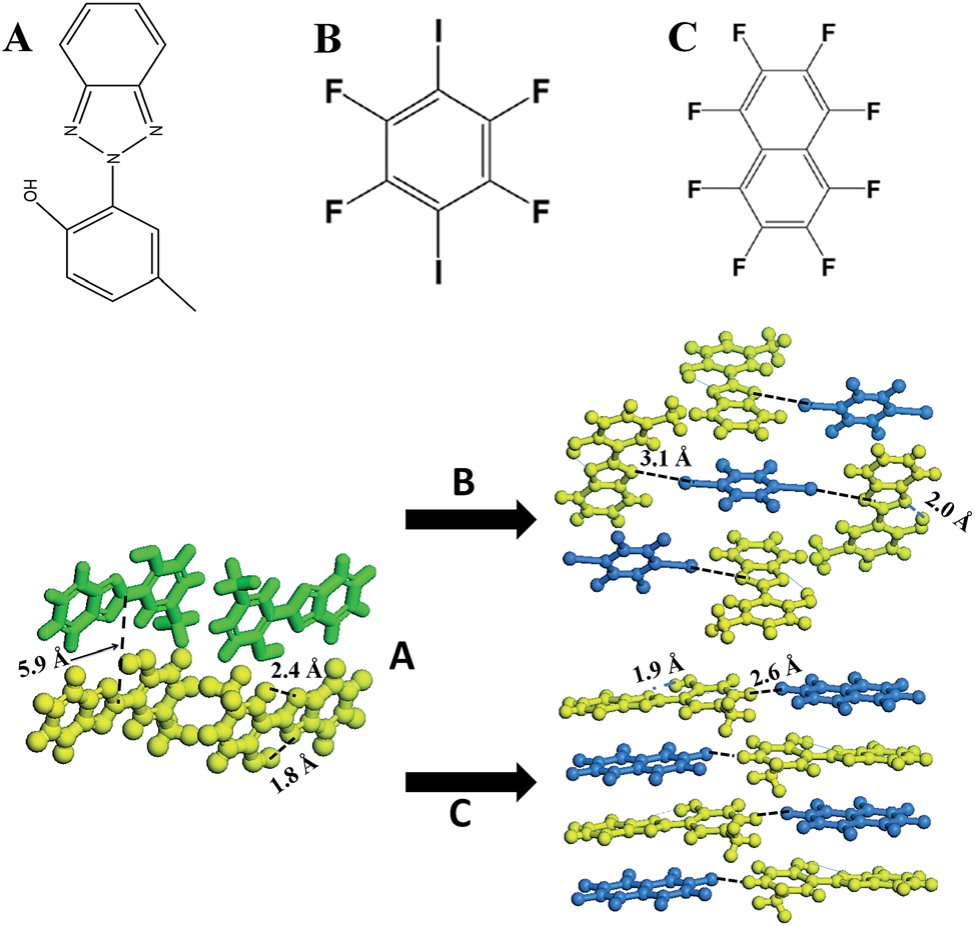
Molecular structures and self-assembly fashion of 2-(2′-hydroxy-5′-methyl-phenyl)-benzotriazole (UV-P, **A**) with 1,4-diiodotetrafluorobenzene (**B**) and octafluoronaphthalene (**C**) towards tuning excitation states between endo and keto forms.

For the pristine UV-P solid, the typical emission for the ESIPT process can be observed with two wavelength bands located at 410 and 604 nm (298 K, Fig. 1A); this solid-state ESIPT emissive characteristic is close to that of the solution form (Fig. S1†). In addition, the relative intensities of the two bands varied obviously with the temperature: with a decrease in the temperature, the intensity at 410 nm presents a slightly decreasing trend, while the one at 604 nm is highly increased. The ratiometric fluorescence intensity *I*_604_/*I*_410_ increases from 0.5 to 3.8 in the range from 298 to 98 K. The crystal structure shows that the UV-P molecules are highly isolated from each other (Scheme 1), suggesting that the intermolecular interactions may not play a key role in the fluorescence properties.

**Fig. 1:**
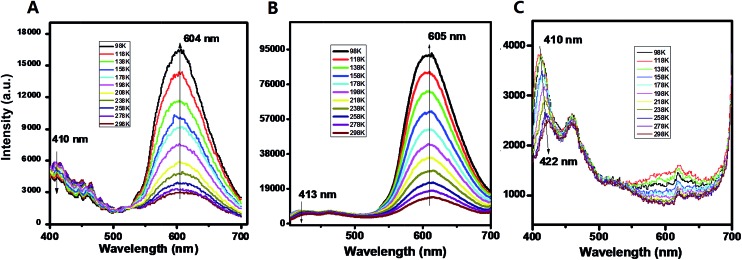
PL emission of UV-P (**A**) and its solid-state cocrystals **A.B.** and **A.C.** at different temperatures.

The powdered form of cocrystals of **A.B.** and **A.C.** can be prepared through a net-grinding method of **A** and **B** (**C**) with a 2 : 1 or 1 : 1 ratio. The single crystals were obtained by evaporation of the chloroform solutions of **A.B.** and **A.C.** precursor powders. Single crystal analysis (Fig. S2, S3 and Table S1†) showed that upon the assembly of **A** with **B** to obtain cocrystal **A.B.**, every two **A** molecules were assembled with one **B** molecule *via* an N···I halogen bond (distance: 3.1 Å), and the three molecules (**2A** and **B**) can be further regarded as a basic unit to form a 3D stacking as shown in Scheme 1. The adjacent **A** and **B** molecules are also organized based on the π–π interactions. For cocrystal **A.C.**, a layered mixed stacking fashion was formed through π–π interactions. Hydrogen bonding can also be observed between C–H in **A** and F–C in **C**. Powder XRD patterns of the grinding products are consistent with the simulated ones from the single crystal structures (Fig. S4†), suggesting the purity of the powdered cocrystal samples. Solid-state ^1^H nuclear magnetic resonance (Fig. S5 and S6†) and Fourier transform infrared spectra (Fig. S7†) confirmed that the H atom is attached to the hydroxyl group, suggesting no proton transfer in the ground state for **A.B.** and **A.C.**, which is consistent with the crystal structure results.

The two molecular cocrystals present obviously different two-wavelength emission compared with the pristine **A** (Fig. 1B and C). For cocrystal **A.B.**, the emission at *ca.* 605 nm is significantly stronger than the one at *ca.* 413 nm at room temperature (298 K). The *I*_605_/*I*_413_ increases from 2.8 to 15.3 as the temperature decreases from 298 K to 98 K (Fig. S8†). To obtain further insight into the photophysical properties and excited-state information of fluorescence for these solids, the fluorescence lifetimes of **A** and **A.B.** were experimentally measured. The fluorescence decay curve (monitored at 605 nm, Fig. S9†) of **A.B.** is faster than that of the pristine **A**, and the corresponding lifetimes of **A** and cocrystal **A.B.** are 183 ps and 78 ps respectively. The decay time is consistent with the time scale for the typical ESIPT process.^21^ For the cocrystal **A.C.**, it is experimentally observed that the emission at *ca.* 605 nm is much weaker at both high and low temperatures, whereas the emission at low-wavelength (410–422 nm ranging from 98 to 298 K) becomes the dominant band. Moreover, compared with the pristine **A** (PLQY: 3.2%), the PLQY of cocrystal **A.B.** and **A.C.** is slightly decreased with values of 2.7% and 1.3% respectively. These observations indicate that the formation of cocrystals with **B** and **C** can result in increasing and decreasing ESIPT bands from UV-P respectively. To the best of our knowledge, while cocrystallization has been previously used to optimize a topotactic solid-state photochemical reaction,^7*d*^ it was never applied before to tune solid-state ESIPT.

In our opinion, alternations of molecular conformation and arrangement, as well as electronic interactions between chromophores and coformers may result in different ESIPT behaviors. For the pristine **A**, and cocrystals **A.B.** and **A.C.**, no obvious molecular aggregation (such as H- or J-type aggregation) of UV-P appears, and thus the molecular arrangement may not play a major role compared with systems with strong π–π stacking.^11^ Then, the possible conformational change of UV-P has been further understood by compiling the typical conformational parameters (Table S2†). It was observed that these parameters are very close for the pristine **A** and **A.B.**; however, for **A.C.**, the dihedral angle between the phenyl and benzotriazole groups has decreased relative to that of the pristine **A**, indicating that the coplanarity of **A** has been reduced upon intermolecular interaction with **C**. The change of coplanarity is consistent with the largely decreased ESIPT emission and quantum yield. Moreover, it was documented that fluorine-containing compounds could modify the optical/luminescent properties of aromatic compounds in the solid state.^6^ In this work, the interactions between the fluoride coformers and UV-P may also adjust the electronic structures and ESIPT photoemission.

In order to rationalize the distinct excited-state properties among crystal **A** and cocrystals **A.B.** and **A.C.**, we have employed *ab initio* electronic structure methods to explore the excited-state properties of **A**, **A.B.**, and **A.C.** (see ESI† for computational details). On the basis of the computational results, the experimentally observed emission bands around 410 nm of **A**, **A.B.**, and **A.C.** are assigned to the fluorescence emission of their respective S_1_ enol minima. The vertical S_1_ → S_0_ emission energies of these enol minima are computed to be 398 nm (71.9 kcal mol^–1^), 398 nm (71.8 kcal mol^–1^), and 438 nm (65.2 kcal mol^–1^) for **A**, **A.B.**, and **A.C.**, respectively, which agree well with the experimental data (see Table 1). By contrast, experimentally observed emission bands of *ca.* 600 nm stem from the fluorescence emission of the S_1_ keto species. At these keto minima, the vertical S_1_ → S_0_ emission energies are predicted to be 557 (51.3), 571 (50.1), and 604 nm (47.3 kcal mol^–1^) for **A**, **A.B.**, and **A.C.**, respectively.

**Table 1:** MS-CASPT2(10,8)/ONIOM(QM/QM′) computed fluorescence emission bands (in kcal mol^–1^ and nm) of **A**, **A.B.**, and **A.C.** (see ESI for details)

	Enol		Keto	
	kcal mol^–1^ (nm)	Exp.	kcal mol^–1^ (nm)	Exp.
**A**	71.9 (398)	69.7 (410)	51.3 (557)	47.3 (604)
**A.B.**	71.8 (398)	69.2 (413)	50.1 (571)	47.3 (605)
**A.C.**	65.2 (438)	69.7 (410)	47.3 (604)	46.4 (617)

In terms of the computed relative energies of the S_1_ enol and keto species, the intensity changes of the two emission bands of **A** and **A.B.** can be qualitatively understood. The S_1_ keto species of **A** and **A.B.** are more stable than the S_1_ enol ones by 6.4 and 5.2 kcal mol^–1^, respectively, so the S_1_ keto species are populated much more than the S_1_ enol species in the S_1_ state. Therefore, it is safe to expect that emission from the S_1_ keto species is much more intensive than that from the S_1_ enol species. In addition, the ESIPT process of **A.B.** is computed to be barrierless (Fig. 2), so its S_1_ enol species is populated very little in the S_1_ state. As a result, the emission band of *ca.* 400 nm of **A.B.** nearly disappears in the experiment (Fig. 1B). In contrast to **A.B.**, there is a small barrier of *ca.* 3.6 kcal mol^–1^ separating both S_1_ enol and keto species of the **A** molecular crystal; therefore, a weak emission band of *ca.* 400 nm is still observed (Fig. 1A). This could qualitatively rationalize that our experiments observe that *I*_605_/*I*_413_ of **A.B.** is significantly larger than the *I*_604_/*I*_410_ of **A** (Fig. S8†).

**Fig. 2:**
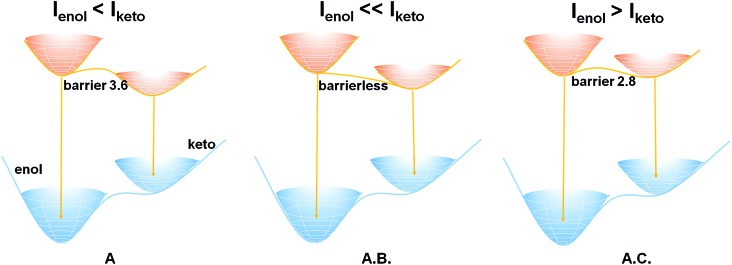
Schematic energy levels of the S_1_ enol and keto species of **A**, **A.B.**, and **A.C.** molecular crystal and cocrystals. In **A**, there is a small barrier for the ESIPT process; in **A.B.**, the ESIPT process is nearly barrierless; in **A.C.**, a small barrier is also associated with the ESIPT process. See text for detailed discussion.

Our computations also show that the S_1_ keto species of **A.C.** is able to fluoresce. Its S_1_ → S_0_ vertical emission energy is computed to be 604 nm (47.3 kcal mol^–1^). The population of the S_1_ keto species of **A.C.** is also comparable with its S_1_ enol species because their energy difference is 1.3 kcal mol^–1^. However, the S_1_ → S_0_ oscillator strength of the S_1_ enol species is about 3 times larger than that of the S_1_ keto species (0.6894 *versus* 0.2313). This could probably explain why the intensity of the emission band of 410 nm of **A.C.** is around 3–4 times stronger than that of the 600 nm emission band (Fig. 1C).

In summary, we have experimentally and computationally demonstrated how the formation of two-component crystalline solids can adjust and control the solid-state ESIPT process and emission (blue/red regions) based on tuning the intermolecular interactions and aggregation states. Introduction of suitable coformers shapes excited-state potential energy profiles related to ESIPT and thus results in highly enhanced or reduced ESIPT emission. We believe that the present strategy of tuning intermolecular interactions *via* the formation of molecular cocrystals can be further extended to other systems towards adjustable ESIPT emission for future potential luminescence, white-light generation, biology imaging, and energy applications.

## References

[cit1] Tang C. W., Van Slyke S. A. (1987). Appl. Phys. Lett..

[cit2] Zhao Y. S., Di C., Yang W., Yu G., Liu Y., Yao J. (2006). Adv. Funct. Mater..

[cit3] Che Y., Zang L. (2009). Chem. Commun..

[cit4] Varghese S., Das S. (2011). J. Phys. Chem. Lett..

[cit5] Sagara Y., Kato T. (2009). Nat. Chem..

[cit6] Yan D. P., Evans D. G. (2014). Mater. Horiz..

[cit7] Desiraju G. R. (1995). Angew. Chem., Int. Ed..

[cit8] Zhang J., Tan J., Ma Z., Xu W., Zhao G., Geng H., Di C., Hu W., Shuai Z., Singh K., Zhu D. (2013). J. Am. Chem. Soc..

[cit9] Zhu W., Zheng R., Fu X., Fu H., Shi Q., Zhen Y., Dong H., Hu W. (2015). Angew. Chem., Int. Ed..

[cit10] Yan D., Yang H., Meng Q., Lin H., Wei M. (2014). Adv. Funct. Mater..

[cit11] Yan D., Delori A., Lloyd G. O., Friscic T., Day G. M., Jones W., Lu J., Wei M., Evans D. G., Duan X. (2011). Angew. Chem., Int. Ed..

[cit12] Goodman J., Brus L. E. (1978). J. Am. Chem. Soc..

[cit13] Chen D.-Y., Chen C.-L., Cheng Y.-M., Lai C.-H., Yu J.-Y., Chen B.-S., Hseish C.-C., Chen H.-C., Chen L.-Y., Wei C.-Y., Wu C.-C., Chou P.-T. (2010). ACS Appl. Mater. Interfaces.

[cit14] Kim S., Park S. Y. (2003). Adv. Mater..

[cit15] Azarias C., Budzák Š., Laurent A. D., Ulrich G., Jacquemin D. (2016). Chem. Sci..

[cit16] Pascal S., Denis-Quanquin S., Appaix F., Duperray A., Grichine A., Guennic B. L., Jacquemin D., Cuny J., Chi S.-H., Perry J. W., van der Sanden B., Monnereau C., Andraud C., Maury O. (2016). Chem. Sci..

[cit17] Chen K.-Y., Hsieh C.-C., Cheng Y.-M., Lai C.-H., Chou P.-T. (2006). Chem. Commun..

[cit18] Shemesh D., Sobolewski A., Domcke W. (2009). J. Am. Chem. Soc..

[cit19] Lu Y., Lan Z., Thiel W. (2011). Angew. Chem., Int. Ed..

[cit20] Cui G., Lan Z., Thiel W. (2012). J. Am. Chem. Soc..

[cit21] Padalkar V. S., Seki S. (2016). Chem. Soc. Rev..

[cit22] Chou P.-T., Pu S.-C., Cheng Y.-M., Yu W.-S., Yu Y.-C., Hung F.-T., Hu W.-P. (2005). J. Phys. Chem. A.

[cit23] Abou-Zied O. K., Jimenez R., Thompson E. H. Z., Millar D. P., Romeberg F. E. (2002). J. Phys. Chem. A.

[cit24] Keck J., Kramer H. E. A., Port H., Hirsch T., Fischer P., Rytz G. J. (1996). J. Phys. Chem..

