# Supplementary material for: Tuning excited-state-intramolecular-proton-transfer (ESIPT) process and emission by cocrystal formation: a combined experimental and theoretical study
Source: Chem Sci. 2016 Nov 14;8(3):2086–90. doi: 10.1039/c6sc04354b (PMC5399640; doi:10.1039/c6sc04354b)
Supplement: Supplementary file 1 [file SC-008-C6SC04354B-s001.pdf]

Electronic Supplementary Material (ESI) for Chemical Science.
This journal is © The Royal Society of Chemistry 2016

Supporting Information:

Tuning Excited-State Intramolecular Proton Transfer (ESIPT) Process and Emission by the Cocrystal Formation: A Combined Experimental and Theoretical Study

Heyang Lin, Xue-Ping Chang, Dongpeng Yan*, Wei-Hai Fang, and Ganglong Cui*

Key Laboratory of Theoretical and Computational Photochemistry, Ministry of Education, College of Chemistry, Beijing Normal University, Beijing 100875, China

Contents

1. Experimental parts
2. Computational parts

Experimental Parts:

Materials. All the compounds were purchased from Sigma Chemical. Co. Ltd. and used without further purification.

Characterization: Single-crystal X-ray diffraction data of all compounds was collected on a Bruker SMART APEX CCD diffractometer equipped with graphite monochromatized Mo-Kα radiation (λ = 0.71073 Å) using the ω-scan technique. Photoluminescence (PL), PL quantum yield (PLQY), PL lifetime and temperature-dependent PL experiments were conducted on an Edinburgh FLS980 fluorescence spectrometer.

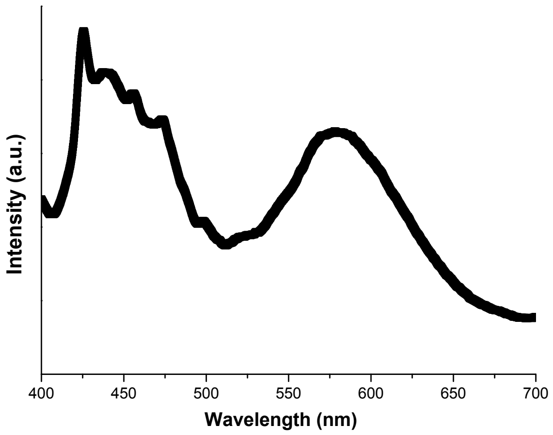

Figure S1. Fluorescence spectra of the form of pristine UV-P in chloroform solution (10^{-5} mol/L).

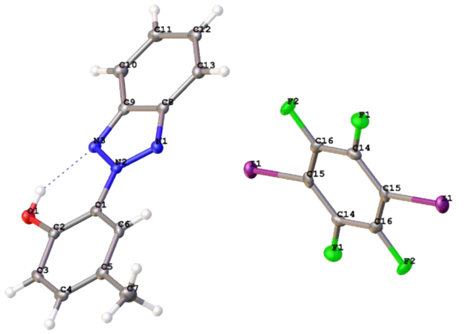

Figure S2. Crystal graphic of **A.B.**

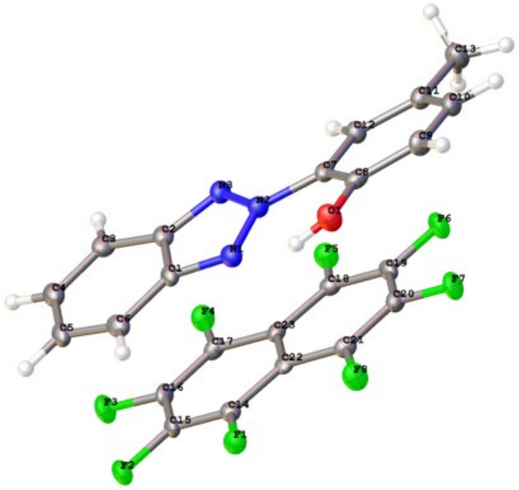

Figure S3. Crystal graphic of **A.C.**

Table S1. Crystal structure information of UV-P cocrystals

	**AB (CCDC: 1454755)**	**AC (CCDC: 1454754)**
Formula	$C_{32}H_{22}F_4I_2N_6O_2$	$C_{23}H_{11}F_8N_3O$
Formula weight	852.36	497.35
Temperature/K	97.20(10)	97.3
Crystal system	monoclinic	monoclinic
Space group	$P2_1/c$	$P2_1/c$
a(Å)	15.842(8)	6.5595(9)
b(Å)	5.6980(5)	41.849(3)

c(Å)	16.7517(15)	6.9922(5)
α(deg)	90.00	90.00
β(deg)	94.72(2)	94.939(8)
γ(deg)	90.00	90.00
Z	2	4
2θ range	6.8 to 51.98°	5.92 to 51.98°
D_c/g·cm^{-1}	1.878	1.727
F(000)	828	1000
R_{int}	0.0381	0.0486
GOF	1.028	1.090
R_1[I>2σ(I)]	0.0332	0.0642
wR_2(all data)	0.0753	0.1452
ρ_{max}/ρ_{min}(eÅ^{-3})	0.642/-0.620	0.274/-0.361

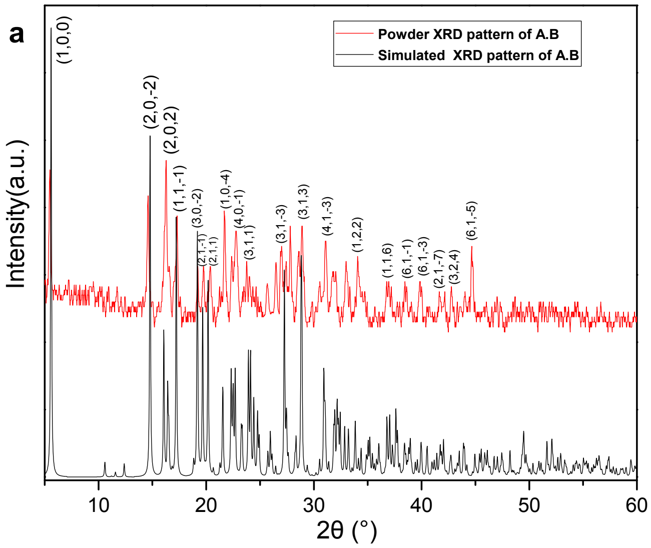

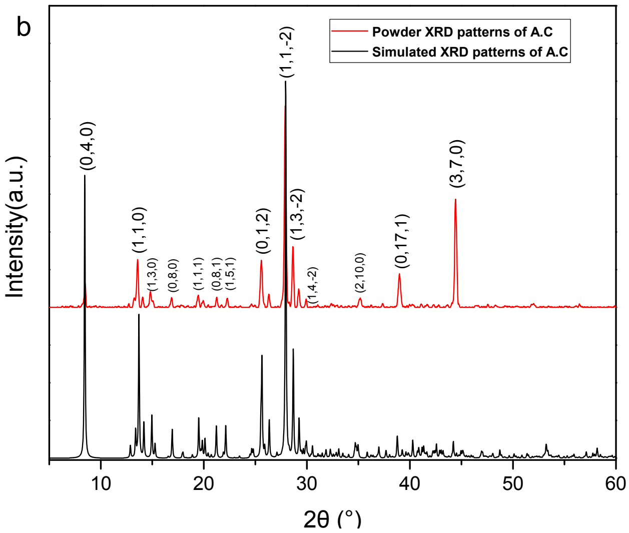

Figure S4. Simulated powder XRD patterns calculated from the crystallographic data of **A.B.** (black line) and measured powder XRD patterns (red line) of the **A.B.** (left) and **A.C.** powders (right).

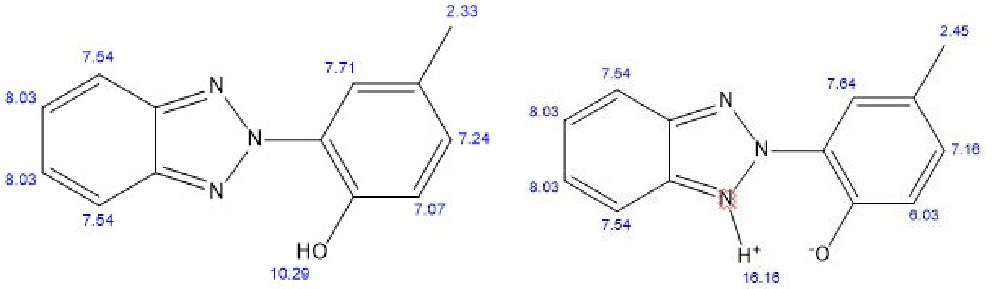

Figure S5. The predicted solid-state ^{1}H nuclear magnetic resonance shift of UV-P.

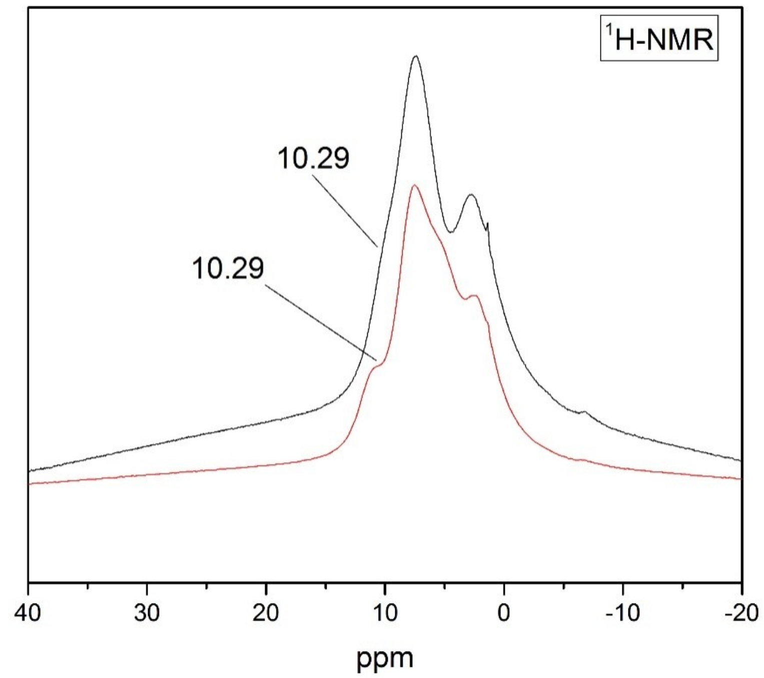

Figure S6. ^{1}H solid-state nuclear magnetic resonance spectra of **A.B.** (red line) and **A.C.** (black line) samples.

^{1}H-NMR positions of the UV-P molecule can be theoretically predicted using the ChemDraw software, and the signals for H atom attached to O and N atoms appear at 10.29 and 16.16 ppm respectively (Figure S5). For the experimental solid-state NMR results, although the resolution of NMR is relative lower, it was observed that should peaks located at ca. 10.29 ppm appeared for **A.B** and **A.C** as shown in Figure S6, while there is a lack of signal at ca. 16.16 ppm. Therefore, it can be concluded that the H-atom in **A.B.** and **A.C.** belongs to hydroxyl group.

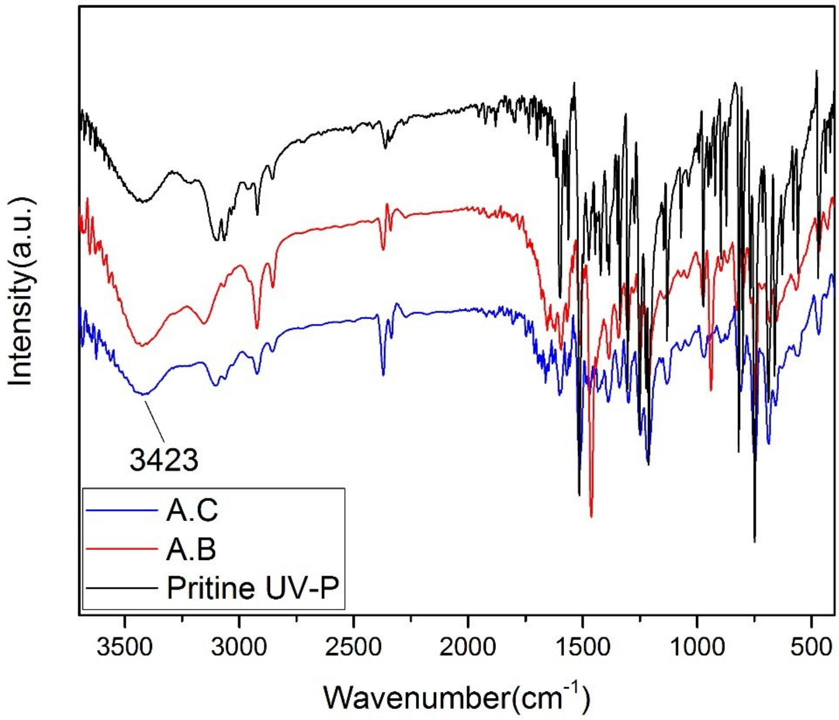

Figure S7. Fourier transform infrared spectra of pristine UV-P (black line), **A.B.** (blue line) and **A.C.** (red line) samples.

FTIR spectra of **A.B.** and **A.C.** (Figure S7) show that broad OH peak appeared at 3423 cm^{-1}, which has no obvious sift relative to the pristine UV-P, suggesting that the position of H-atom in OH in the ground state for **A.B.** and **A.C**..

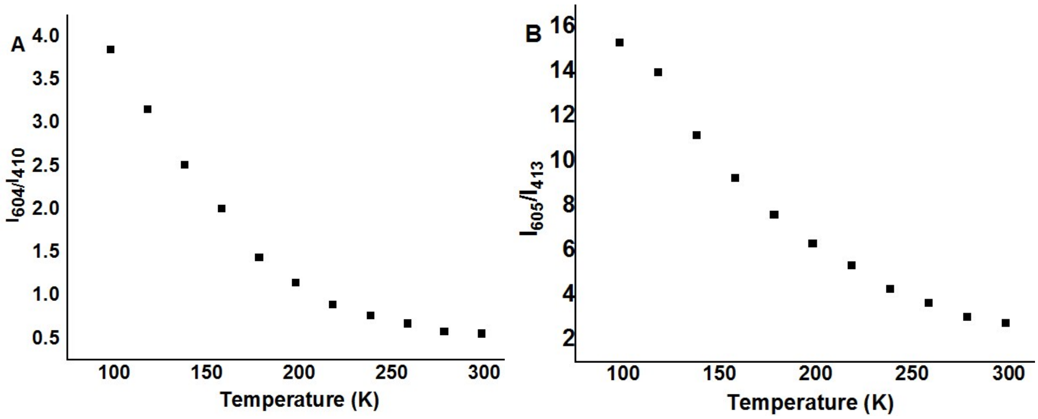

Figure S8. Temperature-dependent values of I_{604}/I_{410} for **A** (A) and I_{605}/I_{413} for **A.B.** (B).

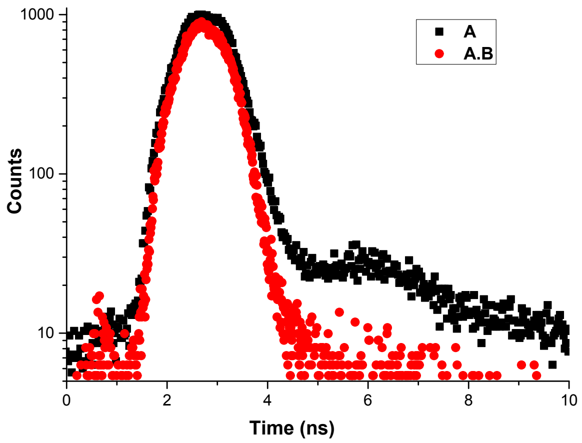

Figure S9. Fluorescence decay curves of solid-state **A** (crystal, red line) and **A.B.** (crystal, black line) samples monitored at 605 nm.

Table S2: Comparison of main parameters of the UV-P molecule in pristine **A** and cocrystals.

Samples	Pristine UV-P	**A.B**	**A.C**
Distance a	1.430	1.432	1.437
Distance b	1.361	1.362	1.365
Angle A	124.57°	125.77°	125.37°
dihedral angle B	179.31°	177.4°	168.09°

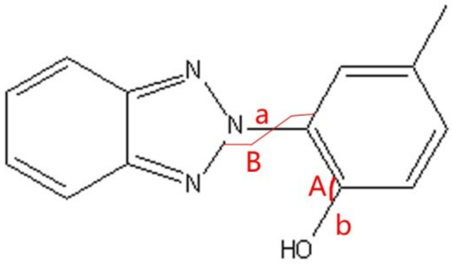

The possible conformational changes of UV-P have been understood by comprising the typical conformational parameters (such as the N-C and C-O distances, ∠CCO (angle A), and ∠NNCC (dihedral angle B) as shown above) of UV-P in the pristine crystal and cocrystals **A.B** and **A.C**. It was observed that the parameters are very close for the pristine **A** and **A.B.**, and thus restrict the understanding on the alternation of optical properties from a molecular structure view. However, for **A.C.**, the dihedral angle between the phenyl and benzotriazole groups has decreased relative to that of the pristine

A, indicating that the coplanarity of **A** has been reduced after intermolecular interaction with **C**. The change of coplanarity could explain the largely decreased ESIPT emission and quantum yield in experiment from a conformation perspective. Therefore, the conformational factors can partially influence the ESIPT emission of the samples.

Computational Parts

Ground State Periodic Calculations. Ground-state bulk molecular crystals are first optimized using the DMol3 module of Materials Studio package. [1-4] The starting geometric parameters of molecular crystals are taken from the experimental data. Full optimizations of the enol forms of molecular crystals **A**, **A.B.**, and **A.C.** including cell parameters and molecular geometric parameters are carried out at the PBE level [5-6] with periodic boundary conditions (PBC). The DNP basis set [7] is used to expand the electronic wavefunction and the default dispersion correction is added to consider intermolecular weak interactions. A Monkhorst-Pack k-point grids of 2*2*2 are used. [8] Default convergence criteria on gradients and displacements are used.

QM/QM′ Calculations. For **A** molecular crystal, a cluster of 15 molecules of **UVP** was extracted from the fully optimized molecular crystals in the enol form for the ONIOM QM/QM′ calculations. A **UVP** molecule is treated using a high-level QM method; while the remaining 14 molecules are done using a low-level QM′ method. For cocrystal **A.B.**, a **UVP** molecule is treated at the high QM level; 12 **UVP** molecules and 5 **B** cocrystal molecules are kept in the low-level QM′ region. For cocrystal **A.C.**, a **UVP** molecule is included in the high-level QM region; 8 **UVP** molecules and 7 cocrystal **C** molecules are placed in the low-level QM′ region.

For the S_0 calculations, the QM region is fully optimized at the CAM-B3LYP/6-31+G* level [9-14] with surrounding molecules fixed (QM′ region; HF/3-21G level). [15-19] For the S_1 calculations, the QM region is described using the TD-CAM-B3LYP/6-31+G* method; [20, 21] the QM′ region still uses the same HF/3-21G method. In the ONIOM QM/QM′ calculations, the Mülliken charge embedding scheme is also used to consider the polarization effects. This computational protocol for molecular crystals has recently been used by Adamo et al. [22-24] to compute excited-state properties of molecular crystals. In all DFT and TD-DFT computations, the LANL2DZ pseudopotential and basis set is applied for the iodine atom; [25] All ONIOM QM/QM′ calculations are carried out using the GAUSSIAN09 package. [26]

QM/MM Calculations. Vertical excitation and emission energies are also computed using the QM/MM method in which the QM region (the QM layer in

the QM/QM′ calculations) is treated by the MS-CASPT2 method with the MM subsystem (the QM′ layer) is modeled using the Mülliken charges same as those used in the QM/QM′ calculations (see above).

In single-point MS-CASPT2 calculations, [27, 28] an active space of ten electrons in eight orbitals is adopted. The Cholesky decomposition technique with unbiased auxiliary basis sets is used for accurate two-electron integral approximations. [29] The imaginary shift technique (0.2 au) is employed to avoid intruder-state issues. [30] The ionization potential−electron affinity shift is not used in all MS-CASPT2 computations. [31] In all computations, the LANL2DZ pseudopotential and basis set [32] is applied for the iodine atom; all other atoms (C, H, O, N, and F) are treated with 6-31G* basis set. [33, 34] All MS-CASPT2 computations are performed using MOLCAS8.0. [35, 36]

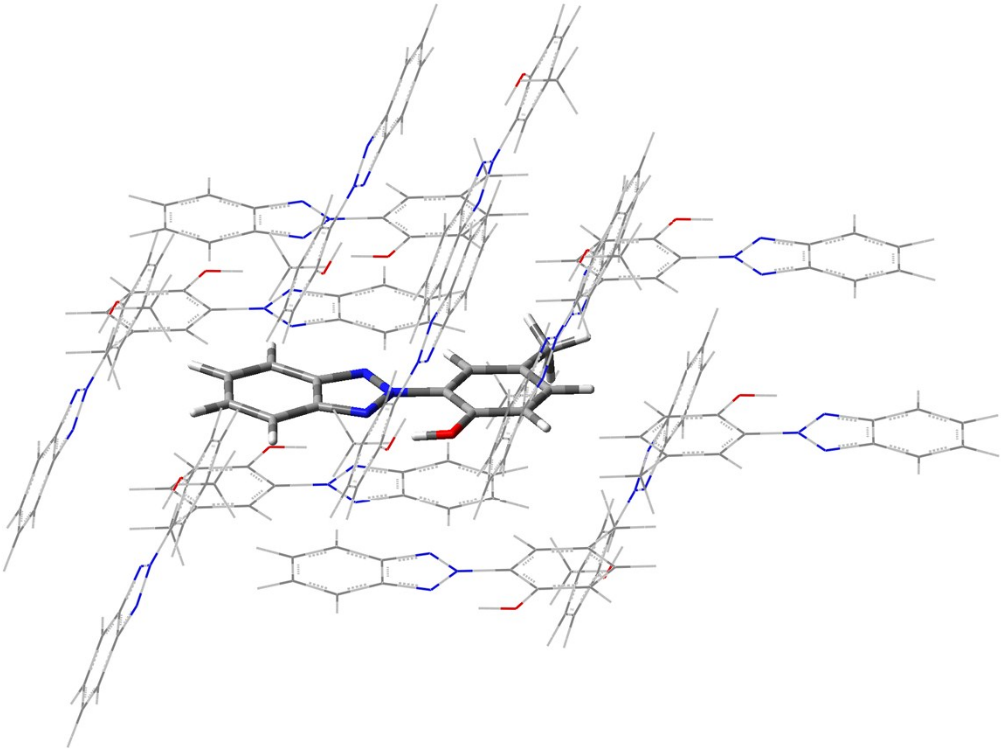

A

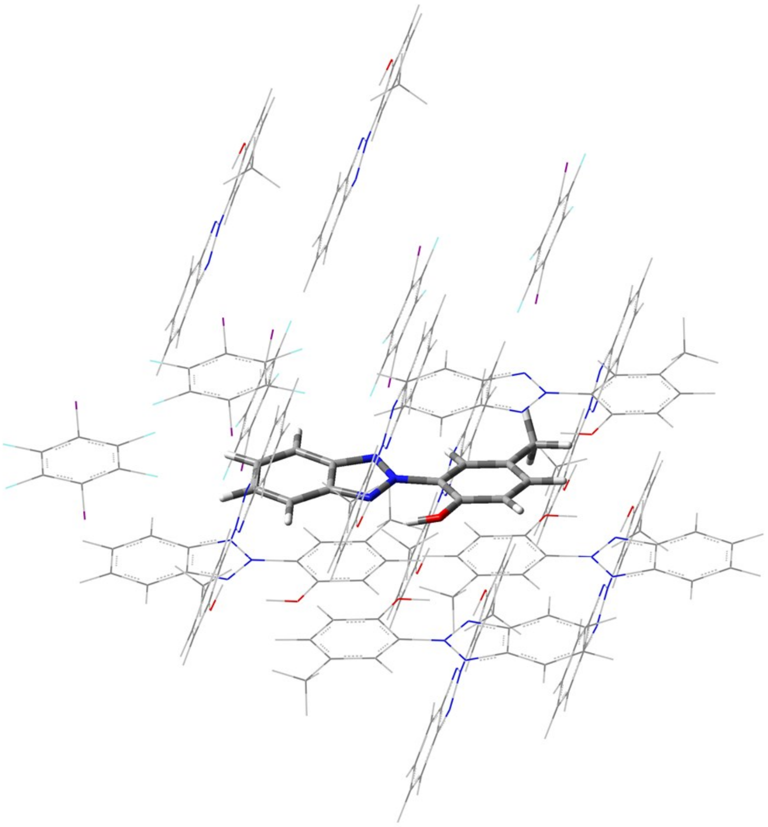

A.B.

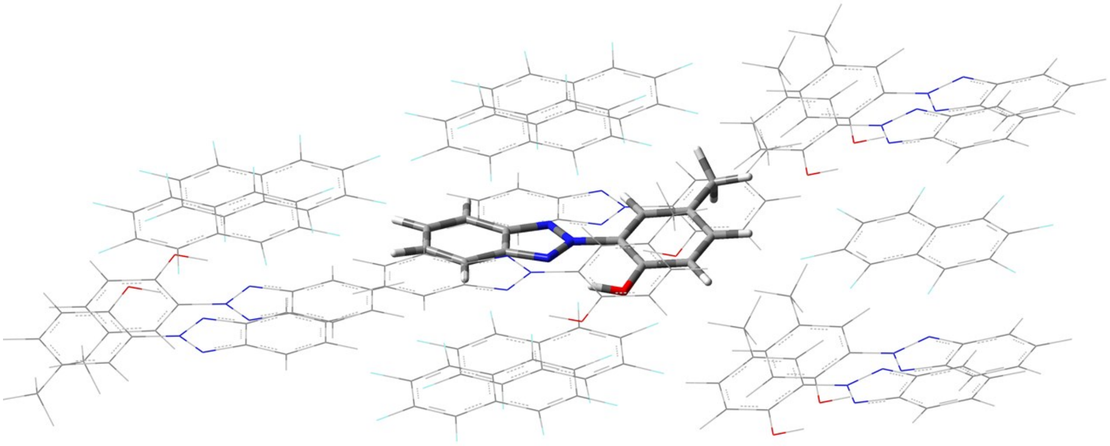

A.C.

Figure S10. Schematic representations of the **A**, **A.B.**, and **A.C.** clusters in the enol form.

Table S3: Vertical Emission Bands (in kcal/mol and nm) of Molecular Crystals **A**, **A.B.**, and **A.C.** Computed by ONIOM(TD-CAM-B3LYP/6-31+G*:HF/3-21G) Method.

	**enol**		**keto**	
	kcal/mol (nm)	Exp.	kcal/mol (nm)	Exp.
**UV-P (A.)**	76.6 (373)	69.7 (410)	44.6 (641)	47.3 (604)

**UV-I (A.B.)**	78.1 (366)	69.2 (413)	48.0 (596)	47.3 (605)
**UV-F (A.C.)**	77.6 (368)	69.7 (410)	42.6 (672)	46.4 (617)

Table S4: Vertical Emission Bands (in kcal/mol and nm) of Molecular Cocrystals **A**, **A.B.**, and **A.C.** Computed by MS-CASPT2(10,8)//ONIOM(TD-CAM-B3LYP/6-31+G*:HF/3-21G) Method. Also Shown are the Oscillator Strengths (f) in Square Brackets.

	**enol**		**keto**	
	kcal/mol (nm)	Exp.	kcal/mol (nm)	Exp.
**UV-P (A.)**	71.9 (398) [0.5207]	69.7 (410)	51.3 (557) [0.4991]	47.3 (604)
**UV-I (A.B.)**	71.8 (398) [0.7822]	69.2 (413)	50.1 (571) [0.4270]	47.3 (605)
**UV-F (A.C.)**	65.2 (438) [0.6894]	69.7 (410)	47.3 (604) [0.2313]	46.4 (617)

Table S5: MS-CASPT2(10,8)//ONIOM Computed Relative Energies (in kcal/mol) of S_1 Enol and Keto Species of Molecular Crystals **A**, **A.B.**, and **A.C.**

	**A**	**A.B.**	**A.C.**
S_1 (enol)	79.5	79.1	70.2
S_1 (keto)	73.1	73.9	68.9

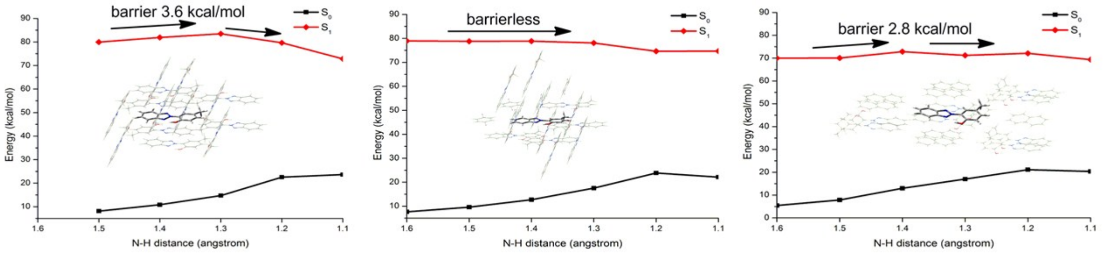

A. **A.B.** **A.C.**

Figure S11. Minimum-energy S_1 intramolecular excited-state proton transfer paths of molecular cocrystals **A**, **A.B.**, and **A.C.** computed by MS-CASPT2(10,8)//ONIOM method.

MS-CASPT2 (10e,8o) for S_0 and S_1 (enol) states

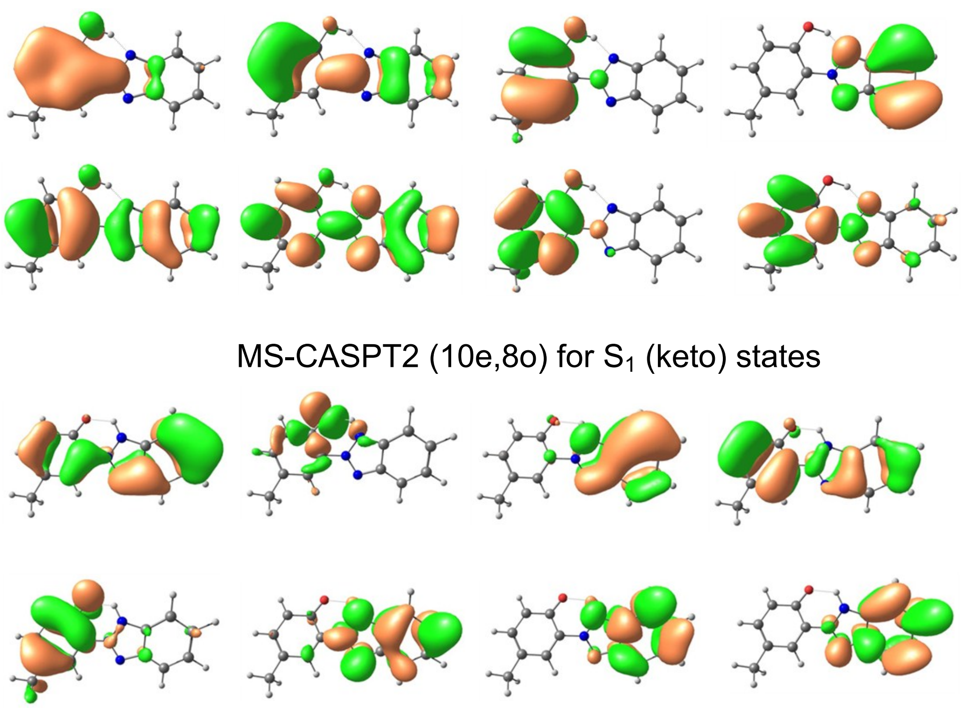

Figure S12. Active spaces used for molecular cocrystals **A**, **A.B.**, and **A.C.** in the MS-CASPT2(10,8)//ONIOM computations.

1. References

[1] B. Delley, An all-electron numerical method for solving the local density functional for polyatomic molecules. *J. Chem. Phys.,* 92: 508, 1990.

[2] B. Delley, Fast Calculation of Electrostatics in Crystals and Large Molecules. *J. Phys. Chem.,* 100: 6107, 1996.

[3] B. Delley, From molecules to solids with the DMol3 approach. *J. Chem. Phys.,* 113: 7756, 2000.

[4] B. Delley, D. E. Ellis, A. J. Freeman, E. J. Baerends, and D. Post, Binding energy and electronic structure of small copper particles. *Phys. Rev. B* 27: 2132, 1983.

[5] J. P. Perdew, K. Burke, and M. Ernzerhof, Generalized gradient approximation made simple, *Phys. Rev. Lett.*, 77: 3865, 1996.

[6] J. P. Perdew, K. Burke, and M. Ernzerhof, Errata: Generalized gradient approximation made simple, *Phys. Rev. Lett.*, 78: 1396, 1997.

[7] B. Delley, D. E. Ellis, A. J. Freeman, E. J. Baerends, and D. Post, Binding energy and electronic structure of small copper particles. *Phys. Rev. B* 27: 2132, 1983.

[8] H. J. Monkhorst and J. D. Pack, Special points for Brillouin-zone integrations. *Phys. Rev. B* 13: 5188,1976.

[9] S. H. Vosko, L. Wilk, M. Nusair, Accurate spin-dependent electron liquid correlation energies for local spin density calculations: a critical analysis. *Can.*

J. Phys., 58: 1200, 1980.
[10] A. D. Becke, Density-Functional Exchange-Energy Approximation with Correct Asymptotic Behavior. *Phys. Rev. A: At., Mol., Opt. Phys.,* 38: 3098, 1988.
[11] C. Lee, W. T. Yang, R. G. Parr, Phys. Development of the Colle Salvetti Correlation-Energy Formula into a Functional of the Electron Density. *Phys. Rev. B: Condens. Matter Mater. Phys.,* 37: 785, 1988.
[12] A. D. Becke, A New Mixing of Hartree-Fock and Local Density-Functional Theories. *J. Chem. Phys.,* 98: 1372, 1993.
[13] P. C. Hariharan, J. A. Pople, Influence of polarization functions on molecular-orbital hydrogenation energies, *Theor. Chim. Acta* 28: 213, 1973.
[14] M. M. Francl, W. J. Pietro, W. J. Hehre, J. S. Binkley, M. S. Gordon, D. J. De-Frees, J. A. Pople, Self-Consistent Molecular Orbital Methods. 23. A polarization-type basis set for 2nd-row elements, *J. Chem. Phys.,* 77: 3654, 1982.
[15] C. C. J. Roothaan, New Developments in Molecular Orbital Theory, *Rev. Mod. Phys.,* 23: 69, 1951.
[16] J. A. Pople and R. K. Nesbet, Self-Consistent Orbitals for Radicals, *J. Chem. Phys.*, 22: 571, 1954.
[17] R. McWeeny and G. Dierksen, Self-consistent perturbation theory. 2. Extension to open shells, *J. Chem. Phys.*, 49: 4852, 1968.
[18] J. S. Binkley, J. A. Pople, and W. J. Hehre, Self-Consistent Molecular Orbital Methods. 21. Small Split-Valence Basis Sets for First-Row Elements, *J. Am. Chem. Soc.*, 102: 939, 1980.
[19] M. S. Gordon, J. S. Binkley, J. A. Pople, W. J. Pietro, and W. J. Hehre, Self-Consistent Molecular Orbital Methods. 22. Small Split-Valence Basis Sets for Second-Row Elements, *J. Am. Chem. Soc.*, 104:2797, 1982.
[20] T. Yanai, D. Tew, and N. Handy. A new hybrid exchange-correlation functional using the Coulomb-attenuating method (CAM-B3LYP). *Chem. Phys. Lett.,* 393: 51, 2004.
[21] M. A. L. Marques, C. A. Ullrich, F. Nogueira, A. Rubio, K. Burke, E. K. U. Gross, Eds. Time-Dependent Density Functional Theory; Springer: Berlin, Heidelberg, New York, 2006.
[22] H. P. Hratchian, P. V. Parandekar, K. Raghavachari, M. J. Frisch, T. Vreven, QM:QM Electronic Embedding Using Mulliken Atomic Charges: Energies and Analytic Gradients in an ONIOM Framework. *J. Chem. Phys.,* 128: 034107, 2008.
[23] D. Presti, F. Labat, A. Pedone, M. J. Frisch, H. P. Hratchian, I. Ciofini, M. C. Menziani, and C. Adamo, Computational Protocol for Modeling Thermochromic Molecular Crystals: Salicylidene Aniline As a Case Study, *J. Chem. Theory Comput.*, 10: 5577, 2014.
[24] G. García, I. Ciofini, M. Fernández-Gómez, C. Adamo, Confinement Effects on UV−Visible Absorption Spectra: Β-Carotene Inside Carbon Nanotube as a Test Case. *J. Phys. Chem. Lett.,* 4: 1239, 2013.

[25] P. J. Hay, and W. R. Wadt. Ab initio effective core potentials for molecular calculations. Potentials for K to Au including the outermost core orbitals. *J. Chem. Phys.,* 82: 299, 1985.
[26] Gaussian 09, Revision B.01, M. J. Frisch, G. W. Trucks, H. B. Schlegel, G. E. Scuseria, M. A. Robb, J. R. Cheeseman, G. Scalmani, V. Barone, B. Mennucci, G. A. Petersson, H. Nakatsuji, M. Caricato, X. Li, H. P. Hratchian, A. F. Izmaylov, J. Bloino, G. Zheng, J. L. Sonnenberg, M. Hada, M. Ehara, K. Toyota, R. Fukuda, J. Hasegawa, M. Ishida, T. Nakajima, Y. Honda, O. Kitao, H. Nakai, T. Vreven, J. A. Montgomery, Jr., J. E. Peralta, F. Ogliaro, M. Bearpark, J. J. Heyd, E. Brothers, K. N. Kudin, V. N. Staroverov, R. Kobayashi, J. Normand, K. Raghavachari, A. Rendell, J. C. Burant, S. S. Iyengar, J. Tomasi, M. Cossi, N. Rega, J. M. Millam, M. Klene, J. E. Knox, J. B. Cross, V. Bakken, C. Adamo, J. Jaramillo, R. Gomperts, R. E. Stratmann, O. Yazyev, A. J. Austin, R. Cammi, C. Pomelli, J. W. Ochterski, R. L. Martin, K. Morokuma, V. G. Zakrzewski, G. A. Voth, P. Salvador, J. J. Dannenberg, S. Dapprich, A. D. Daniels, □. Farkas, J. B. Foresman, J. V. Ortiz, J. Cioslowski, D. J. Fox, Gaussian, Inc., Wallingford CT, 2010.
[27] K. Andersson, P. Malmqvist, B. Roos, A. Sadlej, and K. Wolinski. Second-Order Perturbation Theory with a CASSCF Reference Function. *J. Phys. Chem.,* 94: 5483, 1990.
[28] K. Andersson, P. Malmqvist, and B. Roos. Second-order perturbation theory with a complete active space self-consistent field reference function. *J. Chem. Phys.,* 96: 1218, 1992.
[29] F. Aquilante, R. Lindh, T. B. Pedersen, Unbiased Auxiliary Basis Sets for Accurate Two-Electron Integral Approximations. *J. Chem. Phys.,* 127: 114107, 2007.
[30] N. Försberg, P. Å. Malmqvist, Multiconfiguration Perturbation Theory with Imaginary Level Shift. *Chem. Phys. Lett.,* 274: 196, 1997.
[31] G. Ghigo, B. O. Roos, P. Malmqvist, A Modified Definition of the Zeroth-Order Hamiltonian in Multiconfigurational Perturbation Theory (CASPT2). *Chem. Phys. Lett.,* 396: 142, 2004.
[32] W.R. Wadt and P.J. Hay, Ab initio effective core potentials for molecular calculations. potentials for main group elements sodium to bismuth, *J. Chem. Phys.,* 82: 284, 1985.
[33] R. Ditchfield, W. J. Hehre, J. A. Pople, Self-Consistent Molecular-Orbital Methods. IX. An Extended Gaussian-Type Basis for Molecular-Orbital Studies of Organic Molecules. *J. Chem. Phys.,* 54: 724, 1971.
[34] M. M. Francl, W. J. Pietro, W. J. Hehre, J. S. Binkley, M. S. Gordon, D. J. DeFrees, J. A. Pople, Self-Consistent Molecular Orbital Methods. XXIII. A Polarization-Type Basis Set for Second-Row Elements. *J. Chem. Phys.,* 77: 3654, 1982.
[35] G. Karlström, R. Lindh, P. Å. Malmqvist, B. O. Roos, U. Ryde, V. Veryazov, P. O. Widmark, M. Cossi, B. Schimmelpfennig, P. Neogrady, et al. MOLCAS: A Program Package for Computational Chemistry. *Comput. Mater.*

Sci., 28: 222, 2003.
[36] F. Aquilante, L. De Vico, N. Ferré, G. Ghigo, P. Malmqvist, P. Neogrády, T. B.; Pitoňák, M. Pedersen, M. Reiher, B. O. Roos, et al. MOLCAS 7: the Next Generation. *J. Comput. Chem.,* 31: 224, 2010.

4. Cartesian Coordinates of All Optimized Structures

In xyz format (unit: angström)

UVP (A)

S_0

O	0.211654000	-0.844954000	-1.972261000
N	-2.273811000	1.661522000	0.336328000
N	-2.090271000	-0.197761000	-0.912834000
N	-1.506462000	0.884624000	-0.400018000
C	0.659833000	0.307613000	-1.414558000
C	1.994300000	0.649342000	-1.619417000
C	2.524225000	1.810434000	-1.082053000
C	1.741264000	2.676229000	-0.313906000
C	0.418558000	2.325517000	-0.086714000
C	-0.129355000	1.168018000	-0.636397000
C	-3.355136000	-0.129952000	-0.461399000
C	-3.465845000	1.036878000	0.329368000
C	-4.669988000	1.370399000	0.990211000
C	-5.713143000	0.491820000	0.843837000
C	-4.457658000	-0.993347000	-0.647715000
C	-5.608864000	-0.673738000	0.025248000
C	2.299528000	3.977370000	0.195911000
H	-0.763907000	-0.888988000	-1.840586000
H	2.593297000	-0.016257000	-2.229895000
H	3.566159000	2.058090000	-1.254518000
H	-0.216037000	2.956095000	0.519764000
H	-4.737371000	2.278050000	1.579418000
H	-6.648976000	0.671094000	1.360875000
H	-4.409242000	-1.858565000	-1.295872000
H	-6.460920000	-1.331375000	-0.091372000
H	2.076419000	4.787641000	-0.504389000
H	3.383930000	3.923023000	0.310915000
H	1.860053000	4.249321000	1.158238000
O	4.087389000	-2.032129000	6.980103000
N	2.686201000	-4.231366000	3.649166000
N	2.634303000	-2.292878000	4.843298000
N	3.118517000	-3.545082000	4.704612000
C	4.497924000	-3.303355000	6.733580000
C	5.415952000	-3.886324000	7.617967000
C	5.879568000	-5.184091000	7.418645000

C	5.448330000	-5.954480000	6.323864000
C	4.536235000	-5.380503000	5.437260000
C	4.057533000	-4.077667000	5.629322000
C	1.816854000	-2.144810000	3.772175000
C	1.857584000	-3.358778000	3.021811000
C	1.135625000	-3.507457000	1.816429000
C	0.398694000	-2.410801000	1.396033000
C	1.038658000	-1.046294000	3.342959000
C	0.348395000	-1.201432000	2.148469000
C	5.917857000	-7.372570000	6.131842000
H	3.407798000	-1.806062000	6.272289000
H	5.739865000	-3.292455000	8.474926000
H	6.593268000	-5.622846000	8.119767000
H	4.174680000	-5.943091000	4.577745000
H	1.173955000	-4.444876000	1.256691000
H	-0.172799000	-2.468859000	0.468387000
H	0.973926000	-0.125662000	3.924494000
H	-0.255501000	-0.369931000	1.780837000
H	5.070406000	-8.068039000	6.248443000
H	6.688908000	-7.642160000	6.868107000
H	6.328960000	-7.524614000	5.119951000
O	-8.098221000	-0.618703000	3.297982000
N	-9.499409000	-2.817939000	-0.032955000
N	-9.551306000	-0.879451000	1.161177000
N	-9.067093000	-2.131655000	1.022491000
C	-7.687686000	-1.889927000	3.051459000
C	-6.769657000	-2.472896000	3.935846000
C	-6.306042000	-3.770664000	3.736524000
C	-6.737279000	-4.541052000	2.641743000
C	-7.649374000	-3.967076000	1.755139000
C	-8.128076000	-2.664240000	1.947201000
C	-10.368756000	-0.731382000	0.090054000
C	-10.328024000	-1.945352000	-0.660310000
C	-11.049984000	-2.094030000	-1.865692000
C	-11.786915000	-0.997374000	-2.286088000
C	-11.146951000	0.367133000	-0.339162000
C	-11.837214000	0.211995000	-1.533652000
C	-6.267753000	-5.959143000	2.449721000
H	-8.777811000	-0.392635000	2.590168000
H	-6.445744000	-1.879028000	4.792805000
H	-5.592342000	-4.209418000	4.437646000
H	-8.010929000	-4.529664000	0.895624000
H	-11.011654000	-3.031450000	-2.425430000
H	-12.358409000	-1.055431000	-3.213734000

H	-11.211684000	1.287766000	0.242373000
H	-12.441111000	1.043495000	-1.901284000
H	-7.115203000	-6.654613000	2.566322000
H	-5.496701000	-6.228733000	3.185986000
H	-5.856650000	-6.111187000	1.437830000
O	-1.383342000	-6.571523000	2.367500000
N	0.017847000	-4.372286000	5.698437000
N	0.069960000	-6.309871000	4.503932000
N	-0.414471000	-5.058569000	4.642990000
C	-1.793877000	-5.300298000	2.614023000
C	-2.711470000	-4.716843000	1.728381000
C	-3.175521000	-3.419561000	1.928958000
C	-2.744066000	-2.649589000	3.022857000
C	-1.831971000	-3.222246000	3.909971000
C	-1.353269000	-4.525082000	3.717908000
C	0.887192000	-6.458842000	5.575428000
C	0.846462000	-5.244873000	6.325792000
C	1.568639000	-5.095292000	7.530802000
C	2.305570000	-6.191948000	7.951198000
C	1.665388000	-7.557358000	6.004644000
C	2.355869000	-7.401318000	7.198761000
C	-3.213593000	-1.230179000	3.215388000
H	-0.703316000	-6.797104000	3.074059000
H	-3.035601000	-5.310294000	0.872304000
H	-3.889003000	-2.981223000	1.226954000
H	-1.470634000	-2.660562000	4.769858000
H	1.530308000	-4.157873000	8.090540000
H	2.876845000	-6.134793000	8.879216000
H	1.730338000	-8.477088000	5.422736000
H	2.959549000	-8.233720000	7.566766000
H	-2.366142000	-0.536030000	3.098278000
H	-3.984643000	-0.961908000	2.478613000
H	-3.624696000	-1.078135000	4.227279000
O	5.368735000	-4.478783000	1.800437000
N	3.967547000	-6.678019000	-1.530500000
N	3.915650000	-4.739532000	-0.336367000
N	4.399864000	-5.991736000	-0.475053000
C	5.779271000	-5.750007000	1.553914000
C	6.697298000	-6.332977000	2.438301000
C	7.160914000	-7.630744000	2.238979000
C	6.729677000	-8.401132000	1.144198000
C	5.817582000	-7.827157000	0.257594000
C	5.338879000	-6.524321000	0.449656000
C	3.098201000	-4.591462000	-1.407491000

C	3.138931000	-5.805432000	-2.157855000
C	2.416972000	-5.954111000	-3.363237000
C	1.680041000	-4.857454000	-3.783633000
C	2.320005000	-3.492947000	-1.836706000
C	1.629742000	-3.648084000	-3.031196000
C	7.199204000	-9.819224000	0.952176000
H	4.689145000	-4.252716000	1.092623000
H	7.021211000	-5.739109000	3.295260000
H	7.874614000	-8.069499000	2.940101000
H	5.456027000	-8.389744000	-0.601921000
H	2.455302000	-6.891530000	-3.922975000
H	1.108548000	-4.915512000	-4.711279000
H	2.255273000	-2.572315000	-1.255171000
H	1.025844000	-2.816587000	-3.398830000
H	6.351753000	-10.514693000	1.068777000
H	7.970255000	-10.088813000	1.688441000
H	7.610307000	-9.971267000	-0.059715000
O	-6.816875000	-3.065356000	-1.881684000
N	-8.218063000	-5.264593000	-5.212621000
N	-8.269959000	-3.326105000	-4.018488000
N	-7.785746000	-4.578309000	-4.157174000
C	-6.406339000	-4.336580000	-2.128207000
C	-5.488310000	-4.919550000	-1.243820000
C	-5.024695000	-6.217317000	-1.443142000
C	-5.455932000	-6.987706000	-2.537923000
C	-6.368027000	-6.413730000	-3.424527000
C	-6.846730000	-5.110894000	-3.232465000
C	-9.087409000	-3.178036000	-5.089612000
C	-9.046678000	-4.392005000	-5.839976000
C	-9.768637000	-4.540684000	-7.045358000
C	-10.505568000	-3.444028000	-7.465754000
C	-9.865604000	-2.079520000	-5.518827000
C	-10.555868000	-2.234658000	-6.713318000
C	-4.986406000	-8.405797000	-2.729945000
H	-7.496464000	-2.839288000	-2.589498000
H	-5.164398000	-4.325682000	-0.386861000
H	-4.310995000	-6.656072000	-0.742020000
H	-6.729582000	-6.976317000	-4.284042000
H	-9.730307000	-5.478103000	-7.605096000
H	-11.077062000	-3.502085000	-8.393400000
H	-9.930337000	-1.158888000	-4.937292000
H	-11.159764000	-1.403158000	-7.080950000
H	-5.833857000	-9.101265000	-2.613344000
H	-4.215354000	-8.675387000	-1.993680000

H	-4.575303000	-8.557841000	-3.741836000
O	1.476263000	-3.198003000	-7.102043000
N	-1.019726000	-0.546336000	-4.947766000
N	-0.815604000	-2.502480000	-6.095901000
N	-0.236146000	-1.373654000	-5.635806000
C	1.940586000	-2.028791000	-6.589778000
C	3.291146000	-1.708421000	-6.790053000
C	3.829877000	-0.527454000	-6.286466000
C	3.043532000	0.383772000	-5.560549000
C	1.698891000	0.072877000	-5.355352000
C	1.142464000	-1.109892000	-5.860199000
C	-2.095271000	-2.408229000	-5.658024000
C	-2.217387000	-1.184284000	-4.933638000
C	-3.430806000	-0.810726000	-4.312121000
C	-4.488227000	-1.699252000	-4.433923000
C	-3.191128000	-3.288442000	-5.795529000
C	-4.371329000	-2.916347000	-5.166236000
C	3.616964000	1.681817000	-5.053815000
H	0.490893000	-3.230949000	-6.897329000
H	3.897708000	-2.410218000	-7.365851000
H	4.884325000	-0.293254000	-6.454693000
H	1.053859000	0.752220000	-4.799997000
H	-3.508877000	0.130911000	-3.765340000
H	-5.446595000	-1.463744000	-3.968555000
H	-3.118784000	-4.209958000	-6.374764000
H	-5.236403000	-3.577458000	-5.242482000
H	3.139841000	2.529997000	-5.570224000
H	4.701154000	1.736214000	-5.229483000
H	3.428272000	1.808453000	-3.973770000
O	-0.101995000	-9.018175000	-2.812166000
N	1.299193000	-6.818939000	0.518771000
N	1.351306000	-8.756525000	-0.675734000
N	0.866876000	-7.505223000	-0.536675000
C	-0.512531000	-7.746951000	-2.565643000
C	-1.430123000	-7.163496000	-3.451284000
C	-1.894174000	-5.866214000	-3.250708000
C	-1.462719000	-5.096243000	-2.156811000
C	-0.550626000	-5.668900000	-1.269695000
C	-0.071922000	-6.971735000	-1.461757000
C	2.168539000	-8.905496000	0.395762000
C	2.127809000	-7.691526000	1.146127000
C	2.849986000	-7.541946000	2.351136000
C	3.586917000	-8.638602000	2.771532000
C	2.946735000	-10.004011000	0.824978000

C	3.637216000	-9.847971000	2.019095000
C	-1.932248000	-3.676835000	-1.964303000
H	0.578031000	-9.243758000	-2.105607000
H	-1.754254000	-7.756948000	-4.307361000
H	-2.607656000	-5.427877000	-3.952712000
H	-0.189287000	-5.107214000	-0.409808000
H	2.811655000	-6.604526000	2.910874000
H	4.158192000	-8.581447000	3.699550000
H	3.011685000	-10.923741000	0.243070000
H	4.240894000	-10.680373000	2.387100000
H	-1.084802000	-2.982704000	-2.081353000
H	-2.703299000	-3.408559000	-2.701049000
H	-2.343345000	-3.524794000	-0.952384000
O	-1.086430000	1.695304000	3.257289000
N	-3.582420000	4.346971000	5.411565000
N	-3.378298000	2.390827000	4.263431000
N	-2.798839000	3.519654000	4.723526000
C	-0.622107000	2.864517000	3.769554000
C	0.728453000	3.184886000	3.569279000
C	1.267183000	4.365853000	4.072866000
C	0.480839000	5.277079000	4.798783000
C	-0.863803000	4.966184000	5.003979000
C	-1.420229000	3.783415000	4.499132000
C	-4.657964000	2.485078000	4.701307000
C	-4.780081000	3.709023000	5.425693000
C	-5.993499000	4.082581000	6.047211000
C	-7.050920000	3.194054000	5.925408000
C	-5.753822000	1.604864000	4.563803000
C	-6.934022000	1.976959000	5.193096000
C	1.054270000	6.575123000	5.305516000
H	-2.071801000	1.662358000	3.462002000
H	1.335015000	2.483089000	2.993481000
H	2.321631000	4.600053000	3.904638000
H	-1.508835000	5.645526000	5.559334000
H	-6.071571000	5.024218000	6.593992000
H	-8.009288000	3.429563000	6.390776000
H	-5.681478000	0.683350000	3.984568000
H	-7.799097000	1.315849000	5.116849000
H	0.577147000	7.423304000	4.789107000
H	2.138461000	6.629520000	5.129849000
H	0.865578000	6.701761000	6.385561000
O	6.377081000	3.705915000	2.906148000
N	8.872853000	1.053345000	0.752244000
N	8.668513000	3.009906000	1.901261000

N	8.089054000	1.881080000	1.441166000
C	5.912541000	2.535800000	2.394256000
C	4.561981000	2.215431000	2.594530000
C	4.023251000	1.034463000	2.090943000
C	4.809595000	0.123237000	1.365027000
C	6.154018000	0.434549000	1.160712000
C	6.710444000	1.617318000	1.665559000
C	9.948179000	2.915655000	1.463384000
C	10.070731000	1.692195000	0.737744000
C	11.283714000	1.318152000	0.117481000
C	12.341135000	2.206678000	0.239283000
C	11.044472000	3.796354000	1.599634000
C	12.224456000	3.423357000	0.970714000
C	4.236163000	-1.174806000	0.858293000
H	7.362235000	3.737958000	2.701807000
H	3.955418000	2.917227000	3.170328000
H	2.968803000	0.800263000	2.259171000
H	6.799051000	-0.244794000	0.605357000
H	11.362004000	0.376098000	-0.430183000
H	13.299503000	1.971170000	-0.226085000
H	10.971693000	4.717384000	2.180124000
H	13.089313000	4.084884000	1.047842000
H	4.713287000	-2.022988000	1.374702000
H	3.152190000	-1.228302000	1.033588000
H	4.424855000	-1.301444000	-0.221752000
O	2.484392000	4.985792000	-5.995959000
N	3.885362000	7.185445000	-2.664140000
N	3.937476000	5.246542000	-3.859155000
N	3.453264000	6.498745000	-3.720469000
C	2.073638000	6.257434000	-5.748554000
C	1.155828000	6.839987000	-6.633823000
C	0.691994000	8.138171000	-6.433619000
C	1.123450000	8.908142000	-5.339720000
C	2.035762000	8.335068000	-4.453489000
C	2.514247000	7.031330000	-4.645179000
C	4.754709000	5.098889000	-2.787149000
C	4.714195000	6.312441000	-2.037667000
C	5.435937000	6.461537000	-0.831403000
C	6.173086000	5.364464000	-0.411889000
C	5.532903000	4.000374000	-2.357934000
C	6.223167000	4.155511000	-1.163444000
C	0.653924000	10.326234000	-5.147699000
H	3.163981000	4.759725000	-5.288146000
H	0.831915000	6.246118000	-7.490783000

H	-0.021488000	8.576508000	-7.135623000
H	2.397100000	8.896753000	-3.593601000
H	5.397825000	7.398539000	-0.272547000
H	6.744579000	5.422522000	0.515756000
H	5.597855000	3.079325000	-2.940351000
H	6.827282000	3.323595000	-0.796694000
H	1.501374000	11.021702000	-5.264299000
H	-0.117128000	10.595823000	-5.883964000
H	0.242603000	10.478693000	-4.134925000
O	-5.808528000	5.119342000	-0.775973000
N	-3.312756000	2.466772000	-2.929877000
N	-3.517096000	4.423333000	-1.780860000
N	-4.096556000	3.294506000	-2.240955000
C	-6.273068000	3.949227000	-1.287866000
C	-7.623629000	3.628858000	-1.087591000
C	-8.162359000	2.447891000	-1.591178000
C	-7.376015000	1.536664000	-2.317095000
C	-6.031591000	1.847976000	-2.521409000
C	-5.475165000	3.030746000	-2.016562000
C	-2.237430000	4.329082000	-2.218737000
C	-2.114878000	3.105623000	-2.944377000
C	-0.901895000	2.731579000	-3.564640000
C	0.155526000	3.620106000	-3.442838000
C	-1.141137000	5.209781000	-2.082487000
C	0.038846000	4.836784000	-2.711407000
C	-7.949446000	0.238620000	-2.823828000
H	-4.823375000	5.151386000	-0.980314000
H	-8.230191000	4.330654000	-0.511793000
H	-9.216807000	2.213690000	-1.422950000
H	-5.386559000	1.168634000	-3.076764000
H	-0.823605000	1.789525000	-4.112304000
H	1.113894000	3.384597000	-3.908206000
H	-1.213916000	6.130810000	-1.501997000
H	0.903703000	5.498312000	-2.634279000
H	-7.472323000	-0.609560000	-2.307419000
H	-9.033419000	0.185125000	-2.648533000
H	-7.760754000	0.111983000	-3.903873000
O	7.658427000	1.259261000	-2.273518000
N	10.154199000	-1.393308000	-4.427422000
N	9.949859000	0.563253000	-3.278405000
N	9.370401000	-0.565574000	-3.738500000
C	7.193887000	0.089147000	-2.785411000
C	5.843333000	-0.231225000	-2.585128000
C	5.304593000	-1.412189000	-3.088745000

C	6.090941000	-2.323417000	-3.814638000
C	7.435365000	-2.012104000	-4.018954000
C	7.991791000	-0.829334000	-3.514107000
C	11.229526000	0.469002000	-3.716281000
C	11.352078000	-0.754458000	-4.441922000
C	12.565061000	-1.128501000	-5.062185000
C	13.622482000	-0.239974000	-4.940383000
C	12.325819000	1.349701000	-3.580032000
C	13.505803000	0.976703000	-4.208952000
C	5.517509000	-3.621460000	-4.321373000
H	8.643580000	1.291306000	-2.477859000
H	5.236751000	0.470580000	-2.009344000
H	4.250137000	-1.646375000	-2.920465000
H	8.080397000	-2.691447000	-4.574309000
H	12.643351000	-2.070556000	-5.609849000
H	14.580850000	-0.475484000	-5.405751000
H	12.253040000	2.270730000	-2.999542000
H	14.370659000	1.638232000	-4.131824000
H	5.994634000	-4.469641000	-3.804964000
H	4.433537000	-3.674955000	-4.146078000
H	5.706201000	-3.748097000	-5.401418000
O	1.203017000	7.432396000	-0.816282000
N	2.604015000	9.632099000	2.515525000
N	2.656130000	7.693194000	1.320511000
N	2.171917000	8.945398000	1.459197000
C	0.792297000	8.704090000	-0.568892000
C	-0.125519000	9.286640000	-1.454157000
C	-0.589352000	10.584824000	-1.253953000
C	-0.157897000	11.354796000	-0.160054000
C	0.754415000	10.781721000	0.726177000
C	1.232900000	9.477983000	0.534487000
C	3.473362000	7.545542000	2.392516000
C	3.432848000	8.759095000	3.141999000
C	4.154590000	8.908190000	4.348263000
C	4.891739000	7.811117000	4.767777000
C	4.251557000	6.447027000	2.821732000
C	4.941820000	6.602165000	4.016222000
C	-0.627423000	12.772886000	0.031967000
H	1.882663000	7.206406000	-0.108477000
H	-0.449432000	8.692771000	-2.311117000
H	-1.302834000	11.023162000	-1.955958000
H	1.115753000	11.343407000	1.586064000
H	4.116478000	9.845193000	4.907118000
H	5.463233000	7.869175000	5.695422000

H	4.316508000	5.525979000	2.239315000
H	5.545935000	5.770248000	4.382972000
H	0.220028000	13.468356000	-0.084634000
H	-1.398475000	13.042476000	-0.704298000
H	-1.038744000	12.925347000	1.044740000
O	-7.089875000	7.565995000	4.403693000
N	-4.594103000	4.913425000	2.249789000
N	-4.798443000	6.869986000	3.398806000
N	-5.377901000	5.741160000	2.938710000
C	-7.554415000	6.395880000	3.891800000
C	-8.904975000	6.075511000	4.092075000
C	-9.443706000	4.894543000	3.588488000
C	-8.657364000	3.983323000	2.862563000
C	-7.313004000	4.294385000	2.658479000
C	-6.756524000	5.477425000	3.163058000
C	-3.518777000	6.775735000	2.960929000
C	-3.396221000	5.552270000	2.235298000
C	-2.183255000	5.178246000	1.615001000
C	-1.125819000	6.066757000	1.736833000
C	-2.422484000	7.656435000	3.097178000
C	-1.242501000	7.283437000	2.468258000
C	-9.230793000	2.685274000	2.355838000
H	-6.104722000	7.598039000	4.199352000
H	-9.511537000	6.777307000	4.667873000
H	-10.498154000	4.660343000	3.756716000
H	-6.667782000	3.615525000	2.102698000
H	-2.104957000	4.236176000	1.067378000
H	-0.167452000	5.831250000	1.271460000
H	-2.495263000	8.577464000	3.677668000
H	-0.377644000	7.944965000	2.545387000
H	-8.753670000	1.837092000	2.872246000
H	-10.314766000	2.631778000	2.531132000
H	-9.042101000	2.558636000	1.275793000

S_1 (enol)

O	0.132237000	-0.830408000	-1.855169000
N	-2.318388000	1.708799000	0.365588000
N	-2.094780000	-0.199645000	-0.926231000
N	-1.501003000	0.926788000	-0.389093000
C	0.635408000	0.294184000	-1.381214000
C	1.980545000	0.609632000	-1.621732000
C	2.532855000	1.768543000	-1.105866000
C	1.752834000	2.649017000	-0.326148000
C	0.414344000	2.337435000	-0.052703000

C	-0.177562000	1.202156000	-0.588680000
C	-3.356150000	-0.139065000	-0.466933000
C	-3.483291000	1.044424000	0.338226000
C	-4.697949000	1.350418000	0.985409000
C	-5.741327000	0.455589000	0.832321000
C	-4.449121000	-1.000717000	-0.655969000
C	-5.620935000	-0.696725000	0.017851000
C	2.328136000	3.941884000	0.155313000
H	-0.893354000	-0.824909000	-1.632308000
H	2.553700000	-0.078502000	-2.231170000
H	3.571781000	2.014160000	-1.289285000
H	-0.187654000	2.989382000	0.562691000
H	-4.777763000	2.255833000	1.576657000
H	-6.678178000	0.626106000	1.350732000
H	-4.391033000	-1.863135000	-1.307040000
H	-6.459678000	-1.369501000	-0.108750000
H	2.001525000	4.753696000	-0.504653000
H	3.418732000	3.923347000	0.158886000
H	1.965333000	4.186157000	1.156954000
O	4.088440000	-2.031768000	6.979383000
N	2.687224000	-4.230986000	3.648447000
N	2.635351000	-2.292498000	4.842578000
N	3.119548000	-3.544708000	4.703892000
C	4.498958000	-3.302999000	6.732860000
C	5.416978000	-3.885980000	7.617247000
C	5.880577000	-5.183754000	7.417925000
C	5.449329000	-5.954137000	6.323144000
C	4.537242000	-5.380148000	5.436540000
C	4.058557000	-4.077305000	5.628602000
C	1.817904000	-2.144419000	3.771455000
C	1.858617000	-3.358387000	3.021091000
C	1.136656000	-3.507057000	1.815709000
C	0.399740000	-2.410391000	1.395313000
C	1.039722000	-1.045892000	3.342239000
C	0.349457000	-1.201021000	2.147749000
C	5.918837000	-7.372233000	6.131122000
H	3.408852000	-1.805692000	6.271569000
H	5.740899000	-3.292116000	8.474206000
H	6.594272000	-5.622518000	8.119047000
H	4.175679000	-5.942731000	4.577025000
H	1.174974000	-4.444476000	1.255971000
H	-0.171754000	-2.468441000	0.467667000
H	0.975002000	-0.125260000	3.923774000
H	-0.254428000	-0.369512000	1.780117000

H	5.071377000	-8.067691000	6.247723000
H	6.689885000	-7.641833000	6.867387000
H	6.329938000	-7.524282000	5.119231000
O	-8.097151000	-0.618181000	3.297262000
N	-9.498368000	-2.817398000	-0.033675000
N	-9.550240000	-0.878909000	1.160457000
N	-9.066043000	-2.131120000	1.021771000
C	-7.686633000	-1.889410000	3.050739000
C	-6.768612000	-2.472391000	3.935126000
C	-6.305014000	-3.770165000	3.735804000
C	-6.736261000	-4.540548000	2.641023000
C	-7.648349000	-3.966560000	1.754419000
C	-8.127033000	-2.663717000	1.946481000
C	-10.367688000	-0.730830000	0.089334000
C	-10.326972000	-1.944800000	-0.661030000
C	-11.048934000	-2.093469000	-1.866412000
C	-11.785850000	-0.996803000	-2.286808000
C	-11.145868000	0.367696000	-0.339882000
C	-11.836133000	0.212567000	-1.534372000
C	-6.266754000	-5.958645000	2.449001000
H	-8.776738000	-0.392104000	2.589448000
H	-6.444691000	-1.878527000	4.792085000
H	-5.591320000	-4.208929000	4.436926000
H	-8.009911000	-4.529143000	0.894904000
H	-11.010616000	-3.030889000	-2.426150000
H	-12.357344000	-1.054852000	-3.214454000
H	-11.210589000	1.288330000	0.241653000
H	-12.440019000	1.044075000	-1.902004000
H	-7.114213000	-6.654104000	2.565602000
H	-5.495706000	-6.228245000	3.185266000
H	-5.855653000	-6.110694000	1.437110000
O	-1.382351000	-6.571089000	2.366780000
N	0.018867000	-4.371871000	5.697717000
N	0.070954000	-6.309457000	4.503212000
N	-0.413460000	-5.058148000	4.642270000
C	-1.792869000	-5.299859000	2.613303000
C	-2.710455000	-4.716392000	1.727661000
C	-3.174488000	-3.419104000	1.928238000
C	-2.743023000	-2.649137000	3.022137000
C	-1.830936000	-3.221807000	3.909251000
C	-1.352251000	-4.524649000	3.717188000
C	0.888184000	-6.458438000	5.574708000
C	0.847471000	-5.244469000	6.325072000
C	1.569649000	-5.094897000	7.530082000

C	2.306566000	-6.191563000	7.950478000
C	1.666366000	-7.556965000	6.003924000
C	2.356849000	-7.400934000	7.198041000
C	-3.212531000	-1.229721000	3.214668000
H	-0.702328000	-6.796679000	3.073339000
H	-3.034593000	-5.309839000	0.871584000
H	-3.887965000	-2.980756000	1.226234000
H	-1.469591000	-2.660127000	4.769138000
H	1.531331000	-4.157478000	8.089820000
H	2.877842000	-6.134416000	8.878496000
H	1.731304000	-8.476696000	5.422016000
H	2.960518000	-8.233344000	7.566046000
H	-2.365071000	-0.535583000	3.097558000
H	-3.983578000	-0.961440000	2.477893000
H	-3.623632000	-1.077672000	4.226559000
O	5.369754000	-4.478439000	1.799717000
N	3.968537000	-6.677656000	-1.531220000
N	3.916665000	-4.739169000	-0.337087000
N	4.400863000	-5.991379000	-0.475773000
C	5.780273000	-5.749668000	1.553194000
C	6.698292000	-6.332650000	2.437581000
C	7.161891000	-7.630423000	2.238259000
C	6.730644000	-8.400806000	1.143478000
C	5.818556000	-7.826819000	0.256874000
C	5.339871000	-6.523976000	0.448936000
C	3.099218000	-4.591088000	-1.408211000
C	3.139932000	-5.805058000	-2.158575000
C	2.417971000	-5.953728000	-3.363957000
C	1.681076000	-4.857052000	-3.784366000
C	2.320988000	-3.492584000	-1.837395000
C	1.630944000	-3.647598000	-3.031991000
C	7.200152000	-9.818904000	0.951456000
H	4.690167000	-4.252363000	1.091903000
H	7.022213000	-5.738787000	3.294540000
H	7.875585000	-8.069188000	2.939381000
H	5.456994000	-8.389401000	-0.602641000
H	2.456289000	-6.891147000	-3.923695000
H	1.109561000	-4.915111000	-4.711999000
H	2.256317000	-2.571930000	-1.255891000
H	1.026751000	-2.816408000	-3.399478000
H	6.352692000	-10.514362000	1.068057000
H	7.971199000	-10.088503000	1.687721000
H	7.611253000	-9.970952000	-0.060435000
O	-6.815842000	-3.064835000	-1.882361000

N	-8.217055000	-5.264069000	-5.213341000
N	-8.268925000	-3.325580000	-4.019208000
N	-7.784729000	-4.577791000	-4.157894000
C	-6.405313000	-4.336078000	-2.128927000
C	-5.487297000	-4.919062000	-1.244540000
C	-5.023699000	-6.216835000	-1.443862000
C	-5.454947000	-6.987219000	-2.538643000
C	-6.367034000	-6.413231000	-3.425247000
C	-6.845719000	-5.110388000	-3.233185000
C	-9.086373000	-3.177501000	-5.090332000
C	-9.045658000	-4.391470000	-5.840696000
C	-9.767619000	-4.540140000	-7.046078000
C	-10.504536000	-3.443474000	-7.466474000
C	-9.864554000	-2.078974000	-5.519547000
C	-10.554820000	-2.234103000	-6.714038000
C	-4.985439000	-8.405316000	-2.730665000
H	-7.495416000	-2.838778000	-2.590226000
H	-5.163377000	-4.325198000	-0.387581000
H	-4.310005000	-6.655600000	-0.742740000
H	-6.728596000	-6.975813000	-4.284762000
H	-9.729302000	-5.477559000	-7.605816000
H	-11.076030000	-3.501523000	-8.394120000
H	-9.929274000	-1.158341000	-4.938012000
H	-11.158705000	-1.402595000	-7.081670000
H	-5.832900000	-9.100773000	-2.614064000
H	-4.214391000	-8.674916000	-1.994400000
H	-4.574338000	-8.557365000	-3.742556000
O	1.477299000	-3.197607000	-7.102763000
N	-1.018655000	-0.545907000	-4.948486000
N	-0.814559000	-2.502054000	-6.096621000
N	-0.235086000	-1.373236000	-5.636526000
C	1.941637000	-2.028401000	-6.590498000
C	3.292201000	-1.708049000	-6.790773000
C	3.830948000	-0.527089000	-6.287186000
C	3.044615000	0.384147000	-5.561269000
C	1.699970000	0.073270000	-5.356072000
C	1.143527000	-1.109492000	-5.860919000
C	-2.094225000	-2.407786000	-5.658744000
C	-2.216325000	-1.183839000	-4.934358000
C	-3.429739000	-0.810265000	-4.312841000
C	-4.487172000	-1.698777000	-4.434643000
C	-3.190094000	-3.287985000	-5.796249000
C	-4.370290000	-2.915874000	-5.166956000
C	3.618064000	1.682184000	-5.054535000

H	0.491928000	-3.230540000	-6.898049000
H	3.898754000	-2.409854000	-7.366571000
H	4.885399000	-0.292903000	-6.455413000
H	1.054947000	0.752621000	-4.800717000
H	-3.507797000	0.131373000	-3.766060000
H	-5.445536000	-1.463257000	-3.969275000
H	-3.117762000	-4.209502000	-6.375484000
H	-5.235372000	-3.576973000	-5.243202000
H	3.140952000	2.530371000	-5.570944000
H	4.702255000	1.736567000	-5.230203000
H	3.429374000	1.808823000	-3.974490000
O	-0.101036000	-9.017758000	-2.812886000
N	1.300181000	-6.818541000	0.518051000
N	1.352268000	-8.756128000	-0.676454000
N	0.867855000	-7.504819000	-0.537395000
C	-0.511556000	-7.746529000	-2.566363000
C	-1.429140000	-7.163062000	-3.452004000
C	-1.893174000	-5.865774000	-3.251428000
C	-1.461703000	-5.095811000	-2.157501000
C	-0.549623000	-5.668477000	-1.270415000
C	-0.070936000	-6.971319000	-1.462477000
C	2.169499000	-8.905109000	0.395042000
C	2.128785000	-7.691139000	1.145407000
C	2.850964000	-7.541568000	2.350416000
C	3.587881000	-8.638234000	2.770812000
C	2.947681000	-10.003635000	0.824258000
C	3.638164000	-9.847604000	2.018375000
C	-1.931194000	-3.676391000	-1.964828000
H	0.578987000	-9.243350000	-2.106327000
H	-1.753279000	-7.756510000	-4.308081000
H	-2.606650000	-5.427427000	-3.953432000
H	-0.188277000	-5.106796000	-0.410528000
H	2.812646000	-6.604148000	2.910154000
H	4.159156000	-8.581087000	3.698830000
H	3.012618000	-10.923366000	0.242350000
H	4.241831000	-10.680014000	2.386380000
H	-1.083754000	-2.981954000	-2.082317000
H	-2.702238000	-3.408149000	-2.701813000
H	-2.342330000	-3.524330000	-0.953113000
O	-1.085330000	1.695734000	3.256569000
N	-3.581285000	4.347434000	5.410845000
N	-3.377189000	2.391287000	4.262711000
N	-2.797715000	3.520106000	4.722806000
C	-0.620986000	2.864931000	3.768851000

C	0.729571000	3.185298000	3.568550000
C	1.268314000	4.366260000	4.072131000
C	0.481987000	5.277488000	4.798063000
C	-0.862659000	4.966611000	5.003259000
C	-1.419101000	3.783849000	4.498412000
C	-4.656853000	2.485555000	4.700587000
C	-4.778954000	3.709501000	5.424973000
C	-5.992367000	4.083076000	6.046491000
C	-7.049800000	3.194563000	5.924688000
C	-5.752723000	1.605355000	4.563083000
C	-6.932918000	1.977466000	5.192376000
C	1.055435000	6.575524000	5.304796000
H	-2.070701000	1.662801000	3.461282000
H	1.336086000	2.483496000	2.992728000
H	2.322770000	4.600438000	3.903918000
H	-1.507682000	5.645961000	5.558614000
H	-6.070427000	5.024714000	6.593272000
H	-8.008165000	3.430084000	6.390056000
H	-5.680391000	0.683840000	3.983848000
H	-7.798002000	1.316367000	5.116129000
H	0.578323000	7.423712000	4.788387000
H	2.139627000	6.629907000	5.129129000
H	0.866745000	6.702165000	6.384841000
O	6.378208000	3.706246000	2.905428000
N	8.873945000	1.053643000	0.751524000
N	8.669631000	3.010207000	1.900541000
N	8.090157000	1.881388000	1.440446000
C	5.913652000	2.536137000	2.393536000
C	4.563088000	2.215786000	2.593810000
C	4.024338000	1.034830000	2.090201000
C	4.810675000	0.123589000	1.364307000
C	6.155102000	0.434883000	1.159992000
C	6.711543000	1.617644000	1.664839000
C	9.949295000	2.915939000	1.462664000
C	10.071831000	1.692477000	0.737024000
C	11.284809000	1.318418000	0.116761000
C	12.342242000	2.206930000	0.238563000
C	11.045600000	3.796623000	1.598914000
C	12.225579000	3.423611000	0.969994000
C	4.237225000	-1.174447000	0.857573000
H	7.363362000	3.738276000	2.701087000
H	3.956534000	2.917590000	3.169608000
H	2.969905000	0.800640000	2.258480000
H	6.800126000	-0.244469000	0.604637000

H	11.363087000	0.376363000	-0.430903000
H	13.300607000	1.971409000	-0.226805000
H	10.972833000	4.717654000	2.179404000
H	13.090445000	4.085126000	1.047122000
H	4.714338000	-2.022635000	1.373982000
H	3.153252000	-1.227928000	1.032868000
H	4.425916000	-1.301087000	-0.222472000
O	2.485536000	4.986174000	-5.996679000
N	3.886535000	7.185809000	-2.664860000
N	3.938623000	5.246905000	-3.859875000
N	3.454428000	6.499115000	-3.721189000
C	2.074799000	6.257822000	-5.749274000
C	1.156996000	6.840387000	-6.634543000
C	0.693180000	8.138577000	-6.434339000
C	1.124646000	8.908542000	-5.340440000
C	2.036950000	8.335456000	-4.454209000
C	2.515418000	7.031712000	-4.645899000
C	4.755854000	5.099241000	-2.787869000
C	4.715356000	6.312794000	-2.038387000
C	5.437100000	6.461880000	-0.832123000
C	6.174235000	5.364798000	-0.412609000
C	5.534034000	4.000716000	-2.358654000
C	6.224300000	4.155844000	-1.164164000
C	0.655138000	10.326641000	-5.148419000
H	3.165122000	4.760098000	-5.288866000
H	0.833075000	6.246522000	-7.491503000
H	-0.020297000	8.576924000	-7.136343000
H	2.398296000	8.897137000	-3.594321000
H	5.399001000	7.398883000	-0.273267000
H	6.745729000	5.422848000	0.515036000
H	5.598974000	3.079666000	-2.941071000
H	6.828404000	3.323920000	-0.797414000
H	1.502598000	11.022097000	-5.265019000
H	-0.115910000	10.596240000	-5.884684000
H	0.243819000	10.479105000	-4.135645000
O	-5.807382000	5.119834000	-0.776693000
N	-3.311645000	2.467231000	-2.930597000
N	-3.515960000	4.423795000	-1.781580000
N	-4.095435000	3.294975000	-2.241675000
C	-6.271938000	3.949725000	-1.288586000
C	-7.622503000	3.629374000	-1.088311000
C	-8.161249000	2.448414000	-1.591898000
C	-7.374917000	1.537177000	-2.317815000
C	-6.030489000	1.848471000	-2.522129000

C	-5.474047000	3.031234000	-2.017282000
C	-2.236295000	4.329527000	-2.219457000
C	-2.113759000	3.106066000	-2.945097000
C	-0.900781000	2.732006000	-3.565360000
C	0.156650000	3.620522000	-3.443562000
C	-1.139988000	5.210207000	-2.083200000
C	0.039988000	4.837200000	-2.712137000
C	-7.948369000	0.239134000	-2.824556000
H	-4.822229000	5.151865000	-0.981034000
H	-8.229056000	4.331178000	-0.512513000
H	-9.215700000	2.214227000	-1.423670000
H	-5.385466000	1.169120000	-3.077484000
H	-0.822503000	1.789951000	-4.113024000
H	1.115017000	3.384998000	-3.908926000
H	-1.212757000	6.131241000	-1.502717000
H	0.904789000	5.498736000	-2.634970000
H	-7.471239000	-0.609035000	-2.308153000
H	-9.032338000	0.185662000	-2.649250000
H	-7.759681000	0.112497000	-3.904594000
O	7.659522000	1.259575000	-2.274238000
N	10.155258000	-1.393027000	-4.428142000
N	9.950944000	0.563537000	-3.279125000
N	9.371471000	-0.565283000	-3.739220000
C	7.194963000	0.089471000	-2.786143000
C	5.844443000	-0.230867000	-2.585834000
C	5.305637000	-1.411811000	-3.089502000
C	6.091979000	-2.323064000	-3.815391000
C	7.436416000	-2.011787000	-4.019674000
C	7.992858000	-0.829024000	-3.514827000
C	11.230610000	0.469269000	-3.717001000
C	11.353146000	-0.754193000	-4.442642000
C	12.566124000	-1.128252000	-5.062905000
C	13.623557000	-0.239739000	-4.941103000
C	12.326915000	1.349953000	-3.580752000
C	13.506894000	0.976940000	-4.209672000
C	5.518539000	-3.621118000	-4.322093000
H	8.644675000	1.291607000	-2.478579000
H	5.237775000	0.470884000	-2.010032000
H	4.251189000	-1.646077000	-2.921081000
H	8.081439000	-2.691139000	-4.575029000
H	12.644401000	-2.070308000	-5.610569000
H	14.581922000	-0.475262000	-5.406471000
H	12.254148000	2.270983000	-3.000262000
H	14.371759000	1.638457000	-4.132544000

H	5.995653000	-4.469305000	-3.805684000
H	4.434566000	-3.674598000	-4.146798000
H	5.707229000	-3.747757000	-5.402138000
O	1.204219000	7.432812000	-0.817021000
N	2.605220000	9.632480000	2.514805000
N	2.657310000	7.693574000	1.319791000
N	2.173113000	8.945785000	1.458477000
C	0.793494000	8.704496000	-0.569615000
C	-0.124318000	9.287057000	-1.454877000
C	-0.588134000	10.585247000	-1.254673000
C	-0.156669000	11.355213000	-0.160774000
C	0.755635000	10.782126000	0.725457000
C	1.234103000	9.478382000	0.533767000
C	3.474540000	7.545911000	2.391796000
C	3.434042000	8.759465000	3.141279000
C	4.155786000	8.908550000	4.347543000
C	4.892920000	7.811468000	4.767057000
C	4.252720000	6.447386000	2.821012000
C	4.942985000	6.602515000	4.015502000
C	-0.626176000	12.773310000	0.031247000
H	1.883845000	7.206688000	-0.109200000
H	-0.448239000	8.693192000	-2.311837000
H	-1.301610000	11.023594000	-1.956678000
H	1.116981000	11.343807000	1.585344000
H	4.117686000	9.845554000	4.906398000
H	5.464415000	7.869518000	5.694702000
H	4.317659000	5.526337000	2.238595000
H	5.547089000	5.770590000	4.382252000
H	0.221284000	13.468768000	-0.085354000
H	-1.397225000	13.042910000	-0.705018000
H	-1.037495000	12.925776000	1.044020000
O	-7.088697000	7.566504000	4.402973000
N	-4.592960000	4.913901000	2.249069000
N	-4.797274000	6.870465000	3.398086000
N	-5.376747000	5.741646000	2.937990000
C	-7.553253000	6.396395000	3.891080000
C	-8.903817000	6.076044000	4.091355000
C	-9.442563000	4.895083000	3.587768000
C	-8.656234000	3.983855000	2.861840000
C	-7.311855000	4.294924000	2.657743000
C	-6.755373000	5.477928000	3.162342000
C	-3.517610000	6.776197000	2.960209000
C	-3.395064000	5.552721000	2.234594000
C	-2.182117000	5.178686000	1.614287000

C	-1.124663000	6.067191000	1.736106000
C	-2.421305000	7.656882000	3.096458000
C	-1.241327000	7.283869000	2.467538000
C	-9.229680000	2.685811000	2.355118000
H	-6.103544000	7.598535000	4.198632000
H	-9.510369000	6.777848000	4.667153000
H	-10.497014000	4.660897000	3.755996000
H	-6.666678000	3.615995000	2.101991000
H	-2.103775000	4.236541000	1.066659000
H	-0.166297000	5.831667000	1.270740000
H	-2.494072000	8.577912000	3.676948000
H	-0.376461000	7.945385000	2.544667000
H	-8.752568000	1.837623000	2.871526000
H	-10.313653000	2.632330000	2.530412000
H	-9.040989000	2.559171000	1.275073000

S_1 (keto)

O	0.227985000	-1.056297000	-1.493701000
N	-2.275056000	1.507767000	0.482483000
N	-2.195012000	-0.087568000	-1.094443000
N	-1.482707000	0.770414000	-0.289131000
C	0.678559000	0.136865000	-1.265671000
C	1.989812000	0.536797000	-1.583773000
C	2.517091000	1.770812000	-1.159373000
C	1.738496000	2.674118000	-0.475802000
C	0.358032000	2.340104000	-0.255016000
C	-0.130867000	1.131302000	-0.616680000
C	-3.454483000	-0.111793000	-0.579402000
C	-3.492917000	0.955855000	0.358715000
C	-4.685167000	1.262513000	1.050174000
C	-5.758806000	0.422514000	0.846759000
C	-4.554611000	-0.940878000	-0.797757000
C	-5.689439000	-0.672435000	-0.047431000
C	2.266114000	3.975200000	0.040690000
H	-1.602918000	-0.841873000	-1.480335000
H	2.610752000	-0.176810000	-2.111986000
H	3.560528000	2.003085000	-1.341226000
H	-0.290012000	3.050149000	0.241085000
H	-4.713456000	2.109388000	1.726347000
H	-6.674526000	0.574370000	1.406380000
H	-4.536869000	-1.763584000	-1.499252000
H	-6.533793000	-1.337774000	-0.170599000
H	1.758870000	4.815505000	-0.444152000
H	3.339303000	4.067695000	-0.130704000

H	2.076620000	4.074375000	1.114942000
O	4.090164000	-2.032190000	6.978770000
N	2.687667000	-4.229787000	3.647304000
N	2.636701000	-2.291664000	4.842067000
N	3.120393000	-3.544019000	4.702915000
C	4.500155000	-3.303502000	6.731785000
C	5.418062000	-3.887125000	7.615867000
C	5.881129000	-5.185015000	7.416067000
C	5.449442000	-5.954876000	6.321093000
C	4.537465000	-5.380246000	5.434790000
C	4.059312000	-4.077280000	5.627333000
C	1.819176000	-2.142921000	3.771096000
C	1.859321000	-3.356664000	3.020335000
C	1.137149000	-3.504663000	1.814997000
C	0.400605000	-2.407574000	1.395049000
C	1.041367000	-1.043954000	3.342334000
C	0.350889000	-1.198429000	2.147882000
C	5.918373000	-7.373094000	6.128553000
H	3.410574000	-1.805621000	6.271116000
H	5.742323000	-3.293663000	8.472976000
H	6.594742000	-5.624284000	8.116956000
H	4.175574000	-5.942412000	4.575140000
H	1.175029000	-4.441917000	1.254952000
H	-0.171030000	-2.465104000	0.467457000
H	0.977080000	-0.123484000	3.924174000
H	-0.252719000	-0.366566000	1.780595000
H	5.070657000	-8.068259000	6.245038000
H	6.689409000	-7.643232000	6.864633000
H	6.329286000	-7.524977000	5.116561000
O	-8.095345000	-0.612668000	3.298660000
N	-9.497843000	-2.810266000	-0.032807000
N	-9.548806000	-0.872142000	1.161956000
N	-9.065116000	-2.124496000	1.022805000
C	-7.685353000	-1.883978000	3.051675000
C	-6.767447000	-2.467602000	3.935756000
C	-6.304380000	-3.765492000	3.735957000
C	-6.736066000	-4.535353000	2.640982000
C	-7.648043000	-3.960724000	1.754680000
C	-8.126195000	-2.657758000	1.947223000
C	-10.366333000	-0.723398000	0.090986000
C	-10.326187000	-1.937143000	-0.659775000
C	-11.048360000	-2.085141000	-1.865113000
C	-11.784902000	-0.988052000	-2.285061000
C	-11.144141000	0.375568000	-0.337777000

C	-11.834619000	0.221094000	-1.532229000
C	-6.267136000	-5.953572000	2.448443000
H	-8.774934000	-0.386098000	2.591006000
H	-6.443185000	-1.874141000	4.792866000
H	-5.590767000	-4.204760000	4.436846000
H	-8.009934000	-4.522889000	0.895030000
H	-11.010479000	-3.022396000	-2.425158000
H	-12.356537000	-1.045580000	-3.212653000
H	-11.208429000	1.296040000	0.244063000
H	-12.438227000	1.052955000	-1.899515000
H	-7.114851000	-6.648737000	2.564928000
H	-5.496099000	-6.223709000	3.184523000
H	-5.856223000	-6.105454000	1.436451000
O	-1.382982000	-6.567892000	2.365401000
N	0.019517000	-4.370294000	5.696869000
N	0.070697000	-6.307515000	4.501732000
N	-0.413212000	-5.056063000	4.641256000
C	-1.792974000	-5.296581000	2.612387000
C	-2.710444000	-4.712471000	1.727050000
C	-3.173947000	-3.415067000	1.928105000
C	-2.742043000	-2.645622000	3.022197000
C	-1.830066000	-3.218933000	3.909010000
C	-1.351913000	-4.521899000	3.716466000
C	0.888005000	-6.457161000	5.573076000
C	0.847860000	-5.243417000	6.323837000
C	1.570250000	-5.094516000	7.528803000
C	2.306794000	-6.191604000	7.948751000
C	1.665814000	-7.556128000	6.001838000
C	2.356510000	-7.400751000	7.195917000
C	-3.210974000	-1.226085000	3.215246000
H	-0.702957000	-6.793975000	3.071801000
H	-3.034924000	-5.305516000	0.870823000
H	-3.887342000	-2.976215000	1.226333000
H	-1.468393000	-2.657671000	4.769032000
H	1.532369000	-4.157262000	8.088848000
H	2.878210000	-6.134978000	8.876714000
H	1.730319000	-8.475696000	5.419625000
H	2.959901000	-8.233515000	7.563577000
H	-2.363258000	-0.532240000	3.098252000
H	-3.982010000	-0.957266000	2.478656000
H	-3.621887000	-1.074202000	4.227238000
O	5.369865000	-4.477688000	1.798152000
N	3.967366000	-6.675286000	-1.533315000
N	3.916402000	-4.737163000	-0.338551000

N	4.400095000	-5.989518000	-0.477702000
C	5.779857000	-5.748998000	1.551167000
C	6.697762000	-6.332623000	2.435248000
C	7.160830000	-7.630512000	2.235449000
C	6.729142000	-8.400374000	1.140474000
C	5.817166000	-7.825745000	0.254172000
C	5.339012000	-6.522779000	0.446715000
C	3.098876000	-4.588418000	-1.409522000
C	3.139022000	-5.802163000	-2.160283000
C	2.416849000	-5.950162000	-3.365621000
C	1.680328000	-4.853063000	-3.785583000
C	2.321020000	-3.489474000	-1.838253000
C	1.630775000	-3.643815000	-3.032789000
C	7.198075000	-9.818593000	0.947935000
H	4.690276000	-4.251119000	1.090498000
H	7.022023000	-5.739162000	3.292358000
H	7.874442000	-8.069781000	2.936338000
H	5.455275000	-8.387910000	-0.605478000
H	2.454731000	-6.887416000	-3.925666000
H	1.108672000	-4.910601000	-4.713161000
H	2.256781000	-2.568981000	-1.256444000
H	1.026810000	-2.812293000	-3.400013000
H	6.350358000	-10.513758000	1.064420000
H	7.969110000	-10.088730000	1.684015000
H	7.608987000	-9.970474000	-0.064057000
O	-6.815655000	-3.058147000	-1.881941000
N	-8.218143000	-5.255764000	-5.213426000
N	-8.269106000	-3.317641000	-4.018661000
N	-7.785415000	-4.569994000	-4.157813000
C	-6.405653000	-4.329479000	-2.128930000
C	-5.487740000	-4.913098000	-1.244867000
C	-5.024678000	-6.210990000	-1.444662000
C	-5.456365000	-6.980852000	-2.539636000
C	-6.368342000	-6.406223000	-3.425938000
C	-6.846490000	-5.103254000	-3.233399000
C	-9.086632000	-3.168897000	-5.089633000
C	-9.046486000	-4.382640000	-5.840393000
C	-9.768658000	-4.530639000	-7.045731000
C	-10.505202000	-3.433551000	-7.465679000
C	-9.864439000	-2.069929000	-5.518394000
C	-10.554918000	-2.224404000	-6.712847000
C	-4.987435000	-8.399070000	-2.732175000
H	-7.495232000	-2.831607000	-2.589616000
H	-5.163484000	-4.319640000	-0.387753000

H	-4.311065000	-6.650259000	-0.743772000
H	-6.730233000	-6.968387000	-4.285589000
H	-9.730778000	-5.467894000	-7.605776000
H	-11.076836000	-3.491079000	-8.393271000
H	-9.928727000	-1.149459000	-4.936554000
H	-11.158526000	-1.392542000	-7.080134000
H	-5.835151000	-9.094234000	-2.615691000
H	-4.216397000	-8.669208000	-1.996096000
H	-4.576522000	-8.550953000	-3.744168000
O	1.476774000	-3.192469000	-7.103418000
N	-1.017872000	-0.540492000	-4.947967000
N	-0.814684000	-2.496348000	-6.096759000
N	-0.234713000	-1.367904000	-5.636374000
C	1.941633000	-2.023610000	-6.590835000
C	3.292297000	-1.703719000	-6.791179000
C	3.831567000	-0.523132000	-6.287280000
C	3.045682000	0.388176000	-5.560968000
C	1.700942000	0.077757000	-5.355700000
C	1.143974000	-1.104624000	-5.860858000
C	-2.094258000	-2.401723000	-5.658688000
C	-2.215789000	-1.177963000	-4.933892000
C	-3.428978000	-0.804116000	-4.312100000
C	-4.486771000	-1.692177000	-4.434054000
C	-3.190486000	-3.281450000	-5.796338000
C	-4.370458000	-2.909083000	-5.166774000
C	3.619702000	1.685827000	-5.053889000
H	0.491417000	-3.225085000	-6.898589000
H	3.898502000	-2.405575000	-7.367280000
H	4.886088000	-0.289302000	-6.455566000
H	1.056254000	0.757181000	-4.800044000
H	-3.506599000	0.137376000	-3.765005000
H	-5.444985000	-1.456434000	-3.968487000
H	-3.118588000	-4.202808000	-6.375879000
H	-5.235807000	-3.569820000	-5.243123000
H	3.142855000	2.534366000	-5.569963000
H	4.703891000	1.739844000	-5.229678000
H	3.431198000	1.812190000	-3.973779000
O	-0.103282000	-9.013389000	-2.815217000
N	1.299217000	-6.815792000	0.516250000
N	1.350398000	-8.753013000	-0.678886000
N	0.866489000	-7.501562000	-0.539362000
C	-0.513274000	-7.742079000	-2.568232000
C	-1.430743000	-7.157969000	-3.453567000
C	-1.894247000	-5.860565000	-3.252514000

C	-1.462335000	-5.091124000	-2.158385000
C	-0.550366000	-5.664431000	-1.271608000
C	-0.072211000	-6.967397000	-1.464152000
C	2.167707000	-8.902659000	0.392458000
C	2.127561000	-7.688915000	1.143219000
C	2.849952000	-7.540015000	2.348184000
C	3.586495000	-8.637103000	2.768133000
C	2.945515000	-10.001626000	0.821220000
C	3.636211000	-9.846249000	2.015299000
C	-1.931534000	-3.671279000	-1.965464000
H	0.576744000	-9.239474000	-2.108818000
H	-1.755223000	-7.751014000	-4.309794000
H	-2.607641000	-5.421714000	-3.954285000
H	-0.188692000	-5.103169000	-0.411586000
H	2.812070000	-6.602759000	2.908230000
H	4.157911000	-8.580477000	3.696096000
H	3.010020000	-10.921194000	0.239007000
H	4.239600000	-10.679012000	2.382959000
H	-1.083203000	-2.977805000	-2.082288000
H	-2.702302000	-3.402775000	-2.701972000
H	-2.342143000	-3.519749000	-0.953355000
O	-1.082627000	1.698527000	3.257819000
N	-3.577275000	4.350505000	5.413269000
N	-3.374087000	2.394649000	4.264478000
N	-2.794115000	3.523094000	4.724863000
C	-0.617764000	2.867379000	3.770419000
C	0.732894000	3.187283000	3.570049000
C	1.272161000	4.367874000	4.073942000
C	0.486280000	5.279174000	4.800268000
C	-0.858461000	4.968753000	5.005536000
C	-1.415427000	3.786372000	4.500378000
C	-4.653659000	2.489274000	4.702547000
C	-4.775190000	3.713035000	5.427344000
C	-5.988378000	4.086881000	6.049137000
C	-7.046173000	3.198819000	5.927182000
C	-5.749888000	1.609546000	4.564899000
C	-6.929858000	1.981913000	5.194463000
C	1.060299000	6.576823000	5.307347000
H	-2.067986000	1.665913000	3.462647000
H	1.339061000	2.485432000	2.993923000
H	2.326685000	4.601694000	3.905670000
H	-1.503148000	5.648176000	5.561192000
H	-6.066002000	5.028372000	6.596232000
H	-8.004387000	3.434564000	6.392748000

H	-5.677990000	0.688190000	3.985358000
H	-7.795209000	1.321176000	5.118113000
H	0.583452000	7.425362000	4.791272000
H	2.144489000	6.630840000	5.131559000
H	0.871795000	6.703188000	6.387457000
O	6.381649000	3.706245000	2.906374000
N	8.876077000	1.053366000	0.751297000
N	8.672671000	3.009638000	1.900971000
N	8.092699000	1.881193000	1.440585000
C	5.916572000	2.536482000	2.394164000
C	4.565908000	2.216593000	2.594507000
C	4.026633000	1.036010000	2.090586000
C	4.812523000	0.124696000	1.364298000
C	6.157045000	0.435533000	1.159912000
C	6.714012000	1.617915000	1.665069000
C	9.952244000	2.915013000	1.462900000
C	10.074211000	1.691738000	0.736850000
C	11.286964000	1.317406000	0.116311000
C	12.344758000	2.205467000	0.238265000
C	11.048909000	3.795226000	1.599294000
C	12.228662000	3.421956000	0.970103000
C	4.238504000	-1.172952000	0.857218000
H	7.366789000	3.737958000	2.701918000
H	3.959701000	2.918447000	3.170609000
H	2.972132000	0.802176000	2.258924000
H	6.801734000	-0.243890000	0.604255000
H	11.364804000	0.375498000	-0.431666000
H	13.302971000	1.969723000	-0.227301000
H	10.976575000	4.716098000	2.180091000
H	13.093796000	4.083111000	1.047334000
H	4.715351000	-2.021493000	1.373293000
H	3.154531000	-1.226068000	1.032634000
H	4.427007000	-1.299318000	-0.222892000
O	2.488341000	4.990562000	-5.994823000
N	3.890621000	7.188575000	-2.662473000
N	3.941802000	5.250038000	-3.858120000
N	3.458112000	6.502390000	-3.718968000
C	2.078130000	6.262289000	-5.746955000
C	1.160442000	6.845498000	-6.631919000
C	0.697156000	8.143804000	-6.431237000
C	1.129062000	8.913248000	-5.337145000
C	2.041256000	8.339521000	-4.451215000
C	2.519192000	7.035652000	-4.643387000
C	4.759112000	5.101710000	-2.786266000

C	4.719182000	6.315035000	-2.036387000
C	5.441138000	6.463452000	-0.830167000
C	6.177899000	5.365947000	-0.411101000
C	5.536917000	4.002742000	-2.357505000
C	6.227397000	4.157216000	-1.163053000
C	0.660132000	10.331466000	-5.144607000
H	3.167929000	4.763993000	-5.287169000
H	0.836180000	6.252036000	-7.489029000
H	-0.016239000	8.582654000	-7.133009000
H	2.402930000	8.900782000	-3.591192000
H	5.403475000	7.400289000	-0.271004000
H	6.749532000	5.423475000	0.516489000
H	5.601424000	3.081856000	-2.940227000
H	6.831223000	3.324939000	-0.796648000
H	1.507847000	11.026631000	-5.261090000
H	-0.110906000	10.601604000	-5.880686000
H	0.249002000	10.483765000	-4.131731000
O	-5.803860000	5.125768000	-0.773736000
N	-3.309431000	2.472888000	-2.928814000
N	-3.512836000	4.429160000	-1.779140000
N	-4.092810000	3.300715000	-2.239525000
C	-6.268936000	3.956005000	-1.285947000
C	-7.619600000	3.636115000	-1.085603000
C	-8.158870000	2.455529000	-1.589502000
C	-7.372986000	1.544219000	-2.315813000
C	-6.028463000	1.855055000	-2.520199000
C	-5.471496000	3.037438000	-2.015041000
C	-2.233264000	4.334535000	-2.217210000
C	-2.111297000	3.111261000	-2.943261000
C	-0.898544000	2.736929000	-3.563799000
C	0.159249000	3.624992000	-3.441849000
C	-1.136597000	5.214744000	-2.080809000
C	0.043153000	4.841481000	-2.710017000
C	-7.947008000	0.246563000	-2.822900000
H	-4.818720000	5.157481000	-0.978192000
H	-8.225806000	4.337970000	-0.509501000
H	-9.213391000	2.221698000	-1.421215000
H	-5.383775000	1.175633000	-3.075855000
H	-0.820704000	1.795020000	-4.111777000
H	1.117463000	3.389245000	-3.907411000
H	-1.208933000	6.135619000	-1.500020000
H	0.908222000	5.502654000	-2.632747000
H	-7.470144000	-0.601958000	-2.306832000
H	-9.030976000	0.193456000	-2.647473000

H	-7.758508000	0.120201000	-3.903003000
O	7.661348000	1.260747000	-2.274244000
N	10.155777000	-1.392132000	-4.429322000
N	9.952372000	0.564140000	-3.279648000
N	9.372401000	-0.564305000	-3.740033000
C	7.196271000	0.090986000	-2.786460000
C	5.845655000	-0.228865000	-2.586113000
C	5.306320000	-1.409456000	-3.090067000
C	6.092223000	-2.320803000	-3.816320000
C	7.436746000	-2.009965000	-4.020707000
C	7.993713000	-0.827582000	-3.515549000
C	11.231944000	0.469515000	-3.717717000
C	11.353911000	-0.753760000	-4.443769000
C	12.566665000	-1.128091000	-5.064307000
C	13.624459000	-0.240030000	-4.942353000
C	12.328610000	1.349728000	-3.581324000
C	13.508363000	0.976458000	-4.210515000
C	5.518204000	-3.618450000	-4.323400000
H	8.646488000	1.292461000	-2.478700000
H	5.239310000	0.472894000	-2.009964000
H	4.251750000	-1.643310000	-2.921653000
H	8.081433000	-2.689389000	-4.576363000
H	12.644506000	-2.070001000	-5.612285000
H	14.582673000	-0.475776000	-5.407919000
H	12.256275000	2.270599000	-3.000528000
H	14.373496000	1.637614000	-4.133284000
H	5.995053000	-4.466991000	-3.807326000
H	4.434232000	-3.671566000	-4.147984000
H	5.706707000	-3.744815000	-5.403510000
O	1.208647000	7.436026000	-0.814222000
N	2.610919000	9.634074000	2.518144000
N	2.662102000	7.695534000	1.322498000
N	2.178410000	8.947888000	1.461650000
C	0.798435000	8.707790000	-0.566341000
C	-0.119259000	9.290996000	-1.451301000
C	-0.582544000	10.589301000	-1.250619000
C	-0.150640000	11.358746000	-0.156527000
C	0.761555000	10.785018000	0.729403000
C	1.239490000	9.481150000	0.537232000
C	3.479411000	7.547207000	2.394351000
C	3.439481000	8.760534000	3.144231000
C	4.161437000	8.908950000	4.350451000
C	4.898197000	7.811445000	4.769517000
C	4.257218000	6.448240000	2.823114000

C	4.947695000	6.602715000	4.017566000
C	-0.619570000	12.776963000	0.036012000
H	1.888263000	7.209403000	-0.106539000
H	-0.443520000	8.697533000	-2.308411000
H	-1.295938000	11.028153000	-1.952391000
H	1.123228000	11.346281000	1.589425000
H	4.123773000	9.845787000	4.909613000
H	5.469833000	7.868973000	5.697108000
H	4.321724000	5.527355000	2.240391000
H	5.551522000	5.770436000	4.383970000
H	0.228146000	13.472130000	-0.080473000
H	-1.390606000	13.047102000	-0.700068000
H	-1.030700000	12.929264000	1.048886000
O	-7.083561000	7.571265000	4.406882000
N	-4.589132000	4.918386000	2.251805000
N	-4.792537000	6.874658000	3.401479000
N	-5.372509000	5.746214000	2.941092000
C	-7.548638000	6.401502000	3.894671000
C	-8.899301000	6.081613000	4.095015000
C	-9.438572000	4.901025000	3.591116000
C	-8.652688000	3.989719000	2.864802000
C	-7.308246000	4.300291000	2.660645000
C	-6.751210000	5.482963000	3.165530000
C	-3.512966000	6.780033000	2.963408000
C	-3.390986000	5.556739000	2.237391000
C	-2.178250000	5.182369000	1.616919000
C	-1.120461000	6.070506000	1.738742000
C	-2.416301000	7.660247000	3.099801000
C	-1.236548000	7.286977000	2.470610000
C	-9.226706000	2.692068000	2.357726000
H	-6.098422000	7.602979000	4.202426000
H	-9.505507000	6.783467000	4.671117000
H	-10.493093000	4.667196000	3.759403000
H	-6.663344000	3.621403000	2.104548000
H	-2.100391000	4.240529000	1.068734000
H	-0.162236000	5.834743000	1.273207000
H	-2.488635000	8.581118000	3.680598000
H	-0.371415000	7.948132000	2.547842000
H	-8.749858000	1.843527000	2.873800000
H	-10.310678000	2.638952000	2.533141000
H	-9.038203000	2.565702000	1.277617000

UVI (A.B)

S_0

O	4.860360000	-1.190269000	0.199186000
H	4.625042000	-0.280158000	0.483814000
N	2.144124000	-0.154831000	0.453526000
N	0.903830000	0.304105000	0.446486000
C	1.404394000	-2.462825000	0.498686000
H	0.400519000	-2.087983000	0.637869000
C	2.937636000	-4.265852000	0.200301000
H	3.138184000	-5.326400000	0.100122000
C	1.621556000	-3.826863000	0.381708000
C	3.773829000	-1.996360000	0.269290000
C	3.993582000	-3.369658000	0.148618000
H	5.007955000	-3.698167000	-0.060824000
C	2.452370000	-1.546752000	0.419048000
N	3.098774000	0.757995000	0.513710000
C	0.467961000	-4.795012000	0.434700000
H	-0.268343000	-4.571532000	-0.344260000
H	0.817622000	-5.818021000	0.287766000
H	-0.053260000	-4.769200000	1.396246000
C	1.053334000	1.639538000	0.532887000
C	0.564795000	3.954510000	0.742582000
H	-0.123756000	4.789547000	0.818989000
C	0.091536000	2.673760000	0.617402000
H	-0.969954000	2.458510000	0.603227000
C	2.434591000	1.925116000	0.571337000
C	1.962292000	4.235898000	0.779153000
H	2.265487000	5.268785000	0.883386000
C	2.906654000	3.246544000	0.694396000
H	3.968780000	3.449653000	0.738224000
I	-8.794712000	-2.385978000	-1.082459000
F	-7.152235000	-1.097147000	1.425964000
F	-6.227554000	-2.509263000	-3.042950000
C	-6.766639000	-1.833690000	-0.816689000
C	-6.311489000	-1.271750000	0.376107000
C	-5.843597000	-1.991855000	-1.850860000
O	-2.425582000	-1.797047000	6.960107000
H	-2.446926000	-2.148412000	6.020578000
N	-4.645417000	-2.821908000	5.383990000
N	-5.667218000	-3.270021000	4.647733000
C	-6.024136000	-2.625115000	7.379169000
H	-6.849062000	-3.038804000	6.800052000
C	-5.130864000	-1.714182000	9.412526000
H	-5.259331000	-1.414589000	10.455327000
C	-6.223726000	-2.253277000	8.708310000
C	-3.672367000	-1.950459000	7.475014000

C	-3.881069000	-1.574722000	8.811280000
H	-3.036686000	-1.135427000	9.348497000
C	-4.778961000	-2.464158000	6.751970000
N	-3.458974000	-2.747493000	4.761188000
C	-7.579877000	-2.391623000	9.352300000
H	-8.253196000	-1.582787000	9.017946000
H	-7.501020000	-2.339219000	10.448721000
H	-8.056050000	-3.349576000	9.091379000
C	-5.103114000	-3.528107000	3.439963000
C	-4.826405000	-4.226054000	1.179113000
H	-5.217089000	-4.638375000	0.246371000
C	-5.677600000	-4.051996000	2.260395000
H	-6.733842000	-4.322452000	2.213019000
C	-3.719086000	-3.194386000	3.507828000
C	-3.445506000	-3.885103000	1.246885000
H	-2.823341000	-4.043812000	0.365166000
C	-2.867758000	-3.365194000	2.395494000
H	-1.807286000	-3.115125000	2.449561000
I	-2.028497000	-0.494214000	-0.244162000
F	-3.670978000	-1.783052000	-2.752567000
F	-4.595084000	-0.371371000	1.717039000
C	-4.056574000	-1.046504000	-0.509926000
C	-4.511725000	-1.608442000	-1.702712000
C	-4.980415000	-0.888824000	0.524620000
O	-8.397630000	-1.083151000	-8.286710000
H	-8.376286000	-0.731787000	-7.347181000
N	-6.177795000	-0.058291000	-6.710593000
N	-5.155995000	0.389823000	-5.974336000
C	-4.799077000	-0.255084000	-8.705772000
H	-3.974151000	0.158605000	-8.126654000
C	-5.692349000	-1.166017000	-10.739129000
H	-5.564681000	-1.466088000	-11.781567000
C	-4.598912000	-0.627357000	-10.034220000
C	-7.150270000	-0.930175000	-8.800924000
C	-6.942144000	-1.305476000	-10.137883000
H	-7.785953000	-1.745207000	-10.674406000
C	-6.043676000	-0.416476000	-8.077880000
N	-7.363664000	-0.133142000	-6.087098000
C	-3.243335000	-0.488576000	-10.678901000
H	-2.570017000	-1.297412000	-10.344548000
H	-3.321618000	-0.541416000	-11.774630000
H	-2.766588000	0.468942000	-10.417290000
C	-5.719524000	0.647473000	-4.765874000
C	-5.997034000	1.344942000	-2.504660000

H	-5.605548000	1.757741000	-1.572281000
C	-5.145612000	1.171797000	-3.586997000
H	-4.088796000	1.441818000	-3.538928000
C	-7.104124000	0.314178000	-4.834428000
C	-7.378517000	1.004417000	-2.573132000
H	-8.000214000	1.162665000	-1.690750000
C	-7.954879000	0.484511000	-3.721210000
H	-9.015780000	0.234917000	-3.776139000
I	3.892028000	5.373815000	-2.459627000
F	1.463229000	7.531311000	-2.147943000
O	10.133040000	6.798022000	-0.041871000
H	10.110896000	6.446180000	-0.981036000
N	7.912405000	5.772683000	-1.617624000
F	1.937999000	2.809847000	-2.698096000
N	6.891404000	5.325049000	-2.354244000
C	6.533686000	5.969477000	0.377555000
H	5.709561000	5.556266000	-0.201925000
C	7.427758000	6.880888000	2.410548000
H	7.299291000	7.180480000	3.453350000
C	6.333521000	6.341751000	1.706000000
C	8.885680000	6.645046000	0.472345000
C	8.677554000	7.020348000	1.809303000
H	9.521363000	7.460079000	2.345827000
C	7.779086000	6.131347000	-0.250699000
N	9.099074000	5.848013000	-2.241482000
C	4.978748000	6.203444000	2.350323000
H	4.305424000	7.012283000	2.015973000
H	5.057609000	6.255848000	3.446744000
H	4.501974000	5.245942000	2.088694000
C	1.783375000	5.178679000	-2.433894000
C	7.454934000	5.067398000	-3.562706000
C	7.731643000	4.369450000	-5.823557000
H	7.340958000	3.957131000	-6.756299000
C	6.881022000	4.543074000	-4.741582000
H	5.824781000	4.272618000	-4.788958000
C	8.839534000	5.400693000	-3.494151000
C	9.113127000	4.709975000	-5.755086000
H	9.734824000	4.551728000	-6.637468000
C	9.690289000	5.230360000	-4.607370000
H	10.750390000	5.479476000	-4.552078000
C	0.947306000	6.282266000	-2.263047000
C	1.181573000	3.924911000	-2.554344000
I	-3.148231000	4.683166000	-2.315082000
F	-0.718633000	2.526148000	-2.627128000

F	-1.194203000	7.247133000	-2.076613000
C	-1.038779000	4.878780000	-2.341176000
C	-0.202710000	3.775193000	-2.512023000
C	-0.436977000	6.132548000	-2.220726000
O	0.706827000	-7.985859000	2.687376000
H	0.728168000	-7.634495000	3.626902000
N	2.926654000	-6.961012000	4.263486000
N	3.948455000	-6.512899000	4.999743000
C	4.305374000	-7.157809000	2.268308000
H	5.130299000	-6.744116000	2.847424000
C	3.412090000	-8.068718000	0.234971000
H	3.539783000	-8.368801000	-0.807489000
C	4.505527000	-7.530075000	0.939870000
C	1.954180000	-7.832901000	2.173156000
C	2.162302000	-8.208200000	0.836186000
H	1.318496000	-8.647925000	0.299671000
C	3.060773000	-7.319197000	2.896198000
N	1.740785000	-7.035863000	4.886982000
C	5.861113000	-7.391295000	0.295176000
H	6.534433000	-8.200134000	0.629530000
H	5.782831000	-7.444137000	-0.800552000
H	6.337859000	-6.433780000	0.556788000
C	3.384925000	-6.255248000	6.208205000
C	3.107416000	-5.557779000	8.469418000
H	3.498901000	-5.144981000	9.401798000
C	3.958837000	-5.730924000	7.387081000
H	5.015653000	-5.460903000	7.435151000
C	2.000324000	-6.588543000	6.139650000
C	1.725933000	-5.898304000	8.400948000
H	1.104235000	-5.740056000	9.283329000
C	1.149571000	-6.418209000	7.252869000
H	0.088669000	-6.667804000	7.197940000
I	-9.785526000	1.956610000	2.471050000
F	-8.143049000	3.245441000	4.979473000
F	-7.218368000	1.833325000	0.510559000
C	-7.757453000	2.508898000	2.736820000
C	-7.302303000	3.070838000	3.929615000
C	-6.834411000	2.350732000	1.702649000
I	-3.019314000	3.848367000	3.309365000
F	-4.661792000	2.559536000	0.800941000
O	3.745345000	2.973527000	3.846580000
H	3.428394000	3.917773000	3.967243000
N	1.046274000	4.049084000	3.989220000
F	-5.585898000	3.971216000	5.270548000

N	-0.192191000	4.550578000	4.030019000
C	0.235821000	1.755252000	3.946339000
H	-0.774469000	2.153988000	4.032023000
C	1.757525000	-0.088134000	3.725855000
H	1.943204000	-1.161412000	3.644492000
C	0.435131000	0.378775000	3.844846000
C	2.645772000	2.178886000	3.836020000
C	2.838300000	0.792111000	3.730788000
H	3.863997000	0.434660000	3.614983000
C	1.312550000	2.656384000	3.923017000
N	2.046414000	4.944024000	4.037094000
C	-0.735139000	-0.570000000	3.824882000
H	-1.226754000	-0.561870000	2.836131000
H	-0.409368000	-1.599795000	4.035709000
H	-1.493075000	-0.293303000	4.574298000
C	-5.047388000	3.296078000	3.043595000
C	0.007443000	5.889905000	4.129403000
C	-0.407696000	8.226129000	4.349590000
H	-1.085764000	9.075453000	4.450291000
C	-0.926990000	6.943615000	4.240772000
H	-2.001316000	6.752420000	4.258254000
C	1.411745000	6.138756000	4.127055000
C	0.995349000	8.472151000	4.340729000
H	1.346704000	9.500857000	4.430674000
C	1.925035000	7.449558000	4.226888000
H	2.998473000	7.638140000	4.231595000
C	-5.502538000	2.734139000	1.850799000
C	-5.971230000	3.453765000	4.078128000
O	-9.388445000	3.259436000	-4.733201000
H	-9.367100000	3.610800000	-3.793672000
N	-7.168609000	4.284296000	-3.157084000
N	-6.146809000	4.732410000	-2.420827000
C	-5.789891000	4.087504000	-5.152264000
H	-4.964965000	4.501193000	-4.573146000
C	-6.683163000	3.176571000	-7.185620000
H	-6.555495000	2.876499000	-8.228059000
C	-5.589726000	3.715230000	-6.480711000
C	-8.141084000	3.412413000	-5.247415000
C	-7.932958000	3.037111000	-6.584374000
H	-8.776767000	2.597380000	-7.120897000
C	-7.034490000	3.926112000	-4.524371000
N	-8.354477000	4.209445000	-2.533589000
C	-4.234149000	3.854012000	-7.125393000
H	-3.560831000	3.045176000	-6.791040000

H	-4.312432000	3.801171000	-8.221122000
H	-3.757402000	4.811529000	-6.863781000
C	-6.710338000	4.990060000	-1.212364000
C	-6.987847000	5.687529000	1.048848000
H	-6.596362000	6.100328000	1.981227000
C	-6.136426000	5.514385000	-0.033489000
H	-5.079610000	5.784406000	0.014580000
C	-8.094938000	4.656766000	-1.280919000
C	-8.369330000	5.347004000	0.980377000
H	-8.991028000	5.505253000	1.862759000
C	-8.945693000	4.827099000	-0.167701000
H	-10.006594000	4.577505000	-0.222631000
I	-7.803898000	-6.728565000	-4.635968000
F	-6.161421000	-5.439734000	-2.127544000
F	-5.236740000	-6.851851000	-6.596459000
C	-5.775825000	-6.176277000	-4.370198000
C	-5.320676000	-5.614335000	-3.177402000
C	-4.852783000	-6.334443000	-5.404369000
O	-1.434820000	-6.139586000	3.406581000
H	-1.456194000	-6.490977000	2.466898000
N	-3.654603000	-7.164495000	1.830481000
N	-4.676405000	-7.612609000	1.094224000
C	-5.033322000	-6.967702000	3.825661000
H	-5.858248000	-7.381391000	3.246544000
C	-4.140050000	-6.056770000	5.859017000
C	-5.232912000	-6.595864000	5.154802000
C	-2.681551000	-6.293057000	3.921509000
C	-2.890256000	-5.917310000	5.257771000
H	-2.045872000	-5.478015000	5.794988000
C	-3.788148000	-6.806746000	3.198462000
N	-2.468160000	-7.090079000	1.207678000
C	-4.112300000	-7.870695000	-0.113545000
C	-3.835591000	-8.568643000	-2.374396000
H	-4.226275000	-8.980963000	-3.307138000
C	-4.686786000	-8.394584000	-1.293113000
H	-5.743028000	-8.665040000	-1.340490000
C	-2.728275000	-7.536960000	-0.045685000
C	-2.454686000	-8.227668000	-2.306622000
H	-1.832409000	-8.386364000	-3.188308000
C	-1.876944000	-7.707733000	-1.158232000
H	-0.816547000	-7.457709000	-1.103895000
I	-1.037686000	-4.836808000	-3.797653000
F	-2.680164000	-6.125639000	-6.306076000
O	5.726972000	-5.711648000	-3.260437000

H	5.410019000	-4.767399000	-3.139775000
N	3.027902000	-4.636091000	-3.117797000
F	-3.604279000	-4.713963000	-1.836464000
N	1.789436000	-4.134597000	-3.076998000
C	2.217449000	-6.929923000	-3.160678000
H	1.207159000	-6.531187000	-3.074994000
C	3.739152000	-8.773310000	-3.381162000
H	3.924836000	-9.846586000	-3.462525000
C	2.416757000	-8.306403000	-3.262171000
C	4.627401000	-6.506289000	-3.270998000
C	4.819926000	-7.893061000	-3.376229000
H	5.845624000	-8.250515000	-3.492034000
C	3.294178000	-6.028791000	-3.184001000
N	4.028041000	-3.741151000	-3.069924000
C	1.246423000	-9.255167000	-3.282099000
H	0.754888000	-9.247040000	-4.270914000
H	1.572203000	-10.284981000	-3.071248000
H	0.488563000	-8.978490000	-2.532698000
C	-3.065760000	-5.389099000	-4.063422000
C	1.989072000	-2.795270000	-2.977615000
C	1.573932000	-0.459046000	-2.757428000
H	0.895863000	0.390277000	-2.656726000
C	1.054637000	-1.741559000	-2.866246000
H	-0.019689000	-1.932755000	-2.848763000
C	3.393373000	-2.546419000	-2.979962000
C	2.976977000	-0.213025000	-2.766288000
H	3.328331000	0.815686000	-2.676343000
C	3.906663000	-1.235616000	-2.880130000
H	4.980101000	-1.047036000	-2.875422000
C	-3.520910000	-5.951036000	-5.256218000
C	-3.989601000	-5.231409000	-3.028889000
I	2.901214000	9.716402000	1.093882000
F	0.472415000	11.873899000	1.405566000
F	0.947191000	7.152411000	0.855416000
C	0.792560000	9.521267000	1.119625000
C	-0.043508000	10.624853000	1.290462000
C	0.190761000	8.267500000	0.999167000
I	-4.139045000	9.025753000	1.238427000
F	-1.709447000	6.868735000	0.926381000
F	-2.185016000	11.589721000	1.476895000
C	-2.029592000	9.221367000	1.212332000
C	-1.193524000	8.117781000	1.041476000
C	-1.427791000	10.475135000	1.332783000
O	11.123854000	2.455435000	-3.595379000

H	11.101710000	2.103592000	-4.534545000
N	8.903219000	1.430096000	-5.171133000
N	7.882217000	0.982461000	-5.907753000
C	7.524500000	1.626889000	-3.175954000
H	6.700375000	1.213679000	-3.755434000
C	8.418572000	2.538300000	-1.142960000
H	8.290105000	2.837893000	-0.100159000
C	7.324338000	1.999164000	-1.847503000
C	9.876494000	2.302459000	-3.081164000
C	9.668368000	2.677760000	-1.744206000
H	10.512177000	3.117492000	-1.207682000
C	8.769900000	1.788759000	-3.804208000
N	10.089887000	1.505426000	-5.794990000
C	5.969550000	1.860852000	-1.203184000
H	5.296245000	2.669673000	-1.537523000
H	6.048403000	1.913273000	-0.106773000
H	5.492821000	0.903344000	-1.464802000
C	8.445747000	0.724811000	-7.116215000
C	8.722457000	0.026863000	-9.377066000
H	8.331772000	-0.385456000	-10.309808000
C	7.871836000	0.200486000	-8.295091000
H	6.815594000	-0.069970000	-8.342467000
C	9.830348000	1.058105000	-7.047660000
C	10.103941000	0.367387000	-9.308595000
H	10.725638000	0.209140000	-10.190976000
C	10.681103000	0.887772000	-8.160879000
H	11.741204000	1.136888000	-8.105587000
O	-0.283995000	-3.643285000	6.240878000
H	-0.262651000	-3.291921000	7.180407000
N	1.935840000	-2.618425000	7.816995000
N	2.957641000	-2.170312000	8.553252000
C	3.314559000	-2.815218000	5.821816000
H	4.139485000	-2.401528000	6.400933000
C	2.421300000	-3.726072000	3.788438000
H	2.548862000	-4.026324000	2.746027000
C	3.514721000	-3.187479000	4.493362000
C	0.963365000	-3.490309000	5.726664000
C	1.171498000	-3.865630000	4.389713000
H	0.327683000	-4.305342000	3.853182000
C	2.069959000	-2.976609000	6.449707000
N	0.749971000	-2.693276000	8.440490000
C	4.870300000	-3.048709000	3.848686000
H	5.543619000	-3.857546000	4.183039000
H	4.792018000	-3.101549000	2.752957000

H	5.347047000	-2.091189000	4.110300000
C	2.394111000	-1.912661000	9.761714000
C	2.116603000	-1.215192000	12.022927000
H	2.508087000	-0.802393000	12.955307000
C	2.968023000	-1.388336000	10.940590000
H	4.024839000	-1.118315000	10.988660000
C	1.009511000	-2.245955000	9.693159000
C	0.735119000	-1.555716000	11.954456000
H	0.113421000	-1.397468000	12.836837000
C	0.158757000	-2.075622000	10.806378000
H	-0.902145000	-2.325216000	10.751449000
O	6.103698000	0.181205000	2.352423000
H	6.420648000	-0.763038000	2.231765000
N	8.802771000	-0.894346000	2.209788000
N	10.041236000	-1.395840000	2.168989000
C	9.613224000	1.399485000	2.252669000
H	10.623514000	1.000749000	2.166985000
C	8.091521000	3.242872000	2.473152000
H	7.905842000	4.316150000	2.554515000
C	9.413914000	2.775962000	2.354161000
C	7.203844000	0.975421000	2.363685000
C	7.010745000	2.362627000	2.468218000
H	5.985049000	2.720078000	2.584024000
C	8.536496000	0.498353000	2.275993000
N	7.802632000	-1.789286000	2.161915000
C	10.584250000	3.724730000	2.374090000
H	11.074986000	3.716125000	3.363266000
H	10.258471000	4.754542000	2.163239000
H	11.342121000	3.448041000	1.624710000
C	9.841377000	-2.736082000	2.070660000
C	10.256742000	-5.071391000	1.849418000
H	10.934810000	-5.920715000	1.748716000
C	10.775236000	-3.789356000	1.958598000
H	11.849562000	-3.598160000	1.941116000
C	8.437301000	-2.984019000	2.071953000
C	8.853696000	-5.317413000	1.858279000
H	8.502341000	-6.346119000	1.768334000
C	7.924584000	-4.295256000	1.972813000
H	6.850572000	-4.483402000	1.967413000
O	7.094517000	-4.161364000	-1.201101000
H	7.411480000	-5.105623000	-1.321737000
N	9.793585000	-5.236934000	-1.343721000
N	11.032050000	-5.738428000	-1.384520000
C	10.604037000	-2.943103000	-1.300840000

H	11.614328000	-3.341839000	-1.386524000
C	9.082335000	-1.099716000	-1.080357000
H	8.896656000	-0.026438000	-0.998993000
C	10.404728000	-1.566626000	-1.199347000
C	8.194664000	-3.367176000	-1.189813000
C	8.001541000	-1.980005000	-1.085305000
H	6.975887000	-1.622443000	-0.969462000
C	9.527310000	-3.844234000	-1.277515000
N	8.793446000	-6.131874000	-1.391594000
C	11.575064000	-0.617858000	-1.179420000
H	12.065800000	-0.626463000	-0.190243000
H	11.249285000	0.411956000	-1.390270000
H	12.332935000	-0.894547000	-1.928799000
C	10.832190000	-7.078670000	-1.482849000
C	11.247556000	-9.413979000	-1.704091000
H	11.925624000	-10.263302000	-1.804792000
C	11.766050000	-8.131944000	-1.594911000
H	12.840375000	-7.940748000	-1.612393000
C	9.428115000	-7.326607000	-1.481556000
C	9.844509000	-9.660000000	-1.695230000
H	9.493155000	-10.688707000	-1.785175000
C	8.915398000	-8.637844000	-1.580696000
H	7.841386000	-8.825990000	-1.586096000
C	-6.603355000	-6.724291000	5.845447000
H	-7.315214000	-6.102581000	5.343815000
H	-6.928757000	-7.742834000	5.805649000
H	-6.518286000	-6.416689000	6.866743000
H	-4.271070000	-5.748149000	6.875130000

S_1 (enol)

O	4.786454000	-1.175345000	0.245033000
H	4.444671000	-0.224075000	0.417652000
N	2.133220000	-0.195421000	0.425916000
N	0.862957000	0.307885000	0.457614000
C	1.376741000	-2.474190000	0.518068000
H	0.364936000	-2.123786000	0.655907000
C	2.967166000	-4.289435000	0.205379000
H	3.161037000	-5.349460000	0.104499000
C	1.637109000	-3.840521000	0.387610000
C	3.765245000	-2.009345000	0.282633000
C	4.013602000	-3.386951000	0.156901000
H	5.033660000	-3.695191000	-0.051974000
C	2.399324000	-1.533221000	0.429074000
N	3.133950000	0.742633000	0.473929000

C	0.500216000	-4.813661000	0.416399000
H	-0.165458000	-4.641767000	-0.438020000
H	0.855411000	-5.843045000	0.379813000
H	-0.107245000	-4.698941000	1.320001000
C	1.061528000	1.631062000	0.548216000
C	0.609442000	3.969572000	0.754032000
H	-0.074829000	4.808277000	0.830747000
C	0.123182000	2.680588000	0.639709000
H	-0.940262000	2.474012000	0.638526000
C	2.468877000	1.906906000	0.565254000
C	1.998610000	4.233927000	0.776908000
H	2.317887000	5.263352000	0.871967000
C	2.939874000	3.222036000	0.684850000
H	4.003328000	3.420393000	0.718172000
I	-8.795042000	-2.385290000	-1.082485000
F	-7.152530000	-1.096345000	1.425857000
F	-6.227876000	-2.508818000	-3.042950000
C	-6.766949000	-1.833064000	-0.816741000
C	-6.311783000	-1.271056000	0.376017000
C	-5.843906000	-1.991340000	-1.850895000
O	-2.425940000	-1.796037000	6.960080000
H	-2.447293000	-2.147468000	6.020576000
N	-4.645806000	-2.820922000	5.384022000
N	-5.667619000	-3.269047000	4.647791000
C	-6.024529000	-2.623931000	7.379179000
H	-6.849468000	-3.037629000	6.800086000
C	-5.131235000	-1.712889000	9.412475000
H	-5.259697000	-1.413216000	10.455255000
C	-6.224114000	-2.251990000	8.708292000
C	-3.672735000	-1.949362000	7.474990000
C	-3.881431000	-1.573521000	8.811228000
H	-3.037032000	-1.134221000	9.348418000
C	-4.779344000	-2.463069000	6.751976000
N	-3.459356000	-2.746599000	4.761223000
C	-7.580274000	-2.390236000	9.352282000
H	-8.253559000	-1.581397000	9.017867000
H	-7.501422000	-2.337757000	10.448700000
H	-8.056483000	-3.348189000	9.091427000
C	-5.103519000	-3.527242000	3.440043000
C	-4.826822000	-4.225362000	1.179244000
H	-5.217517000	-4.637734000	0.246530000
C	-5.678017000	-4.051193000	2.260509000
H	-6.734270000	-4.321610000	2.213145000
C	-3.719466000	-3.193626000	3.507911000

C	-3.445903000	-3.884495000	1.247012000
H	-2.823746000	-4.043257000	0.365298000
C	-2.868176000	-3.364337000	2.395404000
H	-1.807656000	-3.114507000	2.449638000
I	-2.028717000	-0.493745000	-0.244222000
F	-3.671273000	-1.782687000	-2.752603000
F	-4.595352000	-0.370650000	1.716896000
C	-4.056853000	-1.045982000	-0.509989000
C	-4.512019000	-1.607975000	-1.702763000
C	-4.980697000	-0.888164000	0.524510000
O	-8.397862000	-1.082996000	-8.286826000
H	-8.376511000	-0.731564000	-7.347323000
N	-6.177997000	-0.058111000	-6.710769000
N	-5.156183000	0.390015000	-5.974537000
C	-4.799273000	-0.255101000	-8.705925000
H	-3.974335000	0.158597000	-8.126831000
C	-5.692568000	-1.166143000	-10.739222000
H	-5.564906000	-1.466296000	-11.781638000
C	-4.599114000	-0.627478000	-10.034345000
C	-7.150493000	-0.930106000	-8.801044000
C	-6.942373000	-1.305511000	-10.137974000
H	-7.786196000	-1.745247000	-10.674471000
C	-6.043883000	-0.416398000	-8.078029000
N	-7.363873000	-0.132871000	-6.087276000
C	-3.243528000	-0.488796000	-10.679027000
H	-2.570245000	-1.297635000	-10.344612000
H	-3.321806000	-0.541712000	-11.774753000
H	-2.766745000	0.468721000	-10.417481000
C	-5.719710000	0.647775000	-4.766097000
C	-5.997206000	1.345416000	-2.504935000
H	-5.605710000	1.758267000	-1.572583000
C	-5.145785000	1.172161000	-3.587255000
H	-4.088958000	1.442143000	-3.539198000
C	-7.104323000	0.314530000	-4.834636000
C	-7.378703000	1.004941000	-2.573391000
H	-8.000399000	1.163277000	-1.691025000
C	-7.955078000	0.484976000	-3.721436000
H	-9.015989000	0.235420000	-3.776354000
I	3.892015000	5.373900000	-2.460127000
F	1.463299000	7.531515000	-2.148613000
O	10.133068000	6.798034000	-0.042433000
H	10.110916000	6.446125000	-0.981573000
N	7.912402000	5.772670000	-1.618127000
F	1.937883000	2.809992000	-2.698419000

N	6.891389000	5.325023000	-2.354722000
C	6.533679000	5.969661000	0.377029000
H	5.709540000	5.556441000	-0.202426000
C	7.427774000	6.881183000	2.409963000
H	7.299312000	7.180854000	3.452742000
C	6.333519000	6.342039000	1.705446000
C	8.885698000	6.645144000	0.471786000
C	8.677578000	7.020549000	1.808715000
H	9.521401000	7.460286000	2.345213000
C	7.779089000	6.131437000	-0.251229000
N	9.099078000	5.847909000	-2.241982000
C	4.978750000	6.203828000	2.349761000
H	4.305449000	7.012673000	2.015357000
H	5.057595000	6.256310000	3.446188000
H	4.501889000	5.246301000	2.088228000
C	1.783353000	5.178850000	-2.434394000
C	7.454916000	5.067264000	-3.563162000
C	7.731612000	4.369143000	-5.823961000
H	7.340916000	3.956772000	-6.756675000
C	6.880991000	4.542877000	-4.742003000
H	5.824739000	4.272460000	-4.789367000
C	8.839528000	5.400508000	-3.494622000
C	9.113108000	4.709618000	-5.755505000
H	9.734805000	4.551282000	-6.637871000
C	9.690284000	5.230062000	-4.607823000
H	10.750395000	5.479140000	-4.552542000
C	0.947326000	6.282483000	-2.263631000
C	1.181503000	3.925097000	-2.554758000
I	-3.148273000	4.683541000	-2.315578000
F	-0.718758000	2.526404000	-2.627454000
F	-1.194145000	7.247448000	-2.077280000
C	-1.038813000	4.879069000	-2.341673000
C	-0.202787000	3.775438000	-2.512435000
C	-0.436962000	6.132822000	-2.221308000
O	0.706251000	-7.985279000	2.687812000
H	0.727600000	-7.633848000	3.627313000
N	2.926109000	-6.960407000	4.263863000
N	3.947922000	-6.512281000	5.000095000
C	4.304833000	-7.157401000	2.268708000
H	5.129770000	-6.743699000	2.847800000
C	3.411527000	-8.068421000	0.235430000
H	3.539215000	-8.368583000	-0.807007000
C	4.504981000	-7.529770000	0.940298000
C	1.953614000	-7.832406000	2.173589000

C	2.161729000	-8.207810000	0.836648000
H	1.317909000	-8.647540000	0.300159000
C	3.060222000	-7.318694000	2.896602000
N	1.740233000	-7.035166000	4.887357000
C	5.860576000	-7.391089000	0.295603000
H	6.533861000	-8.199932000	0.630019000
H	5.782299000	-7.444007000	-0.800122000
H	6.337368000	-6.433572000	0.557157000
C	3.384396000	-6.254521000	6.208535000
C	3.106899000	-5.556879000	8.469696000
H	3.498395000	-5.144030000	9.402049000
C	3.958321000	-5.730135000	7.387377000
H	5.015146000	-5.460152000	7.435434000
C	1.999781000	-6.587766000	6.139995000
C	1.725402000	-5.897354000	8.401241000
H	1.103706000	-5.739018000	9.283606000
C	1.149028000	-6.417319000	7.253196000
H	0.088117000	-6.666875000	7.198278000
I	-9.785707000	1.957591000	2.470707000
F	-8.143195000	3.246536000	4.979048000
F	-7.218540000	1.834064000	0.510241000
C	-7.757613000	2.509818000	2.736450000
C	-7.302448000	3.071825000	3.929208000
C	-6.834571000	2.351541000	1.702297000
I	-3.019425000	3.849139000	3.308929000
F	-4.661938000	2.560194000	0.800588000
O	3.745196000	2.974070000	3.846251000
H	3.428282000	3.918348000	3.966852000
N	1.046167000	4.049744000	3.988797000
F	-5.586016000	3.972231000	5.270088000
N	-0.192279000	4.551290000	4.029552000
C	0.235623000	1.755942000	3.946075000
H	-0.774651000	2.154724000	4.031724000
C	1.757255000	-0.087521000	3.725733000
H	1.942891000	-1.160812000	3.644448000
C	0.434880000	0.379450000	3.844682000
C	2.645592000	2.179472000	3.835741000
C	2.838065000	0.792682000	3.730610000
H	3.863749000	0.435182000	3.614837000
C	1.312388000	2.657030000	3.922695000
N	2.046341000	4.944649000	4.036613000
C	-0.735468000	-0.569279000	3.824806000
H	-1.227104000	-0.561171000	2.836054000
H	-0.409777000	-1.599063000	4.035740000

H	-1.493359000	-0.292500000	4.574169000
C	-5.047518000	3.296913000	3.043186000
C	0.007408000	5.890617000	4.128841000
C	-0.407640000	8.226873000	4.348858000
H	-1.085675000	9.076230000	4.449494000
C	-0.926985000	6.944371000	4.240128000
H	-2.001318000	6.753221000	4.257617000
C	1.411719000	6.139411000	4.126484000
C	0.995415000	8.472839000	4.339988000
H	1.346811000	9.501537000	4.429862000
C	1.925061000	7.450201000	4.226227000
H	2.998507000	7.638740000	4.230927000
C	-5.502684000	2.734905000	1.850428000
C	-5.971362000	3.454710000	4.077702000
O	-9.388527000	3.259886000	-4.733635000
H	-9.367175000	3.611316000	-3.794131000
N	-7.168661000	4.284771000	-3.157577000
N	-6.146848000	4.732896000	-2.421346000
C	-5.789938000	4.087781000	-5.152734000
H	-4.964999000	4.501478000	-4.573640000
C	-6.683233000	3.176738000	-7.186030000
H	-6.555571000	2.876586000	-8.228447000
C	-5.589779000	3.715404000	-6.481153000
C	-8.141157000	3.412775000	-5.247852000
C	-7.933038000	3.037370000	-6.584783000
H	-8.776860000	2.597635000	-7.121280000
C	-7.034548000	3.926483000	-4.524838000
N	-8.354536000	4.210012000	-2.534084000
C	-4.234193000	3.854085000	-7.125836000
H	-3.560908000	3.045247000	-6.791421000
H	-4.312471000	3.801170000	-8.221562000
H	-3.757410000	4.811602000	-6.864290000
C	-6.710375000	4.990656000	-1.212905000
C	-6.987870000	5.688298000	1.048256000
H	-6.596375000	6.101148000	1.980608000
C	-6.136450000	5.515042000	-0.034063000
H	-5.079623000	5.785024000	0.013993000
C	-8.094987000	4.657411000	-1.281445000
C	-8.369366000	5.347822000	0.979800000
H	-8.991064000	5.506159000	1.862167000
C	-8.945743000	4.827858000	-0.168245000
H	-10.006654000	4.578301000	-0.223163000
I	-7.804378000	-6.728171000	-4.635676000
F	-6.161865000	-5.439226000	-2.127334000

F	-5.237211000	-6.851699000	-6.596142000
C	-5.776284000	-6.175945000	-4.369933000
C	-5.321120000	-5.613936000	-3.177174000
C	-4.853241000	-6.334222000	-5.404087000
O	-1.435325000	-6.138864000	3.406863000
H	-1.456673000	-6.490279000	2.467172000
N	-3.655141000	-7.163803000	1.830831000
N	-4.676956000	-7.611929000	1.094599000
C	-5.033865000	-6.966813000	3.825987000
H	-5.858803000	-7.380510000	3.246894000
C	-4.140570000	-6.055770000	5.859284000
C	-5.233449000	-6.594871000	5.155100000
C	-2.682067000	-6.292256000	3.921803000
C	-2.890767000	-5.916402000	5.258036000
H	-2.046368000	-5.477102000	5.795227000
C	-3.788680000	-6.805950000	3.198785000
N	-2.468692000	-7.089479000	1.208030000
C	-4.112854000	-7.870123000	-0.113148000
C	-3.836158000	-8.568244000	-2.373947000
H	-4.226852000	-8.980616000	-3.306662000
C	-4.687352000	-8.394074000	-1.292682000
H	-5.743605000	-8.664492000	-1.340046000
C	-2.728816000	-7.536439000	-0.045303000
C	-2.455239000	-8.227320000	-2.306189000
H	-1.832963000	-8.386103000	-3.187859000
C	-1.877485000	-7.707325000	-1.157832000
H	-0.817078000	-7.457340000	-1.103507000
I	-1.038096000	-4.836623000	-3.797453000
F	-2.680609000	-6.125568000	-6.305795000
O	5.726524000	-5.711692000	-3.260132000
H	5.409607000	-4.767422000	-3.139539000
N	3.027496000	-4.636018000	-3.117586000
F	-3.604696000	-4.713535000	-1.836290000
N	1.789050000	-4.134472000	-3.076831000
C	2.216952000	-6.929821000	-3.160308000
H	1.206678000	-6.531040000	-3.074659000
C	3.738583000	-8.773284000	-3.380650000
H	3.924225000	-9.846573000	-3.461935000
C	2.416206000	-8.306316000	-3.261701000
C	4.626922000	-6.506291000	-3.270642000
C	4.819392000	-7.893078000	-3.375773000
H	5.845077000	-8.250581000	-3.491546000
C	3.293717000	-6.028734000	-3.183688000
N	4.027670000	-3.741114000	-3.069770000

C	1.245835000	-9.255035000	-3.281568000
H	0.754306000	-9.246960000	-4.270387000
H	1.571572000	-10.284847000	-3.070642000
H	0.487981000	-8.978274000	-2.532192000
C	-3.066190000	-5.388852000	-4.063195000
C	1.988737000	-2.795146000	-2.977542000
C	1.573689000	-0.458890000	-2.757525000
H	0.895653000	0.390468000	-2.656888000
C	1.054344000	-1.741390000	-2.866254000
H	-0.019989000	-1.932542000	-2.848765000
C	3.393048000	-2.546351000	-2.979899000
C	2.976751000	-0.212926000	-2.766405000
H	3.328144000	0.815750000	-2.676528000
C	3.906391000	-1.235564000	-2.880153000
H	4.979873000	-1.047032000	-2.875455000
C	-3.521354000	-5.950857000	-5.255955000
C	-3.990031000	-5.231051000	-3.028680000
I	2.901350000	9.716782000	1.093065000
F	0.472635000	11.874397000	1.404579000
F	0.947228000	7.152817000	0.854777000
C	0.792688000	9.521733000	1.118792000
C	-0.043337000	10.625364000	1.289560000
C	0.190846000	8.267985000	0.998431000
I	-4.138937000	9.026422000	1.237614000
F	-1.709423000	6.869285000	0.925738000
F	-2.184807000	11.590330000	1.475911000
C	-2.029477000	9.221950000	1.211519000
C	-1.193451000	8.118319000	1.040754000
C	-1.427627000	10.475704000	1.331883000
O	11.123733000	2.455153000	-3.595624000
H	11.101581000	2.103244000	-4.534765000
N	8.903067000	1.429788000	-5.171319000
N	7.882052000	0.982142000	-5.907913000
C	7.524343000	1.626779000	-3.176162000
H	6.700205000	1.213560000	-3.755618000
C	8.418438000	2.538300000	-1.143228000
H	8.289977000	2.837973000	-0.100449000
C	7.324185000	1.999159000	-1.847742000
C	9.876363000	2.302263000	-3.081406000
C	9.668243000	2.677668000	-1.744476000
H	10.512066000	3.117405000	-1.207978000
C	8.769753000	1.788555000	-3.804420000
N	10.089742000	1.505027000	-5.795174000
C	5.969361000	1.860958000	-1.203414000

H	5.296118000	2.669769000	-1.537820000
H	6.048227000	1.913446000	-0.107011000
H	5.492635000	0.903388000	-1.464932000
C	8.445580000	0.724382000	-7.116353000
C	8.722276000	0.026261000	-9.377152000
H	8.331581000	-0.386109000	-10.309867000
C	7.871655000	0.199996000	-8.295195000
H	6.815403000	-0.070422000	-8.342558000
C	9.830193000	1.057627000	-7.047813000
C	10.103773000	0.366736000	-9.308696000
H	10.725469000	0.208401000	-10.191063000
C	10.680949000	0.887180000	-8.161014000
H	11.741058000	1.136258000	-8.105733000
O	-0.284422000	-3.642410000	6.240997000
H	-0.263069000	-3.290981000	7.180501000
N	1.935444000	-2.617525000	7.817055000
N	2.957258000	-2.169400000	8.553286000
C	3.314167000	-2.814516000	5.821898000
H	4.139106000	-2.400817000	6.400991000
C	2.420762000	-3.725788000	3.788656000
H	2.548803000	-4.025370000	2.746162000
C	3.514340000	-3.186943000	4.493494000
C	0.962950000	-3.489526000	5.726782000
C	1.171050000	-3.864879000	4.389841000
H	0.327258000	-4.304641000	3.853362000
C	2.069557000	-2.975813000	6.449794000
N	0.749569000	-2.692285000	8.440548000
C	4.869916000	-3.048212000	3.848796000
H	5.543197000	-3.857050000	4.183210000
H	4.791635000	-3.101126000	2.753069000
H	5.346692000	-2.090692000	4.110341000
C	2.393731000	-1.911640000	9.761726000
C	2.116235000	-1.213998000	12.022887000
H	2.507730000	-0.801148000	12.955240000
C	2.967656000	-1.387253000	10.940568000
H	4.024482000	-1.117271000	10.988625000
C	1.009118000	-2.244884000	9.693186000
C	0.734738000	-1.554473000	11.954432000
H	0.113041000	-1.396137000	12.836798000
C	0.158363000	-2.074438000	10.806387000
H	-0.902548000	-2.323994000	10.751469000
O	6.103392000	0.181478000	2.352222000
H	6.420296000	-0.762752000	2.231655000
N	8.802479000	-0.894120000	2.209768000

N	10.040925000	-1.395666000	2.169013000
C	9.613023000	1.399681000	2.252490000
H	10.623298000	1.000899000	2.166841000
C	8.091391000	3.243145000	2.472832000
H	7.905754000	4.316436000	2.554117000
C	9.413767000	2.776173000	2.353883000
C	7.203634000	0.975709000	2.363505000
C	7.010581000	2.362941000	2.467958000
H	5.984897000	2.720442000	2.583728000
C	8.536259000	0.498594000	2.275877000
N	7.802305000	-1.789025000	2.161953000
C	10.584140000	3.724896000	2.373751000
H	11.074869000	3.716343000	3.362931000
H	10.258403000	4.754707000	2.162824000
H	11.342005000	3.448124000	1.624396000
C	9.841013000	-2.735908000	2.070779000
C	10.256286000	-5.071249000	1.849707000
H	10.934321000	-5.920607000	1.749070000
C	10.774831000	-3.789227000	1.958798000
H	11.849165000	-3.598075000	1.941309000
C	8.436927000	-2.983788000	2.072080000
C	8.853232000	-5.317215000	1.858576000
H	8.501836000	-6.345913000	1.768703000
C	7.924159000	-4.295013000	1.973031000
H	6.850140000	-4.483116000	1.967637000
O	7.094140000	-4.161369000	-1.200768000
H	7.411008000	-5.105602000	-1.321509000
N	9.793144000	-5.237001000	-1.343423000
N	11.031589000	-5.738548000	-1.384179000
C	10.603687000	-2.943200000	-1.300702000
H	11.613963000	-3.341982000	-1.386351000
C	9.082055000	-1.099735000	-1.080374000
H	8.896419000	-0.026445000	-0.999075000
C	10.404432000	-1.566708000	-1.199309000
C	8.194277000	-3.367142000	-1.189705000
C	8.001191000	-1.980075000	-1.085280000
H	6.975613000	-1.622249000	-0.969386000
C	9.526923000	-3.844286000	-1.277337000
N	8.792969000	-6.131906000	-1.391238000
C	11.574805000	-0.617985000	-1.179442000
H	12.065534000	-0.626538000	-0.190261000
H	11.249068000	0.411826000	-1.390368000
H	12.332669000	-0.894758000	-1.928796000
C	10.831677000	-7.078790000	-1.482412000

C	11.246951000	-9.414131000	-1.703485000
H	11.924986000	-10.263488000	-1.804121000
C	11.765495000	-8.132108000	-1.594393000
H	12.839828000	-7.940956000	-1.611882000
C	9.427592000	-7.326671000	-1.481111000
C	9.843894000	-9.660096000	-1.694615000
H	9.492501000	-10.688795000	-1.784488000
C	8.914824000	-8.637894000	-1.580160000
H	7.840804000	-8.825997000	-1.585554000
C	-6.603902000	-6.723194000	5.845747000
H	-7.315732000	-6.101493000	5.344065000
H	-6.929343000	-7.741727000	5.806019000
H	-6.518826000	-6.415522000	6.867021000
H	-4.271584000	-5.747071000	6.875374000

S_1 (keto)

O	4.864996000	-1.188732000	-0.091735000
H	4.231013000	0.466594000	0.725176000
N	2.317279000	-0.171059000	0.372516000
N	1.088504000	0.341315000	0.311017000
C	1.545391000	-2.436159000	0.603578000
H	0.557274000	-2.045629000	0.811808000
C	3.014967000	-4.315684000	0.250688000
H	3.187205000	-5.380362000	0.156007000
C	1.731709000	-3.847783000	0.457559000
C	3.918470000	-2.027906000	0.146218000
C	4.097993000	-3.430498000	0.130029000
H	5.088185000	-3.788337000	-0.131707000
C	2.578754000	-1.574206000	0.412663000
N	3.244332000	0.763855000	0.716877000
C	0.535995000	-4.748645000	0.488904000
H	-0.138952000	-4.520339000	-0.346095000
H	0.831831000	-5.795289000	0.415263000
H	-0.046348000	-4.631245000	1.409239000
C	1.246463000	1.663800000	0.488365000
C	0.727118000	3.980204000	0.716245000
H	0.024806000	4.806640000	0.748406000
C	0.279479000	2.691510000	0.516436000
H	-0.771632000	2.464554000	0.385686000
C	2.616454000	1.962900000	0.702607000
C	2.101621000	4.267569000	0.863964000
H	2.392137000	5.301959000	0.985280000
C	3.065424000	3.271420000	0.865297000
H	4.117165000	3.480007000	0.997524000

I	-8.802268000	-2.383661000	-1.080612000
F	-7.158909000	-1.094849000	1.427244000
F	-6.235477000	-2.508339000	-3.041494000
C	-6.773950000	-1.832053000	-0.815302000
C	-6.318398000	-1.270002000	0.377288000
C	-5.851135000	-1.990795000	-1.849588000
O	-2.431603000	-1.795193000	6.960773000
H	-2.453231000	-2.146768000	6.021329000
N	-4.652072000	-2.819608000	5.385258000
N	-5.674156000	-3.267518000	4.649273000
C	-6.030390000	-2.621848000	7.380618000
H	-6.855563000	-3.035370000	6.801732000
C	-5.136453000	-1.710771000	9.413616000
H	-5.264640000	-1.410889000	10.456370000
C	-6.229628000	-2.249629000	8.709705000
C	-3.678360000	-1.948030000	7.475920000
C	-3.886706000	-1.571907000	8.812133000
H	-3.042073000	-1.132795000	9.349109000
C	-4.785260000	-2.461492000	6.753177000
N	-3.465704000	-2.745771000	4.762245000
C	-7.585723000	-2.387330000	9.353949000
H	-8.258801000	-1.578326000	9.019519000
H	-7.506667000	-2.334701000	10.450345000
H	-8.062288000	-3.345170000	9.093328000
C	-5.110346000	-3.526090000	3.441470000
C	-4.834262000	-4.224662000	1.180736000
H	-5.225250000	-4.637057000	0.248155000
C	-5.685216000	-4.050043000	2.262118000
H	-6.741565000	-4.320124000	2.214977000
C	-3.726172000	-3.192916000	3.509049000
C	-3.453220000	-3.884234000	1.248214000
H	-2.831265000	-4.043340000	0.366419000
C	-2.875128000	-3.364083000	2.396432000
H	-1.814518000	-3.114581000	2.450441000
I	-2.035098000	-0.494181000	-0.243769000
F	-3.678588000	-1.782994000	-2.751700000
F	-4.601445000	-0.369940000	1.717731000
C	-4.063541000	-1.045818000	-0.509146000
C	-4.519095000	-1.607853000	-1.701739000
C	-4.987165000	-0.887503000	0.525488000
O	-8.405892000	-1.082650000	-8.285229000
H	-8.384267000	-0.731075000	-7.345786000
N	-6.185425000	-0.058236000	-6.709715000
N	-5.163340000	0.389675000	-5.973729000

C	-4.807105000	-0.255994000	-8.705074000
H	-3.981934000	0.157528000	-8.126187000
C	-5.701044000	-1.167071000	-10.738073000
H	-5.573657000	-1.467433000	-11.780462000
C	-4.607294000	-0.628649000	-10.033468000
C	-7.158561000	-0.930249000	-8.799684000
C	-6.950791000	-1.305936000	-10.136589000
H	-7.794849000	-1.745483000	-10.672872000
C	-6.051661000	-0.416786000	-8.076940000
N	-7.371219000	-0.132510000	-6.086008000
C	-3.251773000	-0.490512000	-10.678404000
H	-2.578696000	-1.299517000	-10.343974000
H	-3.330255000	-0.543578000	-11.774108000
H	-2.774634000	0.466891000	-10.417092000
C	-5.726577000	0.647812000	-4.765234000
C	-6.003460000	1.345906000	-2.504136000
H	-5.611671000	1.758779000	-1.571917000
C	-5.152280000	1.172200000	-3.586574000
H	-4.095357000	1.441846000	-3.538740000
C	-7.111310000	0.315007000	-4.833484000
C	-7.385079000	1.005870000	-2.572302000
H	-8.006573000	1.164550000	-1.689856000
C	-7.961819000	0.485909000	-3.720166000
H	-9.022821000	0.236689000	-3.774863000
I	3.887080000	5.371177000	-2.461660000
F	1.459119000	7.529632000	-2.150078000
O	10.129008000	6.793666000	-0.045258000
H	10.106581000	6.441613000	-0.984337000
N	7.907740000	5.768772000	-1.620409000
F	1.932072000	2.807867000	-2.699208000
N	6.886456000	5.321340000	-2.356758000
C	6.529421000	5.966532000	0.374951000
H	5.705049000	5.553488000	-0.204298000
C	7.424160000	6.878089000	2.407586000
H	7.295973000	7.177968000	3.450339000
C	6.329615000	6.339181000	1.703354000
C	8.881676000	6.641264000	0.469198000
C	8.673906000	7.016951000	1.806103000
H	9.517964000	7.456499000	2.342386000
C	7.774777000	6.127802000	-0.253546000
N	9.094334000	5.843525000	-2.244478000
C	4.974664000	6.201672000	2.347798000
H	4.301826000	7.010533000	2.013460000
H	5.053938000	6.254161000	3.444316000

H	4.498026000	5.243750000	2.086706000
C	1.778359000	5.176818000	-2.435536000
C	7.449693000	5.063204000	-3.565253000
C	7.725776000	4.364631000	-5.825987000
H	7.334787000	3.952237000	-6.758568000
C	6.875396000	4.538815000	-4.743912000
H	5.819048000	4.268734000	-4.791052000
C	8.834425000	5.396008000	-3.497002000
C	9.107394000	4.704667000	-5.757821000
H	9.728889000	4.545987000	-6.640268000
C	9.684935000	5.225107000	-4.610321000
H	10.745137000	5.473848000	-4.555260000
C	0.942720000	6.280750000	-2.264807000
C	1.176080000	3.923241000	-2.555596000
I	-3.153408000	4.683134000	-2.315800000
F	-0.724649000	2.525156000	-2.627744000
F	-1.198405000	7.246442000	-2.078246000
C	-1.043889000	4.877970000	-2.342286000
C	-0.208251000	3.774040000	-2.513014000
C	-0.441609000	6.131547000	-2.222225000
O	0.697842000	-7.986142000	2.688963000
H	0.719468000	-7.634564000	3.628404000
N	2.918304000	-6.961738000	4.264472000
N	3.940388000	-6.513826000	5.000458000
C	4.296627000	-7.159505000	2.269115000
H	5.121794000	-6.745975000	2.847999000
C	3.402676000	-8.070561000	0.236137000
H	3.530087000	-8.370926000	-0.806277000
C	4.496415000	-7.532133000	0.940723000
C	1.945168000	-7.833756000	2.174502000
C	2.152933000	-8.209440000	0.837587000
H	1.308879000	-8.648982000	0.301312000
C	3.052067000	-7.320287000	2.897245000
N	1.732510000	-7.036010000	4.888180000
C	5.851956000	-7.394016000	0.295785000
H	6.525033000	-8.203019000	0.630213000
H	5.773473000	-7.447080000	-0.799922000
H	6.329102000	-6.436607000	0.557099000
C	3.377152000	-6.255689000	6.208952000
C	3.100268000	-5.557595000	8.470049000
H	3.492057000	-5.144724000	9.402269000
C	3.951449000	-5.731301000	7.387613000
H	5.008370000	-5.461655000	7.435446000
C	1.992417000	-6.588494000	6.140702000

C	1.718649000	-5.897631000	8.401884000
H	1.097155000	-5.738951000	9.284329000
C	1.141910000	-6.417592000	7.254020000
H	0.080908000	-6.666811000	7.199323000
I	-9.790913000	1.960112000	2.472053000
F	-8.147554000	3.248924000	4.979907000
F	-7.224121000	1.835435000	0.511169000
C	-7.762594000	2.511721000	2.737362000
C	-7.307043000	3.073771000	3.929952000
C	-6.839780000	2.352978000	1.703077000
I	-3.023873000	3.849591000	3.308819000
F	-4.667233000	2.560779000	0.800964000
O	3.740553000	2.972405000	3.845127000
H	3.423963000	3.916816000	3.965636000
N	1.041900000	4.048981000	3.987961000
F	-5.590089000	3.973833000	5.270395000
N	-0.196375000	4.550937000	4.028847000
C	0.230602000	1.755436000	3.945745000
H	-0.779527000	2.154561000	4.031502000
C	1.751596000	-0.088558000	3.725439000
H	1.936865000	-1.161923000	3.644294000
C	0.429394000	0.378863000	3.844539000
C	2.640690000	2.178164000	3.834933000
C	2.832694000	0.791294000	3.729991000
H	3.858241000	0.433442000	3.614101000
C	1.307656000	2.656170000	3.922037000
N	2.042374000	4.943568000	4.035464000
C	-0.741264000	-0.569488000	3.825013000
H	-1.233089000	-0.561371000	2.836342000
H	-0.415874000	-1.599343000	4.036056000
H	-1.498940000	-0.292342000	4.574462000
C	-5.052191000	3.297983000	3.043510000
C	0.003765000	5.890214000	4.127887000
C	-0.410485000	8.226641000	4.347601000
H	-1.088226000	9.076235000	4.448216000
C	-0.930266000	6.944290000	4.239165000
H	-2.004659000	6.753493000	4.256868000
C	1.408156000	6.138551000	4.125251000
C	0.992648000	8.472148000	4.338452000
H	1.344395000	9.500746000	4.428101000
C	1.921942000	7.449189000	4.224697000
H	2.995450000	7.637380000	4.229183000
C	-5.507743000	2.735932000	1.850919000
C	-5.975807000	3.456246000	4.078158000

O	-9.394537000	3.261123000	-4.732565000
H	-9.372911000	3.612697000	-3.793121000
N	-7.174069000	4.285538000	-3.157050000
N	-6.151985000	4.733448000	-2.421065000
C	-5.795750000	4.087779000	-5.152410000
H	-4.970578000	4.501300000	-4.573523000
C	-6.689689000	3.176701000	-7.185408000
H	-6.562302000	2.876341000	-8.227799000
C	-5.595939000	3.715124000	-6.480804000
C	-8.147205000	3.413524000	-5.247019000
C	-7.939437000	3.037837000	-6.583926000
H	-8.783493000	2.598291000	-7.120208000
C	-7.040306000	3.926987000	-4.524276000
N	-8.359862000	4.211265000	-2.533343000
C	-4.240418000	3.853260000	-7.125740000
H	-3.567339000	3.044257000	-6.791310000
H	-4.318900000	3.800195000	-8.221444000
H	-3.763279000	4.810664000	-6.864429000
C	-6.715222000	4.991585000	-1.212569000
C	-6.992104000	5.689680000	1.048527000
H	-6.600316000	6.102552000	1.980746000
C	-6.140925000	5.515973000	-0.033909000
H	-5.084002000	5.785619000	0.013923000
C	-8.099954000	4.658780000	-1.280820000
C	-8.373723000	5.349643000	0.980361000
H	-8.995218000	5.508323000	1.862809000
C	-8.950465000	4.829682000	-0.167502000
H	-10.011466000	4.580462000	-0.222199000
I	-7.813624000	-6.727433000	-4.633275000
F	-6.170264000	-5.438621000	-2.125420000
F	-5.246832000	-6.852111000	-6.594159000
C	-5.785305000	-6.175825000	-4.367967000
C	-5.329755000	-5.613773000	-3.175375000
C	-4.862490000	-6.334568000	-5.402252000
O	-1.443003000	-6.138920000	3.408084000
H	-1.464638000	-6.490463000	2.468459000
N	-3.663427000	-7.163380000	1.832594000
N	-4.685513000	-7.611291000	1.096608000
C	-5.041746000	-6.965622000	3.827953000
H	-5.866918000	-7.379143000	3.249067000
C	-4.147808000	-6.054544000	5.860952000
C	-5.240983000	-6.593402000	5.157041000
C	-2.689710000	-6.291819000	3.923265000
C	-2.898062000	-5.915679000	5.259469000

H	-2.053429000	-5.476568000	5.796445000
C	-3.796616000	-6.805265000	3.200513000
N	-2.477060000	-7.089542000	1.209579000
C	-4.121701000	-7.869863000	-0.111194000
C	-3.845618000	-8.568436000	-2.371928000
H	-4.236605000	-8.980830000	-3.304510000
C	-4.696571000	-8.393816000	-1.290546000
H	-5.752920000	-8.663897000	-1.337686000
C	-2.737543000	-7.536619000	-0.043638000
C	-2.464576000	-8.227951000	-2.304460000
H	-1.842502000	-8.387078000	-3.186210000
C	-1.886457000	-7.707960000	-1.156285000
H	-0.825960000	-7.458312000	-1.102180000
I	-1.046584000	-4.837954000	-3.796509000
F	-2.689944000	-6.126766000	-6.304364000
O	5.717845000	-5.715139000	-3.260203000
H	5.401259000	-4.770750000	-3.139708000
N	3.019189000	-4.638564000	-3.117367000
F	-3.612809000	-4.713716000	-1.834928000
N	1.780914000	-4.136609000	-3.076481000
C	2.207891000	-6.932110000	-3.159583000
H	1.197762000	-6.532986000	-3.073826000
C	3.728884000	-8.776104000	-3.379889000
H	3.914163000	-9.849466000	-3.461034000
C	2.406680000	-8.308686000	-3.260789000
C	4.617980000	-6.509383000	-3.270396000
C	4.809981000	-7.896249000	-3.375337000
H	5.835529000	-8.254105000	-3.491228000
C	3.284946000	-6.031378000	-3.183291000
N	4.019663000	-3.743979000	-3.069865000
C	1.235997000	-9.257027000	-3.280305000
H	0.744302000	-9.248951000	-4.269041000
H	1.561434000	-10.286911000	-3.069269000
H	0.478360000	-8.979899000	-2.530844000
C	-3.074903000	-5.389566000	-4.061817000
C	1.981054000	-2.797332000	-2.977441000
C	1.566804000	-0.460906000	-2.757728000
H	0.889062000	0.388689000	-2.657111000
C	1.047023000	-1.743254000	-2.866163000
H	-0.027369000	-1.934053000	-2.848460000
C	3.385445000	-2.548995000	-2.980077000
C	2.969944000	-0.215400000	-2.766886000
H	3.321688000	0.813176000	-2.677234000
C	3.899232000	-1.238359000	-2.880629000

H	4.972776000	-1.050176000	-2.876144000
C	-3.530453000	-5.951613000	-5.254409000
C	-3.998516000	-5.231298000	-3.027169000
I	2.898435000	9.714950000	1.091005000
F	0.470475000	11.873406000	1.402587000
F	0.943406000	7.151593000	0.853411000
C	0.789712000	9.520591000	1.117139000
C	-0.045923000	10.624523000	1.287856000
C	0.187456000	8.267026000	0.997087000
I	-4.142052000	9.026906000	1.236865000
F	-1.713294000	6.868928000	0.924920000
F	-2.187047000	11.590216000	1.474418000
C	-2.032533000	9.221743000	1.210379000
C	-1.196896000	8.117812000	1.039646000
C	-1.430254000	10.475320000	1.330439000
O	11.117653000	2.449893000	-3.597921000
H	11.095227000	2.097841000	-4.537002000
N	8.896385000	1.424999000	-5.173073000
N	7.875099000	0.977567000	-5.909422000
C	7.518065000	1.622758000	-3.177713000
H	6.693694000	1.209715000	-3.756962000
C	8.412804000	2.534314000	-1.145078000
H	8.284618000	2.834196000	-0.102325000
C	7.318257000	1.995399000	-1.849318000
C	9.870321000	2.297492000	-3.083466000
C	9.662551000	2.673179000	-1.746561000
H	10.506609000	3.112727000	-1.210277000
C	8.763421000	1.784028000	-3.806209000
N	10.082978000	1.499751000	-5.797143000
C	5.963427000	1.857592000	-1.204725000
H	5.290510000	2.666776000	-1.539164000
H	6.042511000	1.910683000	-0.108306000
H	5.486389000	0.900315000	-1.466020000
C	8.438337000	0.719430000	-7.117916000
C	8.714420000	0.020857000	-9.378651000
H	8.323432000	-0.391535000	-10.311233000
C	7.864040000	0.195042000	-8.296577000
H	6.807692000	-0.075039000	-8.343716000
C	9.823070000	1.052235000	-7.049666000
C	10.096039000	0.360893000	-9.310485000
H	10.717533000	0.202214000	-10.192932000
C	10.673580000	0.881333000	-8.162984000
H	11.733780000	1.130075000	-8.107924000
O	-0.290809000	-3.642379000	6.241621000

H	-0.269182000	-3.290806000	7.181065000
N	1.929659000	-2.617964000	7.817136000
N	2.951744000	-2.170054000	8.553121000
C	3.307977000	-2.815724000	5.821776000
H	4.133150000	-2.402200000	6.400662000
C	2.413906000	-3.727046000	3.788835000
H	2.541703000	-4.026759000	2.746306000
C	3.507804000	-3.188432000	4.493399000
C	0.956525000	-3.489984000	5.727169000
C	1.164278000	-3.865629000	4.390254000
H	0.320237000	-4.305194000	3.853986000
C	2.063422000	-2.976515000	6.449910000
N	0.743866000	-2.692238000	8.440843000
C	4.863314000	-3.050242000	3.848446000
H	5.536389000	-3.859246000	4.182875000
H	4.784829000	-3.103306000	2.752741000
H	5.340446000	-2.092836000	4.109757000
C	2.388507000	-1.911917000	9.761616000
C	2.111624000	-1.213822000	12.022713000
H	2.503412000	-0.800950000	12.954933000
C	2.962804000	-1.387528000	10.940276000
H	4.019726000	-1.117882000	10.988110000
C	1.003774000	-2.244721000	9.693365000
C	0.730004000	-1.553858000	11.954548000
H	0.108510000	-1.395178000	12.836994000
C	0.153264000	-2.073819000	10.806684000
H	-0.907737000	-2.323039000	10.751987000
O	6.097586000	0.178806000	2.351145000
H	6.414162000	-0.765546000	2.230675000
N	8.796299000	-0.897694000	2.208403000
N	10.034574000	-1.399649000	2.167517000
C	9.607597000	1.395850000	2.250619000
H	10.617727000	0.996725000	2.164862000
C	8.086603000	3.239845000	2.470925000
H	7.901329000	4.313209000	2.552070000
C	9.408806000	2.772423000	2.351825000
C	7.198089000	0.972681000	2.362113000
C	7.005505000	2.359992000	2.466376000
H	5.979957000	2.717846000	2.582264000
C	8.530543000	0.495118000	2.274334000
N	7.795825000	-1.792281000	2.160902000
C	10.579491000	3.720768000	2.371342000
H	11.070386000	3.712214000	3.360440000
H	10.254054000	4.750651000	2.160305000

H	11.337138000	3.443629000	1.621902000
C	9.834209000	-2.739842000	2.069532000
C	10.248684000	-5.075353000	1.848764000
H	10.926425000	-5.924948000	1.748147000
C	10.767665000	-3.793483000	1.957561000
H	11.842058000	-3.602684000	1.939858000
C	8.430043000	-2.987265000	2.071112000
C	8.845552000	-5.320861000	1.857911000
H	8.493805000	-6.349459000	1.768263000
C	7.916831000	-4.298338000	1.972361000
H	6.842750000	-4.486092000	1.967180000
O	7.086253000	-4.164911000	-1.201368000
H	7.402873000	-5.109286000	-1.321930000
N	9.784944000	-5.241466000	-1.344261000
N	11.023218000	-5.743423000	-1.385147000
C	10.596241000	-2.947922000	-1.302046000
H	11.606372000	-3.347047000	-1.387803000
C	9.075247000	-1.103927000	-1.081754000
H	8.889974000	-0.030563000	-1.000595000
C	10.397451000	-1.571349000	-1.200839000
C	8.186720000	-3.371073000	-1.190547000
C	7.994095000	-1.983915000	-1.086348000
H	6.968654000	-1.625737000	-0.970323000
C	9.519188000	-3.848655000	-1.278343000
N	8.784469000	-6.136053000	-1.391762000
C	11.568136000	-0.623004000	-1.181324000
H	12.059031000	-0.631559000	-0.192225000
H	11.242699000	0.406879000	-1.392359000
H	12.325783000	-0.900144000	-1.930763000
C	10.822853000	-7.083616000	-1.483132000
C	11.237329000	-9.419127000	-1.703901000
H	11.915070000	-10.268721000	-1.804516000
C	11.756309000	-8.137256000	-1.595103000
H	12.830701000	-7.946456000	-1.612806000
C	9.418688000	-7.331039000	-1.481551000
C	9.834194000	-9.664634000	-1.694752000
H	9.482450000	-10.693233000	-1.784401000
C	8.905476000	-8.642111000	-1.580303000
H	7.831394000	-8.829865000	-1.585484000
C	-6.611359000	-6.721168000	5.847942000
H	-7.323073000	-6.099315000	5.346282000
H	-6.937139000	-7.739601000	5.808433000
H	-6.526009000	-6.413360000	6.869152000
H	-4.278548000	-5.745639000	6.877015000

UVF (A.C)

S_0

N	0.588243000	-0.950633000	-0.125949000
N	0.036138000	0.247179000	0.019025000
O	-1.774059000	-1.988542000	0.534680000
H	-0.840779000	-2.078253000	0.247024000
N	0.860655000	1.279173000	0.007221000
C	-1.924365000	1.678700000	0.007794000
H	-1.254335000	2.515392000	-0.147028000
C	1.903840000	-0.687477000	-0.246908000
C	-3.869663000	3.266040000	-0.074628000
H	-3.404928000	3.802276000	-0.904613000
H	-4.950207000	3.228978000	-0.238259000
H	-3.675806000	3.839254000	0.832701000
C	4.407986000	0.447202000	-0.510666000
H	5.409376000	0.846612000	-0.621654000
C	4.222806000	-0.967186000	-0.614121000
H	5.076109000	-1.580443000	-0.872955000
C	2.992515000	-1.557877000	-0.461341000
H	2.843548000	-2.629395000	-0.496493000
C	2.074297000	0.714892000	-0.171228000
C	-4.116589000	0.774178000	0.313855000
H	-5.193489000	0.892913000	0.359947000
C	-2.204922000	-0.714221000	0.372902000
C	-1.380258000	0.397773000	0.138534000
C	-3.295257000	1.883110000	0.073652000
C	3.356659000	1.303911000	-0.290693000
H	3.486289000	2.380751000	-0.260919000
C	-3.578559000	-0.490181000	0.478617000
H	-4.220956000	-1.338080000	0.683716000
N	-10.392109000	5.564736000	-0.902640000
N	-9.978111000	6.839890000	-0.781409000
O	-7.953873000	4.796839000	-0.354014000
H	-8.926116000	4.703124000	-0.573743000
N	-10.927002000	7.777588000	-0.760742000
C	-8.188878000	8.483935000	-0.810692000
H	-8.959238000	9.232694000	-0.982634000
C	-11.741331000	5.673483000	-0.969554000
C	-6.393458000	10.266249000	-0.902868000
H	-6.942241000	10.757203000	-1.719956000
H	-5.317792000	10.306421000	-1.129297000
H	-6.578539000	10.854060000	0.010913000
C	-14.385809000	6.498008000	-1.032837000

H	-15.439838000	6.777584000	-1.058561000
C	-14.043438000	5.119179000	-1.150852000
H	-14.847126000	4.393365000	-1.282645000
C	-12.729043000	4.682384000	-1.126201000
H	-12.452791000	3.637475000	-1.243749000
C	-12.074340000	7.058886000	-0.877250000
C	-5.909599000	7.819166000	-0.464373000
H	-4.854144000	8.075372000	-0.376936000
C	-7.647072000	6.117263000	-0.457060000
C	-8.595655000	7.147471000	-0.679568000
C	-6.843132000	8.840117000	-0.723561000
C	-13.420306000	7.485720000	-0.904900000
H	-13.678641000	8.542196000	-0.829674000
C	-6.300818000	6.491970000	-0.327829000
H	-5.571105000	5.707435000	-0.122790000
F	1.505360000	4.186896000	-0.919459000
F	2.586868000	9.564069000	-1.218954000
F	0.006253000	10.083466000	-1.101461000
F	-1.038133000	4.706909000	-0.714253000
F	4.027280000	5.082859000	-1.234106000
F	-3.021125000	6.504649000	-0.592722000
F	4.557609000	7.749043000	-1.347251000
F	-2.514949000	9.171047000	-0.796749000
C	0.899238000	7.846321000	-1.050171000
C	0.624373000	6.428815000	-0.951978000
C	-1.490856000	8.302804000	-0.846790000
C	-0.720594000	6.016465000	-0.797394000
C	3.007060000	5.958802000	-1.162717000
C	1.708547000	5.518168000	-1.015752000
C	-1.753655000	6.925670000	-0.740123000
C	2.249806000	8.251777000	-1.181807000
C	-0.190699000	8.749496000	-0.997831000
C	3.277208000	7.336336000	-1.240299000
N	-10.112251000	-0.504848000	-3.372924000
N	-9.698254000	0.770305000	-3.251693000
O	-7.674018000	-1.272739000	-2.824300000
H	-8.646217000	-1.367386000	-3.044403000
N	-10.647146000	1.708003000	-3.231026000
C	-7.909021000	2.414351000	-3.280975000
H	-8.679380000	3.163109000	-3.452918000
C	-11.461475000	-0.396101000	-3.439837000
C	-6.113534000	4.195789000	-3.373555000
H	-6.662342000	4.686693000	-4.190616000
H	-5.037936000	4.236837000	-3.599581000

H	-6.298661000	4.783487000	-2.459715000
C	-14.105910000	0.427498000	-3.503497000
H	-15.159981000	0.708000000	-3.528845000
C	-13.763537000	-0.951331000	-3.621512000
H	-14.567226000	-1.677145000	-3.753305000
C	-12.449143000	-1.388126000	-3.596861000
H	-12.172892000	-2.433035000	-3.714409000
C	-11.794441000	0.988376000	-3.347911000
C	-5.629695000	1.748652000	-2.935062000
H	-4.574288000	2.005786000	-2.847220000
C	-7.367174000	0.046755000	-2.927704000
C	-8.315798000	1.077886000	-3.149852000
C	-6.563232000	2.769607000	-3.194221000
C	-13.140450000	1.416135000	-3.375184000
H	-13.398784000	2.472612000	-3.299958000
C	-6.020861000	0.421507000	-2.798516000
H	-5.291319000	-0.363179000	-2.593374000
F	1.785216000	-1.882689000	-3.389743000
F	2.866767000	3.493558000	-3.689614000
F	0.286109000	4.013880000	-3.571745000
F	-0.758269000	-1.363566000	-3.185059000
F	4.307179000	-0.987652000	-3.704766000
F	-2.741225000	0.434139000	-3.063382000
F	4.837467000	1.679458000	-3.817534000
F	-2.235092000	3.101463000	-3.267033000
C	1.179139000	1.775810000	-3.520831000
C	0.904275000	0.358314000	-3.422592000
C	-1.210956000	2.232294000	-3.317451000
C	-0.440652000	-0.054052000	-3.268034000
C	3.286960000	-0.111708000	-3.633377000
C	1.988404000	-0.551417000	-3.486035000
C	-1.473759000	0.855155000	-3.210802000
C	2.529705000	2.181267000	-3.652467000
C	0.089200000	2.678985000	-3.468492000
C	3.557107000	1.265826000	-3.710959000
N	0.100351000	5.120812000	2.299679000
N	-0.437338000	6.347348000	2.432551000
O	-2.231946000	4.109540000	2.906457000
H	-1.261746000	4.114537000	2.663210000
N	0.410323000	7.376447000	2.432169000
C	-2.384953000	7.800490000	2.448978000
H	-1.700319000	8.623580000	2.259238000
C	1.429435000	5.366386000	2.199996000
C	-4.354342000	9.390474000	2.402853000

H	-3.879538000	9.933719000	1.573244000
H	-5.433705000	9.320553000	2.202784000
H	-4.208879000	9.995171000	3.312590000
C	3.973804000	6.455988000	2.073252000
H	4.992917000	6.841475000	2.022168000
C	3.771928000	5.049355000	1.961904000
H	4.640931000	4.408993000	1.809640000
C	2.508857000	4.480894000	2.018044000
H	2.338813000	3.413105000	1.905913000
C	1.621703000	6.778622000	2.285883000
C	-2.673931000	5.391396000	2.812733000
C	-1.841040000	6.512595000	2.568485000
C	-3.758458000	8.017756000	2.569370000
C	2.916096000	7.340341000	2.225936000
H	3.068227000	8.417410000	2.297068000
C	-4.048836000	5.627041000	2.975301000
H	-4.688637000	4.772384000	3.197267000
F	-11.283186000	-3.584704000	0.110869000
F	-12.912897000	1.653036000	-0.155839000
F	-10.396583000	2.433743000	-0.100316000
F	-8.801600000	-2.808869000	0.254086000
F	-13.890152000	-2.951685000	-0.141418000
F	-7.008789000	-0.818306000	0.329546000
F	-14.692028000	-0.352631000	-0.237891000
F	-7.788856000	1.783344000	0.141659000
C	-11.056413000	0.116343000	-0.030407000
C	-10.635236000	-1.265596000	0.059317000
C	-8.721022000	0.814234000	0.115169000
C	-9.252925000	-1.538636000	0.180541000
C	-12.962059000	-1.976210000	-0.093861000
C	-11.623250000	-2.281184000	0.021221000
N	-9.925321000	-2.564471000	3.307234000
C	-8.315752000	-0.528796000	0.213654000
N	-9.511354000	-1.288942000	3.427539000
O	-7.487118000	-3.331985000	3.854932000
H	-8.459317000	-3.426633000	3.634828000
C	-12.444071000	0.381914000	-0.128549000
N	-10.460215000	-0.351619000	3.449132000
C	-10.062225000	1.125988000	-0.003561000
C	-7.722122000	0.355103000	3.398256000
H	-8.492480000	1.103862000	3.226314000
C	-13.372971000	-0.633469000	-0.163112000
C	-11.274544000	-2.455724000	3.240320000
C	-5.926659000	2.136492000	3.305704000

H	-6.475442000	2.627446000	2.488615000
H	-4.851036000	2.177590000	3.079651000
H	-6.111740000	2.724303000	4.219484000
C	-13.919010000	-1.631749000	3.175735000
H	-14.973081000	-1.351246000	3.150386000
C	-13.576638000	-3.010578000	3.057719000
H	-14.380327000	-3.736391000	2.925926000
C	-12.262244000	-3.447373000	3.082370000
H	-11.985992000	-4.492282000	2.964822000
C	-11.607510000	-1.071246000	3.332247000
C	-5.442796000	-0.310595000	3.744169000
H	-4.387357000	-0.053836000	3.832938000
C	-7.180274000	-2.012492000	3.751528000
C	-8.128898000	-0.981360000	3.529380000
C	-6.376332000	0.710360000	3.485010000
C	-12.953550000	-0.643112000	3.304047000
H	-13.211853000	0.412990000	3.380200000
C	-5.833931000	-1.638116000	3.881642000
H	-5.104420000	-2.422425000	4.085858000
F	9.140750000	6.688746000	-0.304482000
F	8.059211000	1.311948000	-0.005913000
F	10.639857000	0.792176000	-0.122480000
F	11.684278000	6.168698000	-0.509543000
F	6.618799000	5.793158000	0.009238000
F	13.667234000	4.370992000	-0.631219000
F	6.088473000	3.126974000	0.122381000
F	13.161058000	1.704594000	-0.427192000
C	9.746840000	3.029697000	-0.174696000
C	10.021734000	4.446818000	-0.272009000
C	12.136965000	2.572838000	-0.377151000
C	11.366661000	4.859183000	-0.426568000
C	7.639049000	4.916839000	-0.061222000
C	8.937563000	5.357474000	-0.208189000
N	10.704014000	3.748948000	3.144939000
C	12.399737000	3.950351000	-0.484726000
N	11.241735000	2.522037000	3.012993000
O	13.036234000	4.760167000	2.538889000
H	12.066154000	4.754662000	2.782375000
C	8.396304000	2.623865000	-0.042133000
N	10.394073000	1.492936000	3.013375000
C	10.836778000	2.126522000	-0.227036000
C	13.189350000	1.068893000	2.996567000
H	12.504716000	0.245805000	3.186306000
C	7.368870000	3.539682000	0.015433000

C	9.374930000	3.503375000	3.244614000
C	15.158739000	-0.521089000	3.042691000
H	14.683934000	-1.064335000	3.872301000
H	16.238103000	-0.451170000	3.242761000
H	15.013276000	-1.125787000	2.132954000
C	6.830562000	2.413772000	3.371366000
H	5.811449000	2.028286000	3.422450000
C	7.032470000	3.820029000	3.483651000
H	6.163465000	4.460390000	3.635904000
C	8.295522000	4.388496000	3.427472000
H	8.465655000	5.456210000	3.539560000
C	9.182663000	2.091138000	3.158735000
C	15.379904000	1.962015000	2.595602000
H	16.453487000	1.814948000	2.481167000
C	13.478400000	3.478009000	2.632755000
C	12.645480000	2.355865000	2.876695000
C	14.562823000	0.852004000	2.875248000
C	7.888270000	1.529418000	3.218682000
H	7.736170000	0.451973000	3.148476000
C	14.853305000	3.242652000	2.469355000
H	15.492905000	4.096905000	2.248392000
F	1.972116000	-3.941935000	3.289489000
F	3.053674000	1.434314000	2.989616000
F	0.473009000	1.954634000	3.107486000
F	-0.571369000	-3.422814000	3.494173000
F	4.494079000	-3.046898000	2.974465000
F	-2.554325000	-1.625108000	3.615849000
F	5.024397000	-0.380164000	2.862623000
F	-2.048192000	1.042217000	3.412199000
C	1.366038000	-0.283437000	3.158400000
C	1.091175000	-1.700933000	3.256639000
C	-1.024057000	0.173047000	3.361781000
C	-0.253752000	-2.113299000	3.411198000
C	3.473860000	-2.170955000	3.045854000
C	2.175303000	-2.610664000	3.193196000
C	-1.286828000	-1.204467000	3.469356000
C	2.716603000	0.122021000	3.026767000
C	0.276100000	0.619739000	3.210740000
C	3.744007000	-0.793421000	2.968272000
N	-9.645422000	-8.634981000	0.836573000
N	-9.231455000	-7.359452000	0.956879000
O	-7.207219000	-9.402495000	1.384272000
H	-8.179461000	-9.496217000	1.164544000
N	-10.180316000	-6.422130000	0.978472000

C	-7.442221000	-5.715406000	0.927596000
H	-8.212581000	-4.966648000	0.755654000
C	-10.994645000	-8.526234000	0.769660000
C	-5.646806000	-3.933091000	0.835405000
H	-6.195586000	-3.442153000	0.018324000
H	-4.571138000	-3.892861000	0.608990000
H	-5.831885000	-3.345270000	1.749194000
C	-13.639153000	-7.701334000	0.705451000
H	-14.693182000	-7.421757000	0.679726000
C	-13.296781000	-9.080163000	0.587435000
H	-14.100469000	-9.805976000	0.455642000
C	-11.982387000	-9.516957000	0.612087000
H	-11.706135000	-10.561867000	0.494539000
C	-11.327654000	-7.140831000	0.861964000
C	-5.162939000	-6.380180000	1.273885000
H	-4.107458000	-6.124346000	1.362278000
C	-6.900418000	-8.082077000	1.281244000
C	-7.848999000	-7.051871000	1.058719000
C	-6.096472000	-5.359224000	1.014745000
C	-12.673651000	-6.713622000	0.833387000
H	-12.931954000	-5.657521000	0.909540000
C	-5.554074000	-7.707700000	1.411358000
H	-4.824562000	-8.492010000	1.615574000
F	9.420649000	0.618235000	-2.775142000
F	8.339067000	-4.757637000	-2.476197000
F	10.919756000	-5.278334000	-2.593140000
F	11.964135000	0.099114000	-2.979826000
F	6.898656000	-0.276426000	-2.461045000
F	13.947091000	-1.698592000	-3.101502000
F	6.368483000	-2.943477000	-2.348105000
F	13.440958000	-4.365917000	-2.897852000
C	10.026697000	-3.039888000	-2.644980000
C	10.301591000	-1.622766000	-2.742292000
C	12.416822000	-3.496747000	-2.847434000
C	11.646517000	-1.210401000	-2.896851000
C	7.918906000	-1.152745000	-2.531510000
C	9.217462000	-0.713037000	-2.678849000
N	10.983914000	-2.321562000	0.674279000
C	12.679595000	-2.119233000	-2.955009000
N	11.521635000	-3.548473000	0.542333000
O	13.316133000	-1.310344000	0.068229000
H	12.346010000	-1.314923000	0.312091000
C	8.676158000	-3.445721000	-2.512443000
N	10.673973000	-4.577574000	0.542715000

C	11.116635000	-3.943063000	-2.697320000
C	13.469249000	-5.001617000	0.525906000
H	12.784615000	-5.824705000	0.715646000
C	7.648692000	-2.529810000	-2.454912000
C	9.654830000	-2.567135000	0.773954000
C	15.438596000	-6.590674000	0.572408000
H	14.963791000	-7.133920000	1.402017000
H	16.518002000	-6.521680000	0.772101000
H	15.293133000	-7.195371000	-0.337330000
C	7.110425000	-3.655818000	0.901135000
H	6.091349000	-4.042225000	0.951790000
C	7.312286000	-2.249620000	1.013402000
H	6.443243000	-1.609271000	1.165555000
C	8.575375000	-1.681085000	0.957149000
H	8.745512000	-0.613375000	1.069277000
C	9.462520000	-3.978446000	0.688452000
C	15.659762000	-4.107570000	0.125319000
H	16.733388000	-4.255562000	0.010507000
C	13.758257000	-2.591576000	0.162472000
C	12.925337000	-3.713720000	0.406412000
C	14.842681000	-5.217580000	0.404965000
C	8.168169000	-4.541092000	0.748022000
H	8.016070000	-5.618537000	0.677816000
C	15.133161000	-2.826933000	-0.000929000
H	15.772763000	-1.972681000	-0.221891000
F	2.252016000	-10.012446000	0.818829000
F	3.333496000	-4.635230000	0.519327000
F	0.752908000	-4.115876000	0.636826000
F	-0.291513000	-9.492398000	1.023889000
F	4.773936000	-9.116483000	0.504182000
F	-2.274468000	-7.694692000	1.145565000
F	5.304296000	-6.450674000	0.391963000
F	-1.768293000	-5.028294000	0.941538000
C	1.645897000	-6.353021000	0.688137000
C	1.371032000	-7.770518000	0.786355000
C	-0.744200000	-5.896538000	0.891497000
C	0.026105000	-8.182883000	0.940914000
C	3.753716000	-8.240539000	0.575570000
C	2.455203000	-8.681174000	0.722536000
N	0.688721000	-7.072273000	-2.631519000
C	-1.006972000	-7.274051000	0.999072000
N	0.151030000	-5.845737000	-2.498647000
O	-1.643469000	-8.083867000	-2.024543000
H	-0.673389000	-8.078362000	-2.268028000

C	2.996482000	-5.947571000	0.556451000
N	0.998692000	-4.816636000	-2.499028000
C	0.555957000	-5.449846000	0.740456000
C	-1.796584000	-4.392594000	-2.482220000
H	-1.111950000	-3.569504000	-2.671960000
C	4.023863000	-6.863006000	0.497963000
C	2.017805000	-6.826699000	-2.731194000
C	-3.765956000	-2.802597000	-2.528356000
H	-3.291156000	-2.259372000	-3.357951000
H	-4.845336000	-2.872507000	-2.728430000
H	-3.620524000	-2.197933000	-1.618491000
C	4.562172000	-5.737095000	-2.857949000
H	5.581286000	-5.351611000	-2.909027000
C	4.360295000	-7.143729000	-2.969304000
H	5.229300000	-7.784090000	-3.121558000
C	3.097243000	-7.712196000	-2.913126000
H	2.927110000	-8.779910000	-3.025214000
C	2.210072000	-5.414463000	-2.645314000
C	-3.987139000	-5.285715000	-2.081256000
H	-5.060753000	-5.138272000	-1.967747000
C	-2.085635000	-6.801709000	-2.118409000
C	-1.252673000	-5.680490000	-2.362725000
C	-3.170088000	-4.175329000	-2.361828000
C	3.504462000	-4.852742000	-2.705267000
H	3.656598000	-3.775672000	-2.634115000
C	-3.460570000	-6.565977000	-1.955935000
H	-4.100140000	-7.420605000	-1.734046000
C	-4.595197000	6.908770000	2.742639000
H	-5.657034000	7.033079000	2.698533000

S_1 (enol)

N	0.576521000	-0.955751000	-0.116261000
N	0.016170000	0.295024000	0.038891000
O	-1.704329000	-1.907415000	0.484249000
H	-0.707888000	-1.899402000	0.253926000
N	0.889807000	1.344776000	-0.011691000
C	-1.915966000	1.731253000	0.049177000
H	-1.268571000	2.588598000	-0.073455000
C	1.890462000	-0.689762000	-0.241405000
C	-3.901176000	3.246620000	-0.035642000
H	-3.429856000	3.804933000	-0.848537000
H	-4.981113000	3.205321000	-0.195965000
H	-3.699378000	3.800769000	0.883571000
C	4.421919000	0.418155000	-0.520785000

H	5.425749000	0.811102000	-0.636543000
C	4.222145000	-0.982967000	-0.611186000
H	5.063975000	-1.612897000	-0.868642000
C	2.970710000	-1.560436000	-0.448323000
H	2.813975000	-2.631336000	-0.469534000
C	2.078003000	0.734726000	-0.182146000
C	-4.136287000	0.751625000	0.295828000
H	-5.211840000	0.871089000	0.332446000
C	-2.195156000	-0.692090000	0.341264000
C	-1.335942000	0.463198000	0.130463000
C	-3.304177000	1.882355000	0.092896000
C	3.369010000	1.293058000	-0.304114000
H	3.512090000	2.368579000	-0.282628000
C	-3.584005000	-0.504693000	0.441284000
H	-4.204398000	-1.371448000	0.631263000
N	-10.392630000	5.563370000	-0.902514000
N	-9.978687000	6.838542000	-0.781283000
O	-7.954361000	4.795577000	-0.353888000
H	-8.926601000	4.701821000	-0.573617000
N	-10.927619000	7.776200000	-0.760616000
C	-8.189524000	8.482664000	-0.810566000
H	-8.959917000	9.231389000	-0.982508000
C	-11.741856000	5.672060000	-0.969428000
C	-6.394181000	10.265055000	-0.902742000
H	-6.942986000	10.755985000	-1.719830000
H	-5.318518000	10.305274000	-1.129171000
H	-6.579288000	10.852858000	0.011039000
C	-14.386370000	6.496470000	-1.032711000
H	-15.440411000	6.776002000	-1.058435000
C	-14.043939000	5.117656000	-1.150726000
H	-14.847597000	4.391807000	-1.282519000
C	-12.729526000	4.680917000	-1.126075000
H	-12.453229000	3.636020000	-1.243623000
C	-12.074925000	7.057448000	-0.877124000
C	-5.910217000	7.817992000	-0.464248000
H	-4.854773000	8.074244000	-0.376810000
C	-7.647616000	6.116015000	-0.456933000
C	-8.596244000	7.146183000	-0.679442000
C	-6.843793000	8.838903000	-0.723435000
C	-13.420909000	7.484223000	-0.904774000
H	-13.679290000	8.540689000	-0.829548000
C	-6.301377000	6.490782000	-0.327705000
H	-5.571638000	5.706271000	-0.122660000
F	1.504898000	4.186042000	-0.919333000

F	2.586174000	9.563261000	-1.218828000
F	0.005537000	10.082547000	-1.101335000
F	-1.038616000	4.705947000	-0.714132000
F	4.026779000	5.082113000	-1.233980000
F	-3.021686000	6.503600000	-0.592596000
F	4.556995000	7.748320000	-1.347125000
F	-2.515625000	9.170021000	-0.796623000
C	0.898620000	7.845441000	-1.050045000
C	0.623815000	6.427923000	-0.951851000
C	-1.491495000	8.301821000	-0.846664000
C	-0.721136000	6.015516000	-0.797268000
C	3.006523000	5.958012000	-1.162591000
C	1.708027000	5.517323000	-1.015626000
C	-1.754234000	6.924675000	-0.739997000
C	2.249170000	8.250955000	-1.181681000
C	-0.191357000	8.748569000	-0.997705000
C	3.276611000	7.335558000	-1.240173000
N	-10.112511000	-0.506202000	-3.372798000
N	-9.698569000	0.768968000	-3.251567000
O	-7.674245000	-1.273988000	-2.824174000
H	-8.646440000	-1.368678000	-3.044277000
N	-10.647501000	1.706627000	-3.230900000
C	-7.909406000	2.413091000	-3.280849000
H	-8.679798000	3.161816000	-3.452792000
C	-11.461739000	-0.397513000	-3.439711000
C	-6.113997000	4.194606000	-3.373428000
H	-6.662825000	4.685487000	-4.190490000
H	-5.038400000	4.235702000	-3.599455000
H	-6.299147000	4.782300000	-2.459590000
C	-14.106209000	0.425972000	-3.503371000
H	-15.160293000	0.706429000	-3.528719000
C	-13.763778000	-0.952842000	-3.621386000
H	-14.567435000	-1.678691000	-3.753179000
C	-12.449364000	-1.389581000	-3.596735000
H	-12.173068000	-2.434478000	-3.714283000
C	-11.794765000	0.986950000	-3.347785000
C	-5.630052000	1.747491000	-2.934936000
H	-4.574656000	2.004671000	-2.847094000
C	-7.367457000	0.045519000	-2.927578000
C	-8.316127000	1.076609000	-3.149726000
C	-6.563633000	2.768406000	-3.194095000
C	-13.140792000	1.414650000	-3.375058000
H	-13.399172000	2.471117000	-3.299832000
C	-6.021160000	0.420329000	-2.798390000

H	-5.291584000	-0.364325000	-2.593248000
F	1.785015000	-1.883531000	-3.389617000
F	2.866335000	3.492763000	-3.689488000
F	0.285654000	4.012974000	-3.571619000
F	-0.758491000	-1.364518000	-3.184933000
F	4.306940000	-0.988385000	-3.704640000
F	-2.741526000	0.433102000	-3.063256000
F	4.837113000	1.678747000	-3.817408000
F	-2.235507000	3.100448000	-3.266907000
C	1.178781000	1.774942000	-3.520705000
C	0.903978000	0.357434000	-3.422466000
C	-1.211333000	2.231323000	-3.317325000
C	-0.440931000	-0.054990000	-3.267908000
C	3.286683000	-0.112486000	-3.633251000
C	1.988146000	-0.552251000	-3.485909000
C	-1.474077000	0.854173000	-3.210676000
C	2.529330000	2.180457000	-3.652341000
C	0.088803000	2.678071000	-3.468366000
C	3.556771000	1.265060000	-3.710833000
N	0.099850000	5.119897000	2.299805000
N	-0.437893000	6.346410000	2.432677000
O	-2.232401000	4.108506000	2.906595000
H	-1.262205000	4.113574000	2.663334000
N	0.409724000	7.375546000	2.432295000
C	-2.385571000	7.799469000	2.449104000
H	-1.700972000	8.622588000	2.259364000
C	1.428923000	5.365528000	2.200122000
C	-4.355028000	9.389367000	2.402979000
H	-3.880246000	9.932633000	1.573370000
H	-5.434387000	9.319400000	2.202910000
H	-4.209591000	9.994071000	3.312716000
C	3.973245000	6.455240000	2.073378000
H	4.992341000	6.840770000	2.022294000
C	3.771430000	5.048598000	1.962030000
H	4.640460000	4.408274000	1.809766000
C	2.508384000	4.480083000	2.018171000
H	2.338383000	3.412287000	1.906040000
C	1.621130000	6.777773000	2.286009000
C	-2.674448000	5.390362000	2.812862000
C	-1.841602000	6.511597000	2.568610000
C	-3.759085000	8.016675000	2.569496000
C	2.915499000	7.339548000	2.226062000
H	3.067584000	8.416624000	2.297194000
C	-4.049365000	5.625951000	2.975425000

H	-4.689115000	4.771268000	3.197385000
F	-11.283314000	-3.586108000	0.110995000
F	-12.913250000	1.651561000	-0.155713000
F	-10.396970000	2.432377000	-0.100190000
F	-8.801761000	-2.810167000	0.254212000
F	-13.890307000	-2.953201000	-0.141292000
F	-7.009034000	-0.819525000	0.329673000
F	-14.692295000	-0.354182000	-0.237765000
F	-7.789214000	1.782090000	0.141785000
C	-11.056699000	0.114948000	-0.030281000
C	-10.635463000	-1.266973000	0.059443000
C	-8.721339000	0.812939000	0.115296000
C	-9.253140000	-1.539953000	0.180666000
C	-12.962255000	-1.977687000	-0.093735000
C	-11.623433000	-2.282603000	0.021347000
N	-9.925492000	-2.565817000	3.307360000
C	-8.316011000	-0.530073000	0.213781000
N	-9.511579000	-1.290270000	3.427665000
O	-7.487257000	-3.333226000	3.855058000
H	-8.459451000	-3.427916000	3.634954000
C	-12.444368000	0.380459000	-0.128423000
N	-10.460482000	-0.352989000	3.449258000
C	-10.062555000	1.124636000	-0.003435000
C	-7.722418000	0.353852000	3.398382000
H	-8.492810000	1.102578000	3.226440000
C	-13.373226000	-0.634963000	-0.162986000
C	-11.274719000	-2.457128000	3.240446000
C	-5.927032000	2.135318000	3.305830000
H	-6.475837000	2.626248000	2.488741000
H	-4.851412000	2.176462000	3.079777000
H	-6.112139000	2.723122000	4.219610000
C	-13.919221000	-1.633267000	3.175861000
H	-14.973305000	-1.352810000	3.150512000
C	-13.576790000	-3.012080000	3.057845000
H	-14.380447000	-3.737929000	2.926052000
C	-12.262377000	-3.448819000	3.082496000
H	-11.986080000	-4.493717000	2.964948000
C	-11.607746000	-1.072665000	3.332373000
C	-5.443064000	-0.311747000	3.744295000
H	-4.387636000	-0.054944000	3.833064000
C	-7.180469000	-2.013719000	3.751654000
C	-8.129137000	-0.982629000	3.529506000
C	-6.376644000	0.709167000	3.485136000
C	-12.953803000	-0.644588000	3.304173000

H	-13.212153000	0.411502000	3.380326000
C	-5.834142000	-1.639286000	3.881768000
H	-5.104597000	-2.423564000	4.085984000
F	9.140180000	6.688220000	-0.304356000
F	8.058874000	1.311376000	-0.005787000
F	10.639541000	0.791715000	-0.122354000
F	11.683731000	6.168282000	-0.509417000
F	6.618268000	5.792525000	0.009364000
F	13.666764000	4.370661000	-0.631093000
F	6.088057000	3.126317000	0.122507000
F	13.160703000	1.704241000	-0.427066000
C	9.746428000	3.029198000	-0.174570000
C	10.021261000	4.446330000	-0.271883000
C	12.136573000	2.572441000	-0.377025000
C	11.366171000	4.858753000	-0.426442000
C	7.638556000	4.916249000	-0.061096000
C	8.937051000	5.356940000	-0.208063000
N	10.703571000	3.748489000	3.145065000
C	12.399286000	3.949966000	-0.484600000
N	11.241345000	2.521601000	3.013119000
O	13.035748000	4.759809000	2.539015000
H	12.065668000	4.754262000	2.782501000
C	8.395909000	2.623307000	-0.042007000
N	10.393728000	1.492465000	3.013501000
C	10.836404000	2.126069000	-0.226910000
C	13.189023000	1.068542000	2.996693000
H	12.504424000	0.245424000	3.186432000
C	7.368436000	3.539081000	0.015559000
C	9.374498000	3.502860000	3.244740000
C	15.158480000	-0.521355000	3.042817000
H	14.683698000	-1.064622000	3.872427000
H	16.237840000	-0.451390000	3.242887000
H	15.013043000	-1.126060000	2.133080000
C	6.830177000	2.413146000	3.371492000
H	5.811080000	2.027617000	3.422576000
C	7.032024000	3.819412000	3.483777000
H	6.162991000	4.459735000	3.636030000
C	8.295052000	4.387934000	3.427598000
H	8.465139000	5.455655000	3.539686000
C	9.182292000	2.090614000	3.158861000
C	15.379538000	1.961758000	2.595728000
H	16.453127000	1.814737000	2.481293000
C	13.477969000	3.477670000	2.632881000
C	12.645097000	2.355490000	2.876821000

C	14.562505000	0.851712000	2.875374000
C	7.887922000	1.528839000	3.218808000
H	7.735870000	0.451387000	3.148602000
C	14.852884000	3.242373000	2.469481000
H	15.492448000	4.096653000	2.248518000
F	1.972005000	-3.942769000	3.289615000
F	3.053330000	1.433526000	2.989742000
F	0.472644000	1.953736000	3.107612000
F	-0.571503000	-3.423757000	3.494299000
F	4.493929000	-3.047624000	2.974591000
F	-2.554537000	-1.626137000	3.615975000
F	5.024131000	-0.380866000	2.862749000
F	-2.048518000	1.041209000	3.412325000
C	1.365768000	-0.284297000	3.158526000
C	1.090966000	-1.701805000	3.256765000
C	-1.024346000	0.172084000	3.361907000
C	-0.253943000	-2.114229000	3.411324000
C	3.473672000	-2.171724000	3.045980000
C	2.175134000	-2.611489000	3.193322000
C	-1.287058000	-1.205441000	3.469482000
C	2.716316000	0.121220000	3.026893000
C	0.275792000	0.618832000	3.210866000
C	3.743760000	-0.794179000	2.968398000
N	-9.645332000	-8.636315000	0.836699000
N	-9.231420000	-7.360768000	0.957005000
O	-7.207096000	-9.403724000	1.384398000
H	-8.179333000	-9.497488000	1.164670000
N	-10.180321000	-6.423487000	0.978598000
C	-7.442257000	-5.716645000	0.927722000
H	-8.212649000	-4.967920000	0.755780000
C	-10.994559000	-8.527626000	0.769786000
C	-5.646918000	-3.934254000	0.835535000
H	-6.195720000	-3.443339000	0.018450000
H	-4.571252000	-3.893977000	0.609117000
H	-5.832022000	-3.346440000	1.749319000
C	-13.639104000	-7.702840000	0.705577000
H	-14.693144000	-7.423308000	0.679852000
C	-13.296672000	-9.081654000	0.587561000
H	-14.100328000	-9.807501000	0.455768000
C	-11.982258000	-9.518391000	0.612213000
H	-11.705962000	-10.563290000	0.494665000
C	-11.327628000	-7.142238000	0.862090000
C	-5.162946000	-6.381320000	1.274011000
H	-4.107476000	-6.125442000	1.362404000

C	-6.900352000	-8.083292000	1.281370000
C	-7.848977000	-7.053127000	1.058845000
C	-6.096523000	-5.360406000	1.014870000
C	-12.673643000	-6.715086000	0.833513000
H	-12.931992000	-5.658996000	0.909666000
C	-5.554023000	-7.708858000	1.411484000
H	-4.824478000	-8.493136000	1.615700000
F	9.420341000	0.617722000	-2.775016000
F	8.338991000	-4.758197000	-2.476071000
F	10.919702000	-5.278783000	-2.593014000
F	11.963849000	0.098710000	-2.979700000
F	6.898386000	-0.277048000	-2.460919000
F	13.946882000	-1.698911000	-3.101376000
F	6.368328000	-2.944122000	-2.347979000
F	13.440865000	-4.366257000	-2.897726000
C	10.026546000	-3.040376000	-2.644854000
C	10.301379000	-1.623242000	-2.742166000
C	12.416691000	-3.497132000	-2.847308000
C	11.646288000	-1.210820000	-2.896725000
C	7.918674000	-1.153323000	-2.531384000
C	9.217211000	-0.713558000	-2.678723000
N	10.983732000	-2.322008000	0.674405000
C	12.679404000	-2.119606000	-2.954883000
N	11.521506000	-3.548897000	0.542459000
O	13.315908000	-1.310690000	0.068355000
H	12.345786000	-1.315310000	0.312217000
C	8.676025000	-3.446266000	-2.512317000
N	10.673888000	-4.578033000	0.542841000
C	11.116523000	-3.943504000	-2.697194000
C	13.469183000	-5.001956000	0.526032000
H	12.784585000	-5.825074000	0.715772000
C	7.648520000	-2.530400000	-2.454786000
C	9.654658000	-2.567638000	0.774080000
C	15.438599000	-6.590929000	0.572534000
H	14.963816000	-7.134195000	1.402143000
H	16.518001000	-6.521888000	0.772227000
H	15.293161000	-7.195633000	-0.337204000
C	7.110301000	-3.656431000	0.901261000
H	6.091242000	-4.042882000	0.951916000
C	7.312102000	-2.250224000	1.013528000
H	6.443031000	-1.609913000	1.165682000
C	8.575166000	-1.681634000	0.957276000
H	8.745257000	-0.613918000	1.069403000
C	9.462409000	-3.978958000	0.688578000

C	15.659657000	-4.107815000	0.125445000
H	16.733289000	-4.255761000	0.010633000
C	13.758086000	-2.591902000	0.162598000
C	12.925215000	-3.714083000	0.406538000
C	14.842623000	-5.217861000	0.405091000
C	8.168083000	-4.541659000	0.748148000
H	8.016030000	-5.619111000	0.677942000
C	15.133001000	-2.827200000	-0.000803000
H	15.772566000	-1.972921000	-0.221765000
F	2.252164000	-10.013268000	0.818955000
F	3.333414000	-4.636006000	0.519455000
F	0.752804000	-4.116762000	0.636952000
F	-0.291385000	-9.493329000	1.024015000
F	4.774047000	-9.117197000	0.504308000
F	-2.274419000	-7.695709000	1.145691000
F	5.304292000	-6.451365000	0.392089000
F	-1.768358000	-5.029289000	0.941664000
C	1.645889000	-6.353869000	0.688263000
C	1.371084000	-7.771378000	0.786481000
C	-0.744227000	-5.897489000	0.891623000
C	0.026176000	-8.183801000	0.941040000
C	3.753789000	-8.241297000	0.575696000
C	2.455296000	-8.681987000	0.722662000
N	0.688744000	-7.073162000	-2.631393000
C	-1.006940000	-7.275013000	0.999198000
N	0.151001000	-5.846650000	-2.498521000
O	-1.643402000	-8.084856000	-2.024417000
H	-0.673323000	-8.079309000	-2.267902000
C	2.996457000	-5.948361000	0.556576000
N	0.998618000	-4.817512000	-2.498902000
C	0.555910000	-5.450742000	0.740582000
C	-1.796676000	-4.393589000	-2.482094000
H	-1.112078000	-3.570471000	-2.671834000
C	4.023876000	-6.863752000	0.498089000
C	2.017817000	-6.827531000	-2.731068000
C	-3.766114000	-2.803681000	-2.528229000
H	-3.291339000	-2.260433000	-3.357824000
H	-4.845493000	-2.873634000	-2.728304000
H	-3.620715000	-2.199004000	-1.618372000
C	4.562138000	-5.737818000	-2.857822000
H	5.581235000	-5.352289000	-2.908901000
C	4.360321000	-7.144460000	-2.969178000
H	5.229354000	-7.784784000	-3.121432000
C	3.097294000	-7.712981000	-2.913000000

H	2.927207000	-8.780703000	-3.025088000
C	2.210024000	-5.415287000	-2.645188000
C	-3.987193000	-5.286806000	-2.081130000
H	-5.060813000	-5.139408000	-1.967621000
C	-2.085624000	-6.802717000	-2.118283000
C	-1.252710000	-5.681463000	-2.362599000
C	-3.170190000	-4.176385000	-2.361701000
C	3.504390000	-4.853510000	-2.705140000
H	3.656477000	-3.776434000	-2.633990000
C	-3.460568000	-6.567044000	-1.955809000
H	-4.100102000	-7.421700000	-1.733920000
C	-4.595776000	6.907653000	2.742766000
H	-5.657618000	7.031917000	2.698659000

S_1 (keto)

N	0.579163000	-0.945102000	-0.303039000
N	0.063708000	0.244466000	0.166654000
O	-1.587038000	-1.771062000	0.891240000
H	0.029304000	-1.767133000	0.036181000
N	0.957593000	1.232042000	0.163601000
C	-1.888195000	1.679494000	-0.118100000
H	-1.236194000	2.520643000	-0.319313000
C	1.932438000	-0.755847000	-0.335731000
C	-3.852657000	3.253767000	-0.199622000
H	-3.431277000	3.756675000	-1.073526000
H	-4.942379000	3.243598000	-0.276899000
H	-3.566713000	3.844317000	0.674931000
C	4.467638000	0.324609000	-0.482325000
H	5.476056000	0.714646000	-0.566896000
C	4.250738000	-1.063649000	-0.670078000
H	5.087277000	-1.692209000	-0.941399000
C	2.986665000	-1.630397000	-0.583517000
H	2.818103000	-2.695231000	-0.673373000
C	2.133048000	0.633456000	-0.113539000
C	-4.110926000	0.776440000	0.227527000
H	-5.188023000	0.892672000	0.263690000
C	-2.155567000	-0.677601000	0.515129000
C	-1.354832000	0.455742000	0.154499000
C	-3.301889000	1.869720000	-0.051535000
C	3.430678000	1.188463000	-0.193292000
H	3.589626000	2.254029000	-0.070062000
C	-3.553420000	-0.476580000	0.500633000
H	-4.186310000	-1.307450000	0.789623000
N	-10.396487000	5.563224000	-0.903072000

N	-9.982774000	6.838471000	-0.781841000
O	-7.958080000	4.795870000	-0.354446000
H	-8.930303000	4.701939000	-0.574175000
N	-10.931875000	7.775958000	-0.761174000
C	-8.193907000	8.482915000	-0.811124000
H	-8.964435000	9.231501000	-0.983066000
C	-11.745733000	5.671671000	-0.969986000
C	-6.398885000	10.265629000	-0.903300000
H	-6.947779000	10.756460000	-1.720388000
H	-5.323230000	10.306042000	-1.129729000
H	-6.584098000	10.853399000	0.010481000
C	-14.390395000	6.495604000	-1.033269000
H	-15.444487000	6.774946000	-1.058993000
C	-14.047716000	5.116852000	-1.151284000
H	-14.851243000	4.390858000	-1.283077000
C	-12.733224000	4.680350000	-1.126633000
H	-12.456739000	3.635503000	-1.244181000
C	-12.079051000	7.056999000	-0.877682000
C	-5.914479000	7.818651000	-0.464821000
H	-4.859083000	8.075096000	-0.377368000
C	-7.651574000	6.116365000	-0.457483000
C	-8.600386000	7.146361000	-0.680000000
C	-6.848240000	8.839396000	-0.723993000
C	-13.425112000	7.483531000	-0.905332000
H	-13.683684000	8.539951000	-0.830106000
C	-6.305381000	6.491380000	-0.328310000
H	-5.575566000	5.707005000	-0.123148000
F	1.501289000	4.188040000	-0.919891000
F	2.581596000	9.565454000	-1.219386000
F	0.000865000	10.084275000	-1.101893000
F	-1.042127000	4.707435000	-0.714982000
F	4.023008000	5.084565000	-1.234538000
F	-3.025713000	6.504782000	-0.593154000
F	4.552744000	7.750868000	-1.347683000
F	-2.520132000	9.171294000	-0.797181000
C	0.894351000	7.847330000	-1.050603000
C	0.619797000	6.429760000	-0.952453000
C	-1.495846000	8.303279000	-0.847222000
C	-0.725202000	6.017156000	-0.797633000
C	3.002594000	5.960280000	-1.163149000
C	1.704178000	5.519357000	-1.016184000
C	-1.758337000	6.926085000	-0.740559000
C	2.244828000	8.253087000	-1.182239000
C	-0.195788000	8.750261000	-0.998263000

C	3.272434000	7.337875000	-1.240731000
N	-10.115274000	-0.506297000	-3.373356000
N	-9.701562000	0.768947000	-3.252125000
O	-7.676870000	-1.273644000	-2.824732000
H	-8.649048000	-1.368509000	-3.044835000
N	-10.650663000	1.706435000	-3.231458000
C	-7.912696000	2.413392000	-3.281407000
H	-8.683222000	3.161979000	-3.453350000
C	-11.464522000	-0.397852000	-3.440269000
C	-6.117609000	4.195228000	-3.373984000
H	-6.666524000	4.686013000	-4.191048000
H	-5.042018000	4.236521000	-3.600013000
H	-6.302861000	4.782891000	-2.460148000
C	-14.109140000	0.425157000	-3.503929000
H	-15.163275000	0.705424000	-3.529277000
C	-13.766461000	-0.953595000	-3.621944000
H	-14.569987000	-1.679589000	-3.753737000
C	-12.451968000	-1.390098000	-3.597293000
H	-12.175484000	-2.434945000	-3.714841000
C	-11.797798000	0.986551000	-3.348343000
C	-5.633222000	1.748203000	-2.935494000
H	-4.577872000	2.005573000	-2.847652000
C	-7.370320000	0.045918000	-2.928136000
C	-8.319176000	1.076837000	-3.150284000
C	-6.566987000	2.768950000	-3.194653000
C	-13.143902000	1.414009000	-3.375616000
H	-13.402472000	2.470429000	-3.300390000
C	-6.024091000	0.420971000	-2.798948000
H	-5.294373000	-0.363552000	-2.593806000
F	1.782499000	-1.881483000	-3.390175000
F	2.862851000	3.495006000	-3.690046000
F	0.282076000	4.014752000	-3.572177000
F	-0.761100000	-1.362928000	-3.185491000
F	4.304263000	-0.985882000	-3.705198000
F	-2.744459000	0.434335000	-3.063814000
F	4.833956000	1.681345000	-3.817966000
F	-2.238921000	3.101772000	-3.267465000
C	1.175606000	1.776881000	-3.521263000
C	0.901059000	0.359324000	-3.423024000
C	-1.214590000	2.232831000	-3.317883000
C	-0.443776000	-0.053343000	-3.268466000
C	3.283848000	-0.110167000	-3.633809000
C	1.985391000	-0.550166000	-3.486467000
C	-1.477086000	0.855634000	-3.211234000

C	2.526082000	2.182639000	-3.652899000
C	0.085466000	2.679814000	-3.468924000
C	3.553688000	1.267428000	-3.711391000
N	0.096073000	5.121642000	2.299247000
N	-0.441891000	6.348058000	2.432119000
O	-2.235994000	4.109819000	2.906097000
H	-1.265799000	4.115074000	2.662786000
N	0.405540000	7.377346000	2.431737000
C	-2.389831000	7.800766000	2.448546000
H	-1.705381000	8.624008000	2.258806000
C	1.425101000	5.367512000	2.199564000
C	-4.359575000	9.390309000	2.402421000
H	-3.884891000	9.933660000	1.572812000
H	-5.438921000	9.320147000	2.202352000
H	-4.214247000	9.995039000	3.312158000
C	3.969227000	6.457683000	2.072820000
H	4.988253000	6.843396000	2.021736000
C	3.767664000	5.051002000	1.961473000
H	4.636814000	4.410840000	1.809209000
C	2.504722000	4.482262000	2.017613000
H	2.334913000	3.414435000	1.905482000
C	1.617054000	6.779792000	2.285451000
C	-2.678265000	5.391612000	2.812330000
C	-1.845628000	6.512993000	2.568066000
C	-3.763384000	8.017724000	2.568938000
C	2.911322000	7.341800000	2.225504000
H	3.063212000	8.418903000	2.296636000
C	-4.053235000	5.626947000	2.974857000
H	-4.692830000	4.772150000	3.196827000
F	-11.285523000	-3.586414000	0.110437000
F	-12.916402000	1.650961000	-0.156271000
F	-10.400263000	2.432230000	-0.100748000
F	-8.804109000	-2.810026000	0.253654000
F	-13.892630000	-2.953977000	-0.141850000
F	-7.011750000	-0.819049000	0.329114000
F	-14.695086000	-0.355103000	-0.238323000
F	-7.792390000	1.782413000	0.141227000
C	-11.059574000	0.114682000	-0.030839000
C	-10.638089000	-1.267163000	0.058885000
C	-8.724341000	0.813095000	0.114745000
C	-9.255717000	-1.539894000	0.180100000
C	-12.964753000	-1.978296000	-0.094293000
C	-11.625876000	-2.282971000	0.020789000
N	-9.927884000	-2.565879000	3.306802000

C	-8.318771000	-0.529854000	0.213207000
N	-9.514201000	-1.290257000	3.427107000
O	-7.489511000	-3.332848000	3.854500000
H	-8.461688000	-3.427714000	3.634396000
C	-12.447291000	0.379943000	-0.128981000
N	-10.463273000	-0.353147000	3.448700000
C	-10.065612000	1.124550000	-0.003993000
C	-7.725337000	0.354187000	3.397824000
H	-8.495863000	1.102774000	3.225882000
C	-13.375966000	-0.635646000	-0.163544000
C	-11.277131000	-2.457433000	3.239888000
C	-5.930272000	2.135977000	3.305272000
H	-6.479165000	2.626808000	2.488183000
H	-4.854659000	2.177314000	3.079219000
H	-6.115484000	2.723747000	4.219052000
C	-13.921781000	-1.634048000	3.175303000
H	-14.975916000	-1.353781000	3.149954000
C	-13.579102000	-3.012800000	3.057287000
H	-14.382628000	-3.738793000	2.925494000
C	-12.264610000	-3.449302000	3.081938000
H	-11.988125000	-4.494150000	2.964390000
C	-11.610407000	-1.073030000	3.331815000
C	-5.445863000	-0.311001000	3.743737000
H	-4.390481000	-0.054008000	3.832506000
C	-7.182961000	-2.013286000	3.751096000
C	-8.131815000	-0.982367000	3.528948000
C	-6.379627000	0.709745000	3.484578000
C	-12.956542000	-0.645195000	3.303615000
H	-13.215082000	0.410848000	3.379768000
C	-5.836701000	-1.638611000	3.881210000
H	-5.107015000	-2.422757000	4.085426000
F	9.136120000	6.691594000	-0.304914000
F	8.055783000	1.314555000	-0.006345000
F	10.636543000	0.795359000	-0.122912000
F	11.679764000	6.172114000	-0.509975000
F	6.614369000	5.795444000	0.008806000
F	13.663121000	4.374850000	-0.631651000
F	6.084647000	3.129150000	0.121976000
F	13.157541000	1.708339000	-0.427624000
C	9.743027000	3.032681000	-0.175128000
C	10.017605000	4.449862000	-0.272441000
C	12.133254000	2.576355000	-0.377583000
C	11.362440000	4.862528000	-0.427000000
C	7.634815000	4.919352000	-0.061655000

C	8.933231000	5.360277000	-0.208621000
N	10.700040000	3.752144000	3.144507000
C	12.395719000	3.953927000	-0.485158000
N	11.238036000	2.525353000	3.012561000
O	13.032035000	4.763885000	2.538457000
H	12.061956000	4.758163000	2.781943000
C	8.392583000	2.626547000	-0.042560000
N	10.390604000	1.496065000	3.012943000
C	10.833166000	2.129748000	-0.227468000
C	13.185975000	1.072645000	2.996135000
H	12.501525000	0.249404000	3.185874000
C	7.364941000	3.542138000	0.014985000
C	9.371012000	3.506276000	3.244182000
C	15.155719000	-0.516897000	3.042259000
H	14.681035000	-1.060249000	3.871869000
H	16.235066000	-0.446737000	3.242329000
H	15.010391000	-1.121628000	2.132522000
C	6.826887000	2.416103000	3.370934000
H	5.807860000	2.030391000	3.422018000
C	7.028481000	3.822406000	3.483219000
H	6.159332000	4.462572000	3.635472000
C	8.291406000	4.391155000	3.427040000
H	8.461301000	5.458907000	3.539128000
C	9.179060000	2.093995000	3.158303000
C	15.376329000	1.966256000	2.595170000
H	16.449945000	1.819428000	2.480735000
C	13.474487000	3.481825000	2.632323000
C	12.641817000	2.359495000	2.876263000
C	14.559496000	0.856063000	2.874816000
C	7.884792000	1.531987000	3.218250000
H	7.732934000	0.454508000	3.148044000
C	14.849445000	3.246776000	2.468923000
H	15.488855000	4.101171000	2.247960000
F	1.969861000	-3.940687000	3.289057000
F	3.050217000	1.435803000	2.989184000
F	0.469437000	1.955548000	3.107054000
F	-0.573741000	-3.422133000	3.493741000
F	4.491623000	-3.045087000	2.974033000
F	-2.557099000	-1.624871000	3.615417000
F	5.021345000	-0.378234000	2.862191000
F	-2.051560000	1.042567000	3.411767000
C	1.362964000	-0.282324000	3.157968000
C	1.088418000	-1.699882000	3.256207000
C	-1.027232000	0.173626000	3.361349000

C	-0.256417000	-2.112548000	3.410766000
C	3.471208000	-2.169371000	3.045422000
C	2.172750000	-2.609370000	3.192764000
C	-1.289696000	-1.203946000	3.468924000
C	2.713439000	0.123436000	3.026335000
C	0.272826000	0.620608000	3.210308000
C	3.741048000	-0.791778000	2.967840000
N	-9.646631000	-8.636326000	0.836141000
N	-9.232948000	-7.360705000	0.956447000
O	-7.208256000	-9.403296000	1.383840000
H	-8.180476000	-9.497235000	1.164112000
N	-10.182018000	-6.423595000	0.978040000
C	-7.444082000	-5.716259000	0.927164000
H	-8.214609000	-4.967673000	0.755222000
C	-10.995877000	-8.527880000	0.769228000
C	-5.649059000	-3.933570000	0.834994000
H	-6.197955000	-3.442743000	0.017884000
H	-4.573400000	-3.893054000	0.608562000
H	-5.834275000	-3.345755000	1.748755000
C	-13.640571000	-7.703571000	0.705019000
H	-14.694661000	-7.424229000	0.679294000
C	-13.297890000	-9.082323000	0.587003000
H	-14.101415000	-9.808315000	0.455210000
C	-11.983398000	-9.518823000	0.611655000
H	-11.706913000	-10.563672000	0.494107000
C	-11.329196000	-7.142552000	0.861532000
C	-5.164651000	-6.380523000	1.273453000
H	-4.109227000	-6.124455000	1.361846000
C	-6.901750000	-8.082809000	1.280812000
C	-7.850561000	-7.052814000	1.058287000
C	-6.098408000	-5.359777000	1.014329000
C	-12.675288000	-6.715643000	0.832955000
H	-12.933827000	-5.659599000	0.909108000
C	-5.555489000	-7.708132000	1.410926000
H	-4.825802000	-8.492278000	1.615142000
F	9.417375000	0.621146000	-2.775574000
F	8.336993000	-4.754968000	-2.476629000
F	10.917798000	-5.275089000	-2.593572000
F	11.960976000	0.102593000	-2.980258000
F	6.895581000	-0.274078000	-2.461477000
F	13.944333000	-1.694671000	-3.101934000
F	6.365993000	-2.941231000	-2.348541000
F	13.438797000	-4.362108000	-2.898284000
C	10.024239000	-3.036843000	-2.645412000

C	10.298816000	-1.619659000	-2.742724000
C	12.414466000	-3.493168000	-2.847866000
C	11.643651000	-1.206995000	-2.897283000
C	7.916027000	-1.150169000	-2.531943000
C	9.214485000	-0.710170000	-2.679281000
N	10.981295000	-2.318302000	0.673847000
C	12.676931000	-2.115595000	-2.955441000
N	11.519290000	-3.545094000	0.541901000
O	13.313289000	-1.306564000	0.067797000
H	12.343168000	-1.311359000	0.311659000
C	8.673791000	-3.442976000	-2.512870000
N	10.671858000	-4.574383000	0.542283000
C	11.114379000	-3.939774000	-2.697752000
C	13.467229000	-4.997802000	0.525474000
H	12.782780000	-5.821043000	0.715214000
C	7.646122000	-2.527300000	-2.455345000
C	9.652266000	-2.564172000	0.773522000
C	15.436932000	-6.586420000	0.571976000
H	14.962246000	-7.129772000	1.401585000
H	16.516321000	-6.517185000	0.771669000
H	15.291602000	-7.191150000	-0.337762000
C	7.108105000	-3.653423000	0.900704000
H	6.089116000	-4.040058000	0.951358000
C	7.309653000	-2.247178000	1.012968000
H	6.440462000	-1.607030000	1.165122000
C	8.572614000	-1.678362000	0.956718000
H	8.742513000	-0.610615000	1.068845000
C	9.460271000	-3.975526000	0.688020000
C	15.657542000	-4.103266000	0.124887000
H	16.731201000	-4.251019000	0.010075000
C	13.755698000	-2.587696000	0.162040000
C	12.923029000	-3.710027000	0.405980000
C	14.840708000	-5.213460000	0.404533000
C	8.166046000	-4.538460000	0.747590000
H	8.014188000	-5.615940000	0.677384000
C	15.130655000	-2.822746000	-0.001361000
H	15.770066000	-1.968352000	-0.222323000
F	2.251113000	-10.011135000	0.818397000
F	3.331291000	-4.633668000	0.518974000
F	0.750691000	-4.114900000	0.636394000
F	-0.292529000	-9.491655000	1.023457000
F	4.772835000	-9.114610000	0.503750000
F	-2.275887000	-7.694392000	1.145133000
F	5.302600000	-6.448682000	0.391531000

F	-1.770307000	-5.027881000	0.941106000
C	1.644181000	-6.351846000	0.687725000
C	1.369629000	-7.769404000	0.785923000
C	-0.746019000	-5.895896000	0.891065000
C	0.024796000	-8.182069000	0.940482000
C	3.752419000	-8.238894000	0.575138000
C	2.454006000	-8.679818000	0.722104000
N	0.687164000	-7.071311000	-2.631951000
C	-1.008484000	-7.273468000	0.998640000
N	0.149200000	-5.844896000	-2.499079000
O	-1.644800000	-8.083425000	-2.024975000
H	-0.674722000	-8.077703000	-2.268460000
C	2.994758000	-5.946118000	0.555940000
N	0.996631000	-4.815605000	-2.499460000
C	0.554037000	-5.448915000	0.740024000
C	-1.798739000	-4.392186000	-2.482652000
H	-1.114289000	-3.568945000	-2.672392000
C	4.022257000	-6.861300000	0.497520000
C	2.016192000	-6.825441000	-2.731626000
C	-3.768503000	-2.802569000	-2.528823000
H	-3.293770000	-2.259309000	-3.358378000
H	-4.847831000	-2.872789000	-2.728856000
H	-3.623159000	-2.197977000	-1.618911000
C	4.560317000	-5.735269000	-2.858376000
H	5.579345000	-5.349557000	-2.909457000
C	4.358753000	-7.141947000	-2.969736000
H	5.227902000	-7.782115000	-3.121990000
C	3.095829000	-7.710696000	-2.913558000
H	2.925934000	-8.778449000	-3.025646000
C	2.208144000	-5.413161000	-2.645753000
C	-3.989095000	-5.285798000	-2.081688000
H	-5.062742000	-5.138593000	-1.968179000
C	-2.087253000	-6.801366000	-2.118841000
C	-1.254541000	-5.679963000	-2.363157000
C	-3.172292000	-4.175229000	-2.362259000
C	3.502441000	-4.851157000	-2.705686000
H	3.654249000	-3.774040000	-2.634538000
C	-3.462239000	-6.565941000	-1.956367000
H	-4.101619000	-7.420712000	-1.734478000
C	-4.599876000	6.908551000	2.742208000
H	-5.661740000	7.032624000	2.698101000
